# High‐Performance Phthalonitrile Resins Partially Derived from a Furan Bio‐Based Chemical Platform

**DOI:** 10.1002/cssc.202501854

**Published:** 2025-10-31

**Authors:** Daria I. Poliakova, Sergey S. Nechausov, Daria S. Stepaniuk, Doreen Mollenhauer, Ulrich S. Schubert, Boris A. Bulgakov

**Affiliations:** ^1^ Laboratory of Organic Chemistry and Macromolecular Chemistry (IOMC) Friedrich Schiller University Jena Humboldtstr.10 07743 Jena Germany; ^2^ HIPOLE Jena (Helmholtz Institute for Polymers in Energy Applications Jena) Lessingstrasse 12‐14 07743 Jena Germany; ^3^ Institute for Technical and Environmental Chemistry (ITUC) Friedrich Schiller University Jena Philosophenweg 7a 07743 Jena Germany; ^4^ Helmholtz‐Zentrum Berlin für Materialien und Energie GmbH (HZB) Hahn‐Meitner‐Platz 1 14109 Berlin Germany; ^5^ Jena Center for Soft Matter (JCSM) Friedrich Schiller University Jena Philosophenweg 7 07743 Jena Germany

**Keywords:** bond dissociation energies, density functional theory, furanic platform chemicals, heat resistance, phthalonitriles, sustainability, thermosets

## Abstract

Phthalonitrile resins are renowned for their outstanding heat resistance, withstanding temperatures exceeding 350 °C. This makes them highly sought after in industries such as aerospace, automotive, electronics, and renewable energy. To improve the sustainability of these materials, bio‐derived phenols have been employed for the synthesis of the monomer in this research field. Thermosets derived from these partially bio‐based monomers have demonstrated performance on par with their fossil‐based counterparts, emphasizing the potential of sustainable phthalonitrile resins for advanced applications. Herein, the furan chemical platform is utilized to design and synthesize phthalonitrile monomers. Starting with furfural and its derivatives, novel low‐melting (50–75 °C) Schiff‐base monomers are synthesized and subsequently cured into thermosets. The resulting thermosets exhibit remarkable properties, including degradation temperatures (*T*_5%_, determined by thermogravimetric analysis) above 450 °C (after curing at 350 °C), char yields (*Y*_c_, at 900 °C) determined between 74 and 78%, and glass transition temperatures (*T*_g_) surpassing 400 °C after curing at 350 °C and exceeding 300 °C after curing at only 250 °C. These findings underscore the versatility of furan chemistry in producing more sustainable phthalonitrile resins. The chemical design of these monomers enables the optimization of processability and material properties, broadening their application potential and advancing sustainability across multiple industries.

## Graphical abstract

Low‐melting Schiff‐base phthalonitrile resins are synthesized from a furan‐based chemical platform, providing improved processability and sustainability. Upon curing, the resins yield high‐performance thermosets with exceptional thermal stability, high char yield, and glass transition temperatures above 400 °C.

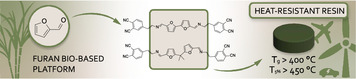

## 1 Introduction

Modern technological challenges demand the development of new materials. The transition to carbon‐neutral industries requires not only the use of bio‐based feedstocks and environmentally friendly processes, but also the implementation of high‐performance, long‐lifespan materials. These advanced materials align with the “Reduce” and “Reuse” principles of the 4R framework,^[^ ^1^ ^]^ supporting sustainable development through a mindful approach to part fabrication. Although most thermosetting materials are not recyclable, they remain essential in high‐end applications due to their exceptional performance characteristics. For example, fiber‐reinforced epoxy resins have transformed material selection in the aerospace industry. Their superior specific strength compared to metals and alloys has enabled significant reductions in aircraft weight and has extended the service life of components, thanks to excellent fatigue and environmental resistance.^[^ ^2^ ^]^ For similar reasons, thermosetting composites play a critical role in electric vehicles,^[^ ^3^ ^]^ wind turbines,^[^ ^4^ ^,^ ^5^ ^]^ high‐voltage insulation systems,^[^ ^6^, ^7^, ^8^ ^]^ and other applications that support sustainability goals. Phthalonitrile resins are distinguished among thermosetting polymers for their outstanding thermal resistance,^[^ ^9^, ^10^, ^11^, ^12^, ^13^ ^]^ extraordinary flame retardancy,^[^ ^14^, ^15^, ^16^, ^17^, ^18^ ^]^ resistance to gamma rays,^[^ ^19^, ^20^ ^]^ low dielectric constants,^[^ ^7^, ^21^ ^]^ and high char yield^[^ ^22^, ^23^ ^]^ and are in high demand for advanced applications ranging from aerospace^[^ ^24^, ^25^ ^]^ to electronics^[^ ^6^ ^]^ and semiconductor technologies.^[^ ^26^, ^27^ ^]^ But one of the primary challenges associated with phthalonitrile resins remains their inherently poor processability. Recent research over the past decade has primarily focused on lowering its melting point,^[^ ^13^, ^28^, ^29^, ^30^ ^]^ enhancing the rheological behavior,^[^ ^28^, ^31^ ^]^ reducing curing times,^[^ ^16^, ^33^ ^]^ and elucidating oxidative degradation mechanisms under high‐temperature exposure,^[^ ^33^, ^34^, ^35^ ^]^ primarily through the incorporation of various chemical linkers. Since the production of phthalonitrile emerged in several countries, the question of sustainability was naturally raised.^[^ ^36^ ^]^ Multiple recent publications report the use of bio‐based feedstock materials for synthesizing thermosetting resins,^[^ ^37^ ^]^ including phthalonitrile resins.^[^ ^38^, ^39^, ^40^, ^41^, ^42^ ^]^ Studies on bio‐based phthalonitriles have mainly focused on the use of plant‐derived phenols, such as vanillin,^[^ ^11^, ^41^ ^]^ resveratrol,^[^ ^43^ ^]^ and eugenol,^[^ ^44^ ^]^ as well as nucleic and amino acid derivatives.^[^ ^45^, ^46^ ^]^ Although the resulting thermosets typically exhibit thermal performance comparable to their fossil‐based counterparts, such as a high decomposition temperature (*T*_5%_ > 450 °C) and a glass transition temperature above 400 °C, resins that combine synthesis from industrially produced bio‐based raw materials with good processability and high thermal and mechanical performance have not yet been reported to the best of our knowledge. Therefore, the exploration of novel bio‐derived raw materials for designing new molecular architectures remains an attractive challenge for researchers.^[^ ^36^ ^]^ As a result of these studies, structure–performance relationships will be established with the aim of formulating basic principles for designing high‐performance bio‐based materials, which can be applied to a wide range of polymers and thermosets.

**Scheme 1:**
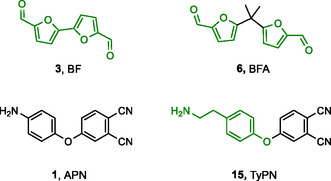
Schematic representation of the building blocks for phthalonitrile diimines synthesized in this work. Bio‐originated fragments of the molecules are highlighted in green.

To address the challenge of limited bio‐feedstock, the present study explores the integration of furanic platform chemicals to enhance the environmental profile of phthalonitrile resins. Furan and its derivatives are increasingly recognized as valuable and versatile chemical platforms due to their unique aromatic structure and renewable origin from biomass.^[^ ^47^ ^]^ Aside from their established applications, furan derivatives serve as essential building blocks for polymers^[^ ^48^, ^49^, ^50^ ^]^ and resins,^[^ ^51^, ^52^ ^]^ since furfural is already produced industrially from biomass in several countries including China, Dominican Republic, and South Africa.^[^ ^53^ ^]^ As a result, furfural and its derivatives can be considered promising for designing novel architectures of bisphthalonitrile monomers. As a fundamental compound within the furan platform,^[^ ^54^ ^]^ furfural possesses a chemical structure that readily facilitates the formation of imine bonds, which are known for their thermal stability—making them highly suitable for applications in heat‐resistant polymers,^[^ ^38^, ^55^ ^]^ including phthalonitrile resins.^[^ ^38^, ^42^ ^]^ In addition, bio‐based amines such as tyramine^[^ ^45^, ^56^ ^]^ can be employed to further improve the processability of the system, while contributing to the sustainability of the material. This work aims to investigate the potential of incorporating furan‐derived imine linkers into phthalonitrile networks for the synthesis of high‐performance, thermally stable thermosetting materials, with the goal of developing resins that combine good processability and high thermal performance with enhanced environmental sustainability. In this context, the present study seeks to ultimately demonstrate the suitability of furan derivatives as a feedstock for phthalonitrile resins and to reveal the first structure–performance relationships as a basis for further research. We selected two furan‐based dialdehydes—bis‐furfural (BF) and 5,5′‐(propane‐2,2‐diyl)bis(furan‐2‐carbaldehyde) (BFA)—along with two phthalonitrile‐bearing amines—4‐(4‐aminophenoxy)phthalonitrile (APN)^[^ ^57^ ^]^ and the partially bio‐based 4‐(4‐(2‐aminoethyl)phenoxy)phthalonitrile (TyPN)^[^ ^56^ ^]^—to synthesize four diimine‐linked bis‐phthalonitrile monomers and investigate their potential as high‐performance thermosetting resins (**Scheme** **1**).

## 2 Results and Discussion

### 2.1 Synthesis and Characterization of Schiff‐Base Phthalonitrile Monomers

**Scheme 2:**
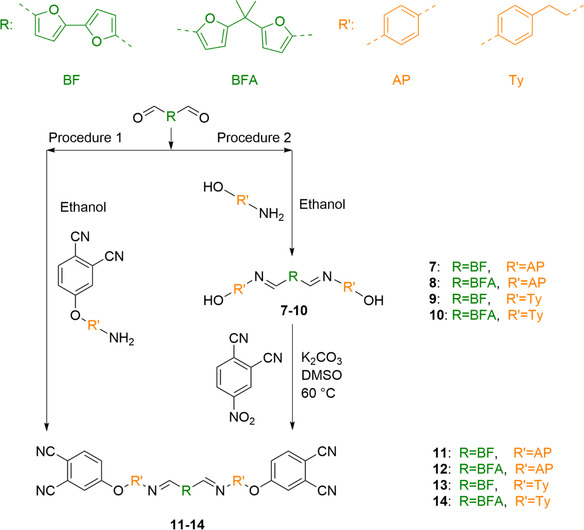
Schematic representation of the general synthesis procedures of the target monomers.

To explore the furan chemistry platform for synthesizing phthalonitrile monomers, furan‐based dialdehydes and aromatic amines bearing phthalonitrile groups were selected for synthesis of the monomers. **Scheme** **2** illustrates the chosen synthetic pathway for phthalonitrile monomers with diimine linkages. These monomers are based on two dialdehydes: Bisfurfural (BF), synthesized via oxidative homocoupling of 2‐furyl‐1,3‐dioxolane, and 5,5′‐(propane‐2,2′‐diyl)difuran‐2‐carbaldehyde (BFA), which is obtained through the Friedel–Crafts alkylation of 2‐furyl‐1,3‐dithiolane. Both dialdehydes were synthesized according to literature procedures^[^ ^55^, ^58^ ^]^ with minor modifications, which are detailed in the Supporting Information. In the synthesis of BF, the conversion of oxidative homocoupling in the presence of palladium acetate catalyst did not exceed 50%, resulting in a relatively low isolated yield. However, a key advantage of this approach is that the homocoupling reaction and deprotection of the aldehyde group can be performed in a one‐pot process. In contrast, the synthesis of BFA requires the isolation of an intermediate alkylation product before deprotection stage, adding a step to the process.

Two methods for obtaining target monomers **11**–**14** (**Table** **1**) from BF and BFA were tested. Direct reaction between BF and 4‐(aminophenoxy)phthalonitrile^[^ ^57^ ^]^ (APN) was carried out in two environmentally friendly solvents: DMSO and ethanol. Conversion of the reaction was estimated by integrating signals on the ^1^H NMR of the reaction mixture, corresponding to the starting materials: The calculations were based on the integration of the most distinguishable signals corresponding to the two protons of APN (6.85 ppm) and the two protons of the product **11** (7.83 ppm) (Figure S42, Supporting Information). After 1 h at 150 °C in a microwave oven, equilibrium was reached for the reaction in both solvents, which remained unchanged even after 6 h at 150 °C. The conversion was up to 80% for the reaction in DMSO and 55% for the reaction in ethanol. A similar effect was observed in the reaction between BFA and Ty‐PN in ethanol, where the reaction mixture also contained the target product, an intermediate, and the unreacted starting materials (Figure S43, Supporting Information). Ty‐PN for this synthesis was prepared according to the procedure reported by Gang Yang et al.,^[^ ^56^ ^]^ and it was expected to obtain a liquid substance. Unexpectedly, the reaction yielded a yellow amorphous substance that solidified upon standing overnight. After additional purification through dissolving the product in DMF followed by precipitation into CH_2_Cl_2,_ a white powder was obtained. The product was characterized by using ^1^H and ^13^C NMR, DSC and thermogravimetric analysis (TGA), and DSC revealed melting point at around 270 °C and the exothermal peak at 285 °C which was attributed to self‐curing of the monomer in Yang's report. Nevertheless, the reasons for this mismatch in behavior were beyond the scope of this study, and Ty‐PN was excluded from further synthesis due to its inability to achieve full conversion in the direct reaction.

**Table 1:** Schematic representation of the list of monomers with their melting points of the monomers 11‐14.

Name	Structure	T_1000_ [°C]a)	T_mp_ [°C]	T_g_ [°C]
11 BF‐AP‐PN	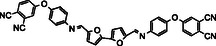	–	251	–
12 BFA‐AP‐PN	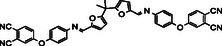	170	–	75
13 BF‐Ty‐PN	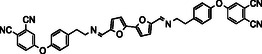	175	–	65
14 BFA‐Ty‐PN	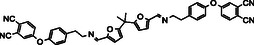	141	–	51

a) Temperature of reaching 1000 mPa·s viscosity.

Since the full conversion was not reached in the direct reaction, the second approach involving a two‐step synthesis was proposed. The intermediate products (**7**–**10**) were first obtained, followed by a nucleophilic substitution reaction with 4‐nitrophthalonitrile similar to the strategy reported by Gang Yang.^[^ ^38^ ^]^ Four intermediate imines (**7**‐**10**, Table 1) were synthesized from BF and BFA via a condensation reaction with 4‐aminophenol (**AP**) or tyramine (**Ty**). The reaction proceeds in ethanol and requires only the dissolution of both reagents to occur. To initiate the process, the ethanol mixture is shortly brought to a boil to ensure complete dissolution, after which the solution is allowed to cool in a refrigerator, leading to the precipitation of the desired product. The reactions were completed in less than 1 h with high yields. This efficiency was facilitated using ethanol as a mildly acidic solvent, which acts as an acid catalyst in the condensation process.

Target phthalonitrile substances with imine linkers (**11**–**14**, Table 1) were synthesized from **7**–**10** and 4‐nitrophthalonitrile via nucleophilic substitution under basic conditions. For this type of reaction, nonpolar aprotic solvents such as DMF and DMAc are typically well‐suited.^[^ ^16^, ^32^ ^]^ The synthesis was optimized with a focus on using nontoxic and environmentally friendly solvents, such as DMSO. The reaction was carried out under an inert atmosphere for 2 h after the addition of 4‐nitrophthalonitrile, after which the product was isolated by precipitation in water. At this step, hydrolysis of the imine bond in the monomers **11** and **12** was a concern (from here and further substances will be called according to building blocks, from which they were synthesized (Table 1, Scheme 2). As previously demonstrated, the reaction between BF and APN is an equilibrium process, which means that in the presence of water, the monomer might be prone to hydrolysis. In the ^1^H NMR spectrum after isolation of the **11** and **12**, signals corresponding to APN are observed in amounts of up to 3 mol%, whereas these signals are absent in the spectrum prior to product isolation (Figure S22 and S27, Supporting Information). In contrast, monomers synthesized from tyramine (**Ty**) do not exhibit hydrolysis during isolation. The increased hydrolytic stability of these monomers may be attributed to the electron‐donating effect of the aliphatic bridge and the interruption of conjugation between the amino group and the aromatic ring, which together enhance the stability of the imine bond compared to monomers containing the AP moiety.^[^ ^59^, ^60^ ^]^ As an alternative to product isolation via precipitation in water, the product could be isolated by evaporating the solvent (DMSO). Due to the high boiling point of DMSO, its complete removal from the final product is highly challenging, even under vacuum and heating conditions (< 0.05 mbar, 120 °C). Since residual solvent can cause porosity formation during curing and affect the properties of the thermosets, it was decided to study the obtained mixture, which contains up to 3 mol% of APN and aldehyde impurities in **11** and **12**. It is expected that during the curing of phthalonitriles, APN will also act as a curing agent, while the aldehyde will be incorporated into the polyisoindoline structures via nucleophilic addition or condensation reaction.^[^ ^61^, ^62^ ^]^ Thus, four novel partially bio‐based monomers were synthesized from furfural, and the subsequent study focused on their curing behavior and the characterization of the resulting thermosets.

### 2.2 Processability Study

**Figure 1:**
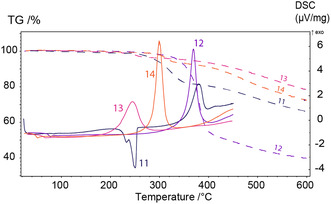
DSC (solid curves) and TGA (dashed curves) of the monomers **11**–**14**.

**Figure 2:**
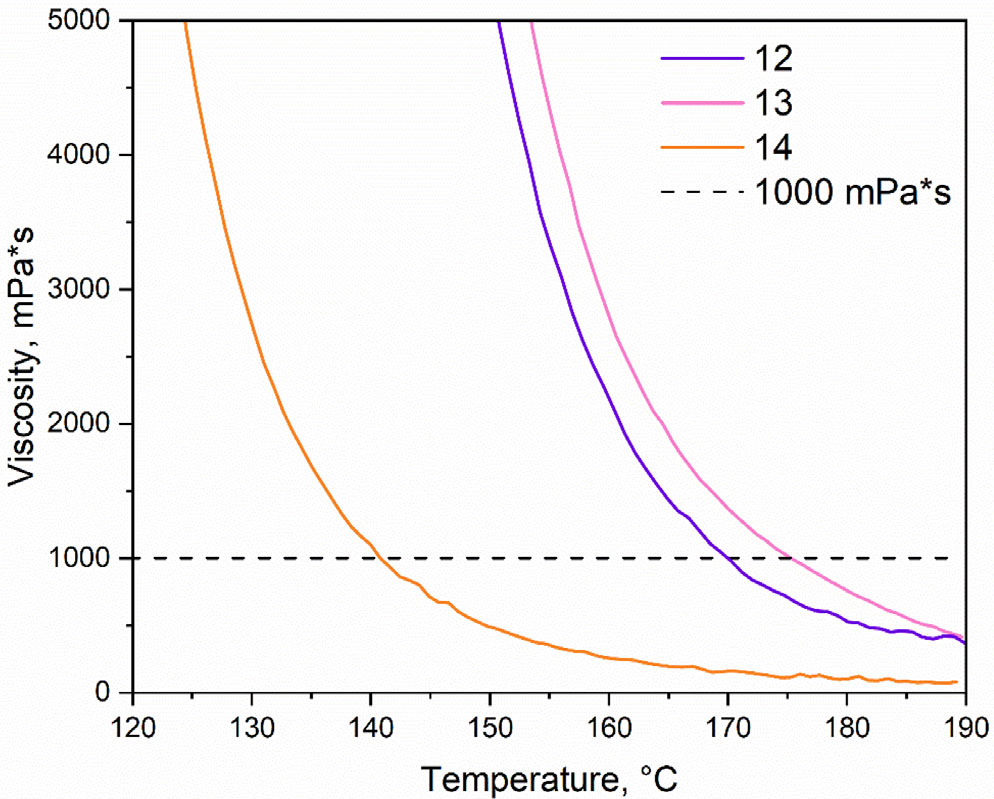
Temperature‐dependent viscosity curves of **12**, **13**, and **14**.

For the curing of phthalonitrile resins and ensuring good processability in commercial applications, a two‐step curing process is typically used—the first stage at 180–200 °C and the second at 280–350 °C.^[^ ^13^, ^36^, ^63^ ^]^ To enable the initial curing stage, the resin should have a melting temperature below 180 °C. For cost‐efficient processing methods such as RTM or VIMP, low viscosity at temperatures below 100 °C is also highly desirable.^[^ ^31^ ^]^ Therefore, reducing the melting temperature is a critical consideration in the design of phthalonitrile monomers. Differential scanning calorimetry (DSC, **Figure** **1**) analysis revealed that only one of the synthesized monomers **11** exhibits a crystalline structure, characterized by a high melting temperature (251 °C). Because of this reason, further processability investigation of **11**, including viscosity measurements, is not possible due to a fast polymerization occurring right after melting. Monomers **12**–**14**, with an iso‐propenyl bridge between the furan rings and/or by an aliphatic bridge in the tyramine‐based structures which increase conformational flexibility of the molecules, were amorphous, and the corresponding DSC curves exhibit only glass transition temperatures, ranging from 50 to 75 °C (Table 1, Figure 1). Despite the relatively low glass transition temperatures of the monomers BF‐Ty‐PN and BFA‐Ty‐PN, the viscosities began to decrease only after 100 °C, reaching a plateau approximately at 170 °C 430 mPa·s for both **12** and **13**, and 250 mPa s for **14** (**Figure** **2**). It has been reported^[^ ^22^, ^28^ ^]^ that monomers with viscosities around 1000 mPa s at 120–150 °C have been successfully used for vacuum infusion in fiber reinforced polymers. The monomer **14** reaches a viscosity of 1000 mPa·s at 140 °C, which makes it suitable for filament winding, RTM (Resin Transfer Molding) and vacuum infusion.^[^ ^64^ ^]^

The DSC study (Figure 1) of the monomer **11** demonstrated significantly different thermal behavior compared to monomers **12**‐**14**. Specifically, monomer **11** exhibits a distinct melting point, indicative of a crystalline structure, whereas monomers **12**–**14** display only glass transition temperatures (*T*_g_), characteristic of amorphous materials. This difference can be attributed to the fact that all atoms in the backbone are in the sp^2^‐hybridized state, resulting in an extended, conjugated π‐system across the entire molecule, which restricts conformational mobility. This enables molecules to pack more efficiently into a crystalline structure. This enables the molecules to pack more efficiently into a crystalline structure, providing additional order and leading to an increased melting temperature of monomer **11**. In contrast, in monomers **12**–**14**, the conjugation is interrupted by carbon bridges in the BFA and/or Ty fragments (sp^3^ carbons), which enhances molecular mobility and reduces packing density. As a result, these monomers tend to form amorphous states with lower glass transition temperatures.

For each monomer **11**–**14** (Table 1), the DSC curve also exhibited a broad exothermic peak above 280 °C, which could indicate either thermal decomposition or self‐curing of the phthalonitrile monomers via the imine bond. The literature reports^[^ ^38^, ^42^ ^]^ describe self‐curing of phthalonitriles with imine linkages, with curing initiation occurring at temperatures above 275 °C, based solely on DSC data. Regarding present monomers, self‐curing of **14** attempts at 180 °C, a typical temperature for the initial stage of phthalonitrile resin processing, did not result in the formation of an insoluble network. The resin remained soluble, indicating insufficient crosslinking. Heating to 350 °C resulted in the formation of an insoluble material, indicating that self‐curing had occurred, but accompanied by a significant mass loss of 15% during the curing. Thermogravimetric analysis (TGA) of the neat monomers **11**–**14** confirmed significant mass loss (ranging from 10 to 50%) between 200 and 300 °C (Figure 1). This decomposition occurs at the same temperature range as the broad exothermic peak observed in the DSC, which is commonly attributed to self‐curing of phthalonitriles with imine linkages in the literature.^[^ ^38^, ^42^ ^]^ Thus, the self‐curing mechanism and the origin of the substantial mass loss require further investigation. It should be noted that self‐curing of the imine‐containing monomers is challenging for applications due to a high mass loss and requires using special heat‐resistant auxiliary materials for molding. For these reasons for the further investigation, the resins containing curing initiator were taken.

### 2.3 Curing Behavior

**Figure 3:**
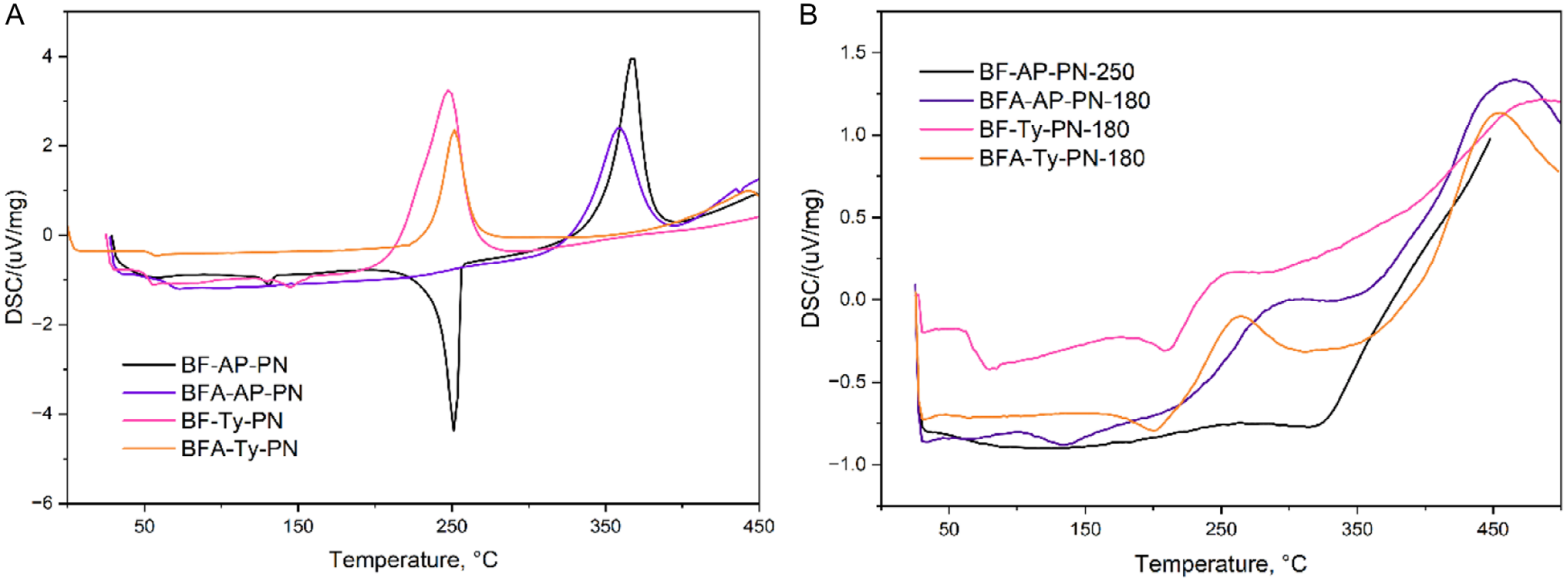
DSC of A) melted and B) cured at 180 °C (250 °C for BF‐AP‐PN) resins with 10 mol% of APN.

Each monomer of **12**–**14** was mixed with 10 mol% APN as a curing agent using a mortar and pestle, then melted and degassed under vacuum at 130 °C. As observed in the DSC curves of the uncured resins (**Figure** **3**A), the addition of the curing agent decreases the onset temperature of the curing exothermal peaks compared to the neat monomers (Figure 1). The absence of a distinct melting peak of APN around 135 °C^[^ ^57^ ^]^ indicates the formation of a homogeneous and amorphous mixture for BFA‐AP‐PN and BFA‐Ty‐PN mixtures, with a glass transition temperature around 50 °C. BF‐AP‐PN and BF‐Ty‐PN do not form homogeneous mixtures, as evidenced by the persistent melting peak of APN (Figure 3A).

Due to the high melting point of **11** (≈250 °C), thermal processing below the curing onset was not feasible, preventing conventional melting and degassing procedures. Consequently, after the addition of the curing agent (10 mol% APN), the mixture was sealed by brief thermal treatment at 250 °C for 5 min. A residual melting peak corresponding to APN is still detectable on the DSC curve (Figure 3A). Additionally, the position of the curing exotherm remains unchanged relative to the neat monomer without the curing agent, while its area increases significantly (from 315 to 460 μV mg^−1^). These findings suggest that a fully homogeneous melt is not achieved. However, the presence of the curing agent facilitates a more rapid and efficient curing process.

As previously noted, phthalonitriles typically cure in two stages. From here and further, cured resins will be named as follows: Name_of_the_monomer – Temperature_of_the_last_curing_stage, for example, BF–AP‐PN‐250 corresponds to a thermoset obtained from the monomer **11** mixed with 10% of APN and cured at 250 °C. The first stage occurs at 180–200 °C, primarily forming polyisoindoline structures.^[^ ^61^ ^]^ This is followed by a postcuring stage at 280–350 °C, during which structural reorganization takes place, leading to the formation of phthalocyanines and triazines.^[^ ^65^ ^]^

Due to the high melting point of **11** (≈250 °C), the first stage of curing for BF–AP–PN was conducted at 250 °C for 6 h. After the first stage, a mass loss of 2.7% was recorded, which may be attributed to ammonia release resulting from the reaction of the amino group in APN with polyisoindoline.^[^ ^66^ ^]^ The release of ammonia during the first stage was confirmed using wet litmus paper (Video S1, Supporting Information). The same behavior and 1–2% of mass loss for the rest of resins during first stage of curing were detected.

According to the DSC curves recorded before and after curing at 180 °C of BFA‐AP‐PN, BF–AP–PN, and BFA‐Ty‐PN, a clear change in the shape of the curing peaks is observed, along with a significant reduction in their area (Figure 3B), indicating that the curing reactions have already progressed to a considerable extent at this stage. Solid thermosets were obtained after the first stage of the curing and were used for characterization and further postcuring.

Since the initial curing of the BF‐AP‐PN was carried out at 250 °C, whereas the other three systems were cured at 180 °C, a postcuring step at 250 °C (4 h) was applied to them to enable a consistent comparison of their thermal properties. Additionally, all the samples were subjected to a postcuring step at 350 °C to ensure full cross‐linking and to evaluate their thermal performance.

**Figure 4:**
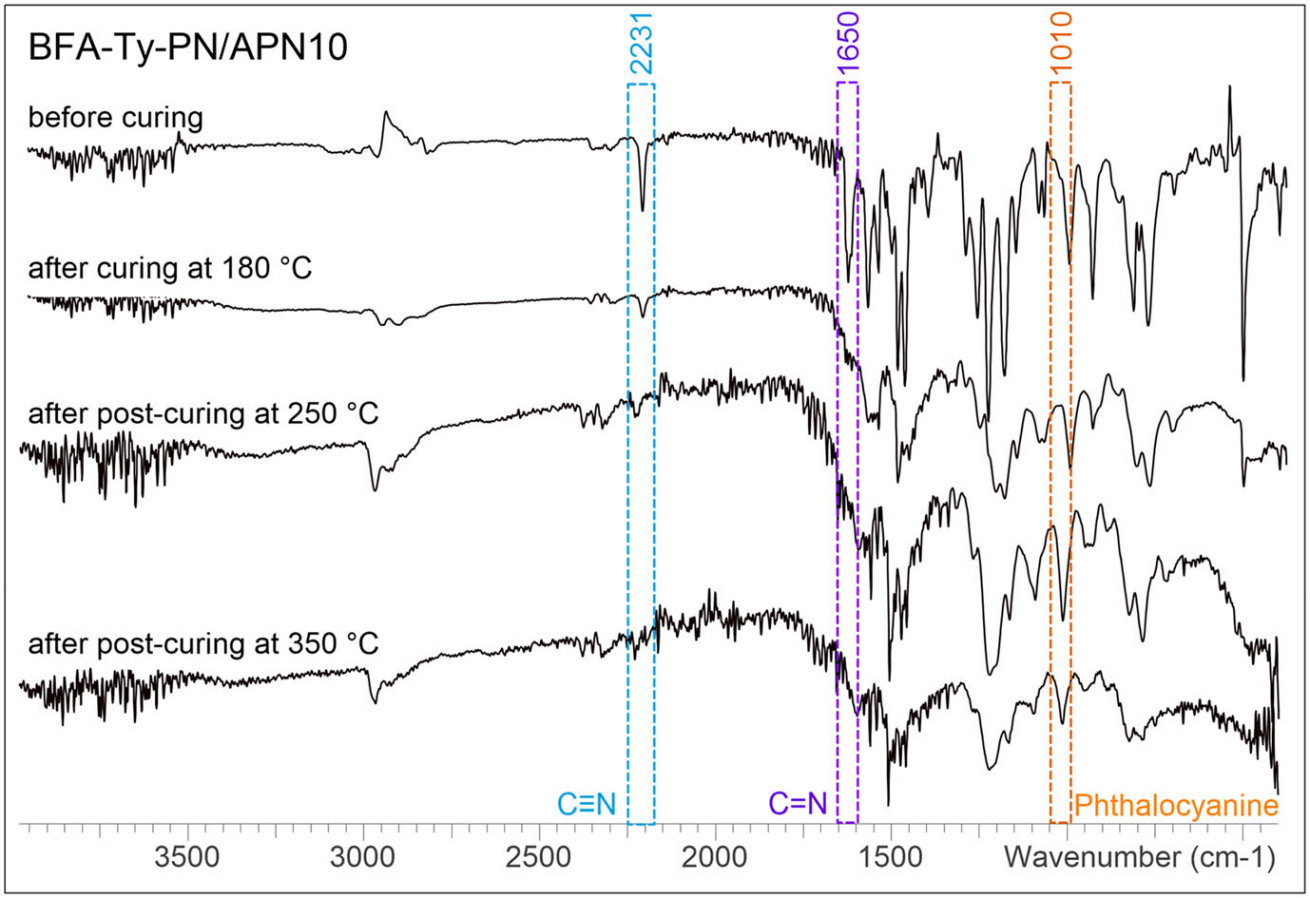
FT‐IR spectra of uncured and cured at different temperatures BFA‐Ty‐PN/APN10 resin.

To observe chemical transformations at the different stages of the curing process, FT‐IR analysis was performed (**Figure** **4**). The result indicates a decrease in the intensity of the absorption band corresponding to the nitrile (–CN) groups (2231 cm^−1^)^[^ ^67^ ^]^ with the increase in curing temperature. Absorbance peaks in the triazine vibration region (1525, 1350 cm^−1^)^[^ ^67^ ^]^ are not distinguishable. Subsequent postcuring at 250 and 350 °C further indicates enhanced absorbance associated with phthalocyanine structures (1010 cm^−1^)^[^ ^67^ ^]^. Before curing, the peaks in imine vibration region (1630 cm^−1^)^[^ ^38^, ^68^ ^]^ clearly indicate the presence of imines in the structure. After curing, due to the formation of isoindolines (1730–1650 cm^−1^)^[^ ^67^ ^]^ and the increased absorbance in this region, the imine bond becomes indistinguishable.

### 2.4 Thermal Properties of the Thermosets

**Figure 5:**
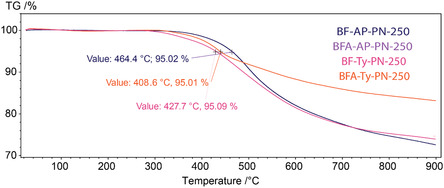
TGA curves of resins postcured at 250 °C.

**Figure 6:**
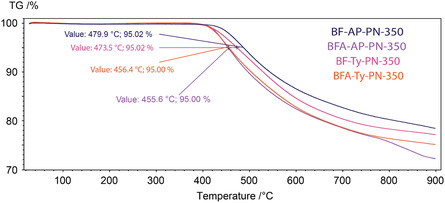
TGA curves of resins postcured at 350 °C.

TGA was performed for the cured resins after the first curing stage and after postcuring (**Figure** **5** and **6**). The temperatures at which 5% mass loss (*T*_5%_) occurs for all resins cured at 250 °C exceed 400 °C. Curing at 350 °C for an additional 4 h provides only a modest improvement in thermal stability, increasing T_5%_ by ≈10 to 20 °C for each resin (**Table** **2**). Although a significant improvement in thermal stability was observed in the shape of the degradation curves—for the BF‐AP‐PN‐250 after the first curing stage at 250 °C (Figure 5), decomposition gradually begins at ≈350 °C, whereas the postcured sample exhibits a sharp mass loss only after 430 °C.

**Table 2:** Characterization of thermal stability of the resins.

Resin [11–14 + 10 mol% APN]	Structure	Bio‐based atom content [mass%]	Highest curing temperature [°C]	Tg [°C]	T5% [°C]	Char yield (900 °C) [%]
BF‐AP‐PN	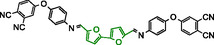	26	250	303	464	74
			350	408	479	76
BFA‐AP‐PN	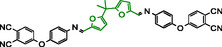	30	250	293	426	72
			350	427	455	73
BF‐Ty‐PN	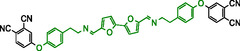	62	250	286	428	75
			350	427	473	78
BFA‐Ty‐PN	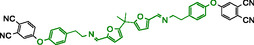	65	250	312	430	74
			350	433	456	76
VTPN^[^ ^38^ ^]^	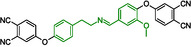	46	320	415	510	76
			360	445	506	77
Resins from Cinnamaldehyde^[^ ^42^ ^]^	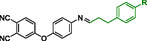	≈35–45	380		524–542	77–80

**Figure 7:**
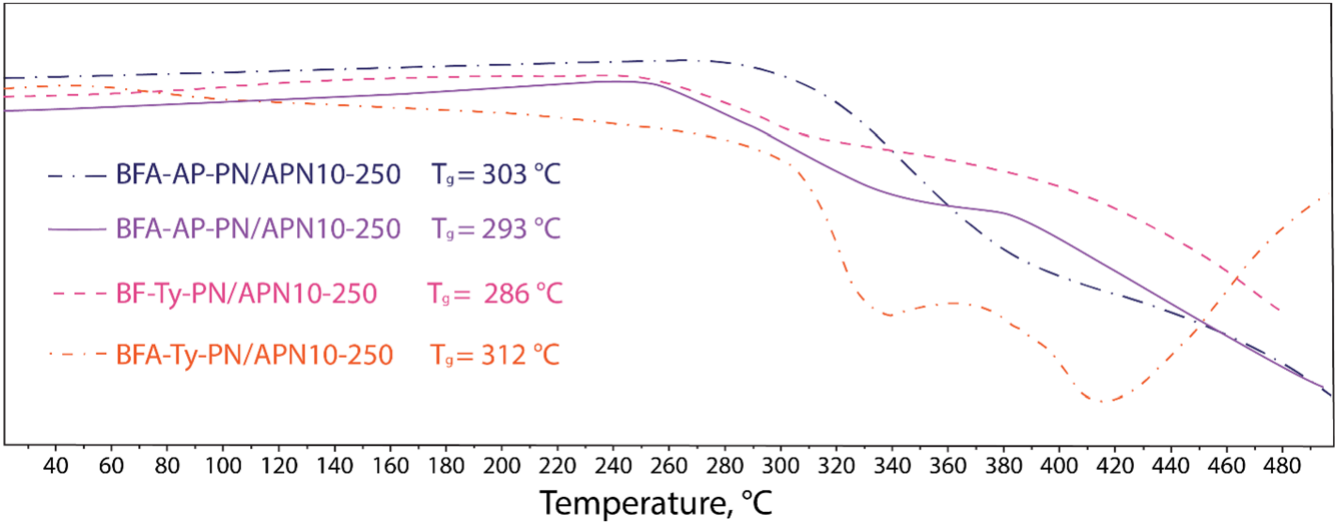
TMA of all resins cured at 250 °C.

**Figure 8:**
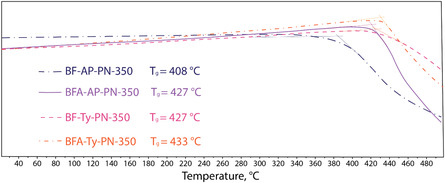
TMA curves of all resins cured at 350 °C.

To determine the glass transition temperature (*T*_g_) of the cured resins, thermomechanical analysis (TMA) was performed (**Figure** **7** and **8**). The TMA results reveal that after a single stage curing process at 250 °C—without the application of a subsequent post‐curing step—the resulting thermoset demonstrates a *T*_g_ of ≈300 °C. For comparison with the lower‐melting monomers **12**–**14**, a two‐step curing process was also applied to them, involving initial curing at 180 °C followed by a second stage at 250 °C. The resulting materials exhibited a glass transition temperature similar to that of BF‐AP‐PN‐250, despite the differences in the curing protocols. This indicates that a substantial degree of crosslinking can be achieved even under relatively mild curing conditions—compared to conventional protocols at 280–350 °C—when an appropriate combination of monomer and curing agent is employed. Therefore, it can be stated that BF‐AP‐PN can still be effectively cured in a single stage, which could be advantageous despite its high melting point. Furthermore, if a postcuring stage at 350 °C is proceeded, the glass transition temperature (*T*_g_) increases to over 400 °C, further enhancing the thermal stability of the cured resin.

According to the TMA analysis (Figure 8), all resins exhibit comparable glass transition temperatures after curing at 350 °C, with *T*_g_ values ranging from 408 to 433 °C. The corresponding decomposition temperatures (*T*_5%_) lie within 455–479 °C, confirming the high thermal stability of the cured polymers. An imine‐based compound reported previously^[^ ^40^ ^]^ demonstrated similar glass transition behavior but achieved slightly higher decomposition temperatures (510–506 °C) when cured at 320 °C and 360 °C (Table 2). Phthalonitrile resins derived from cinnamaldehyde^[^ ^44^ ^]^ displayed a *T*_g_ of 380 °C and *T*_5%_ values in the range of 524–542 °C, with a char yield of 77–80%. Although the decomposition temperatures of the present resins are somewhat lower, comparable char yields (≥74%) are achieved even at lower curing temperatures (350 °C), indicating efficient crosslinking and the formation of thermally stable polymer networks.

### 2.5 Thermal Decomposition Prediction

Despite a slight decrease in decomposition temperatures observed by TGA compared to previously reported imine‐linked phthalonitriles, the operational limits of the presented materials remain promising. As noted in earlier studies, the critical temperature for long‐term operation of phthalonitrile resins in air is limited to 300 °C,^[^ ^35^, ^69^ ^]^ due to rapid thermal oxidation at higher temperatures. Therefore, it can be concluded that the resins presented here retain the high‐performance characteristics typical of phthalonitrile resins and are consistent with the thermal behavior of most bio‐based phthalonitriles.^[^ ^36^ ^]^ TGA data also indicate that resins containing the BFA linker exhibit lower thermal stability than those with the BF linker, suggesting that this structural fragment may limit the thermal resistance of the resulting thermosets. To investigate this hypothesis further, the decomposition behavior was examined using computational methods.

Experimental observations revealed a difference of ≈20 °C in decomposition temperatures between monomers containing the BF and BFA linkers, while the presence of Ty and AP showed no significant effect on thermal stability (Table 2). It's possible that the presence of BF or BFA may influence the stability of the imine bond, potentially affecting the decomposition pathway. Understanding this observation requires an assessment of the bond dissociation energies of the two distinct monomers, **11** and **12**, to identify the most favorable reaction pathway for their decomposition.

**Figure 9:**
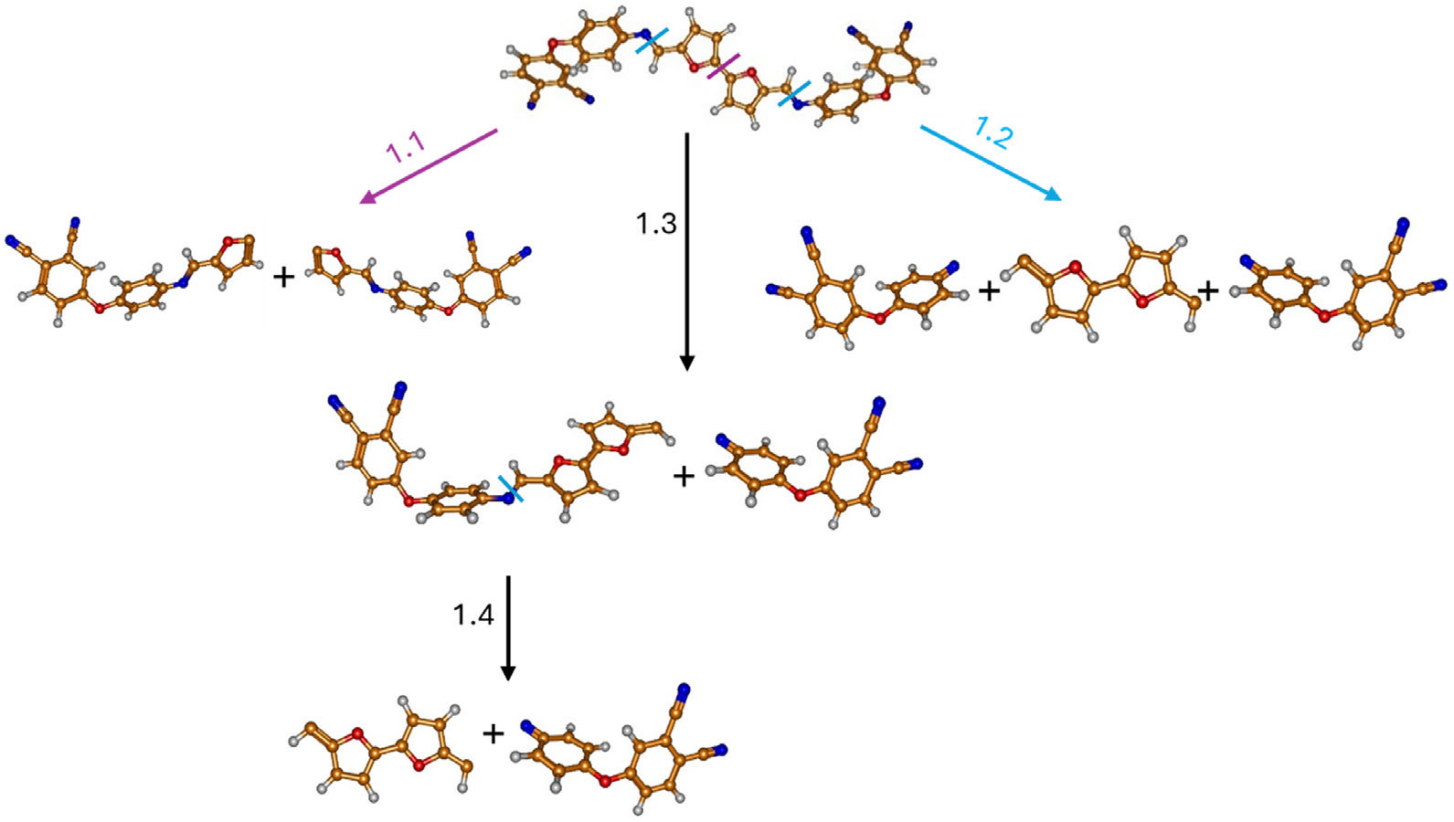
Schematic representation and labeling of the decomposition pathways of **11**.

The decomposition temperatures of **11** and **12** are comparatively lower than that of the previously described molecule containing phthalonitrile rings and ether bonds,^[^ ^13^, ^25^ ^]^ and this observation motivated the investigation of bond dissociation energies of the new fragments were in focus. Table S1, Supporting Information, presents the corresponding calculated enthalpies and Gibbs free energies of the optimized fragments at *T* = 298 K and *T* = 653 K. **Figure** **9** shows a schematic representation of the possible reaction pathways at the two different temperatures for the optimized fragments after breaking the corresponding bond. In **Table** **3**, the enthalpy differences (ΔH) and Gibbs free energy differences (ΔG) for the decomposition reaction pathways at *T* = 298 K and *T* = 653 K are presented.

**Table 3:** The enthalpy differences (ΔH) and Gibbs free energy differences (ΔG) of the decomposition reaction paths of **11** corresponding to the bond cleavage shown in Figure 9.

Reaction path	ΔG [kcal mol^−1^]		ΔH [kcal mol^−1^]	
	*T* = 298 K	*T* = 653 K	*T* = 298 K	*T* = 653 K
1.1.	121.43	104.85	135.52	134.96
1.2.	287.87	256.00	313.94	319.48
1.3.	141.33	125.19	154.52	157.32
1.4.	146.54	130.81	159.41	162.16

As shown in Figure 9 and Table 3, the blue reaction pathway 1.2 corresponds to a combination of two elementary steps from the black reaction mechanism, namely steps 1.3 and 1.4. Consequently, the energy of pathway 1.2 equals the sum of the energies of steps 1.3 and 1.4. It is noteworthy that increasing the temperature from 298 to 653 K leads to only minor changes in the enthalpy differences. In contrast, the Gibbs free energy differences exhibit significantly greater temperature dependence, with differences of up to 20 kcal mol^−1^, due to the T·ΔS contribution. Among all the investigated reaction pathways, the violet pathway 1.1 is thermodynamically the most favorable, despite its comparatively high bond dissociation energy.

**Figure 10:**
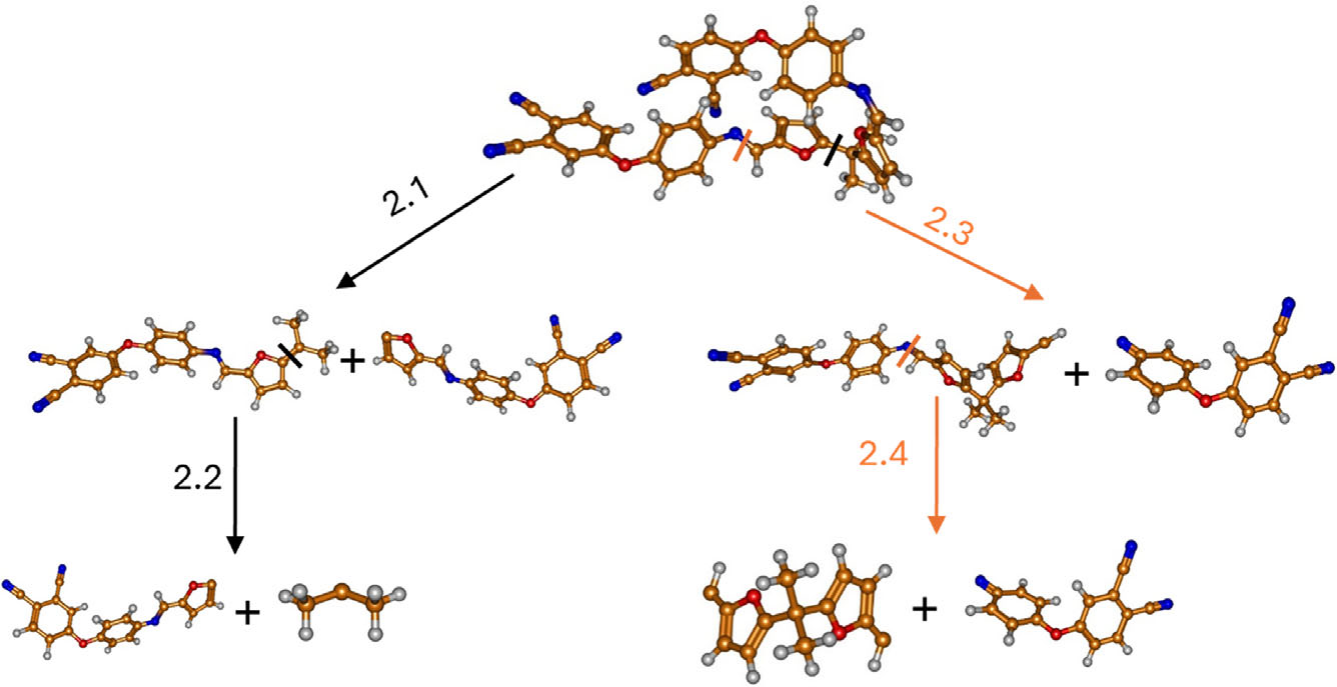
Schematic representation and labeling of the decomposition pathways of **12**.

Table S1, Supporting Information, presents the calculated enthalpies and Gibbs free energies of the optimized fragments of the **12** molecule at *T* = 298 K and *T* = 653 K. **Figure** **10** provides a schematic representation of the possible reaction pathways and **Table** **4** summarizes the corresponding calculated enthalpies and Gibbs free energies for this monomer.

**Table 4:** The enthalpy differences (ΔH) and Gibbs free energy differences (ΔG) of the decomposition reaction paths of **12** corresponding to the bond cleavage shown in Figure 10.

Reaction path	ΔG [kcal mol^−1^]		ΔH [kcal mol^−1^]	
	*T* = 298 K	*T* = 653 K	*T* = 298 K	*T* = 653 K
2.1.	72.53	51.31	90.49	89.98
2.2.	120.89	103.09	135.29	134.97
2.3.	141.58	118.46	160.64	163.45
2.4.	141.66	125.52	154.86	157.66

Among the two proposed reaction pathways for **12** shown in Figure 10, the black pathway, which involves C—C bond cleavage (2.1), exhibits the lowest Gibbs free energy change and is, therefore, expected to occur first among the considered decomposition pathways at elevated temperatures. Both steps of the black reaction path require less energy than step 2.3.

Furthermore, a comparison of the decomposition paths of the two polymers reveals that the energy required to break the C—C bond (2.1) in **12** is almost twice as low as that required for bond cleavage in **11** (1.1). In contrast, the energy of the C—N bond cleavage (1.3 and 2.3) is similar for both monomers and therefore cannot account for the observed differences in decomposition temperature. Additionally, there is a notable difference in the geometry of the two monomers: **11** adopts a more planar structure, while in **12**, there appears to be an interaction—likely π–π stacking—between aromatic rings.

**Figure 11:**
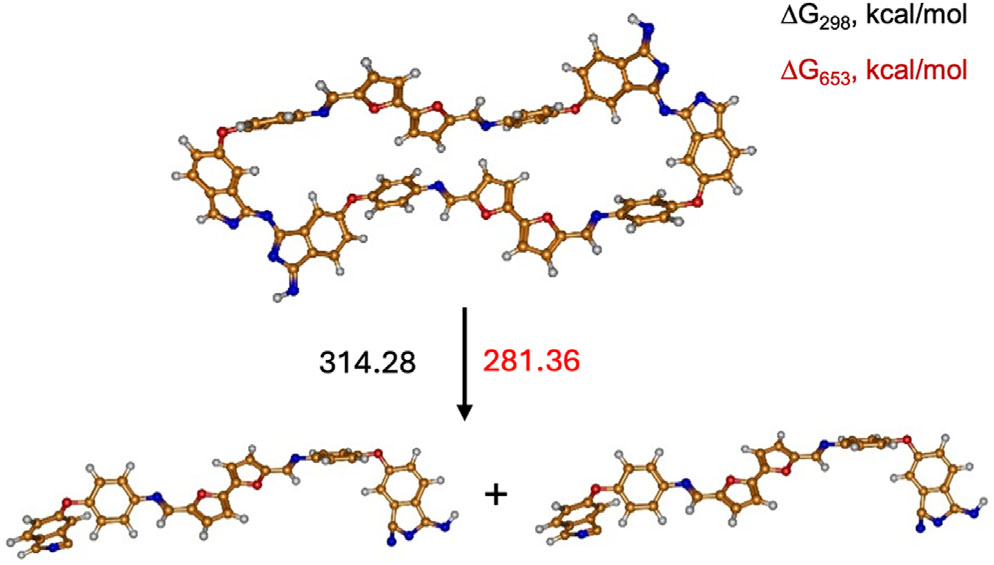
Schematic representation and labeling of the decomposition pathway of BF‐AP‐PN (**11**) dimer into its monomeric units.

**Figure 12:**
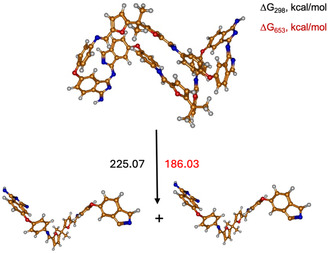
Schematic representation and labeling of the decomposition pathway of BFA‐AP‐PN (**12**) dimer into its monomeric units.

In addition, the decomposition of dimeric model molecules was evaluated to study bond dissociation energies within the thermosetting network. In this case, isoindoline structures were considered as reaction products, as they are the first to form during curing.^[^ ^13^ ^]^ The corresponding structures are represented schematically in **Figure** **11** and **12**.

Based on the calculated ΔG_(298)_ and ΔG_(653)_ values for both considered monomers, it is evident that the C—N bond between the monomeric subunits is the strongest among all intramolecular bonds within the thermoset network. At the same time, a notable difference of ≈100 kcal mol is observed in the decomposition energies of the BFA‐AP‐PN (**12**) and BF‐AP‐PN (**11**) dimers, which may be attributed to the higher flexibility of the BFA linker and the disruption of conjugation compared to the BF‐based monomers. Thus, the results demonstrate that the linker architecture in bis‐phthalonitriles plays a crucial role in the thermal stability of the resulting thermosets, while the cross‐links formed during curing exhibit higher decomposition energies.

## 3 Conclusions

To explore the suitability of a furan‐based chemical platform for incorporation into phthalonitrile resins, four novel phthalonitrile monomers were synthesized using furan‐derived dialdehydes. These monomers (**11**–**14**) were prepared via the formation of imine bonds to connect phthalonitrile‐bearing end groups. The synthesis followed a two‐step procedure, starting from the dialdehydes BF and BFA. In the first step, BF or BFA was reacted with either 4‐aminophenol or tyramine in ethanol, followed by a nucleophilic substitution with 4‐nitrophthalonitrile in DMSO. The use of bio‐based furfural as a starting material for the dialdehydes offers an environmentally friendly approach; however, the dialdehyde synthesis process requires further optimization to align more closely with green chemistry principles.

The processability of the synthesized monomers was evaluated using DSC and rheological analysis. It was found that the monomer **11** exhibited a high melting point (251 °C), whereas the **12**–**14** monomers were amorphous and displayed glass transition temperatures ranging from 50 to 75 °C. Despite the low *T*_g_, the viscosity of monomers **12**–**14** remained high until around 100 °C, getting lower at 170 °C. **14** reaches 1000 mPa s at 140 °C, indicating suitability for vacuum infusion processing. All the monomers were cured with final temperatures of 250 °C and 350 °C demonstrating almost no difference in decomposition temperatures, but difference in *T*_g_. Considering fast oxidation above 300 °C, they can be used at these temperatures to serve as fire protection in batteries, coatings, and adhesives.

Theoretical analysis of the decomposition pathways of monomers **11** and **12** revealed that cleavage of the C—C bond in the furan linkers was the most favorable route in both cases. However, the required energy for this step was nearly twice as low in monomer **12**, explaining its lower experimental decomposition temperature. C—N bond dissociation energies were similar and did not contribute significantly to the observed thermal differences. While enthalpy changes remain nearly constant with temperature, Gibbs free energy differences varied by up to 20 kcal mol^−1^ due to the T·ΔS contribution, highlighting the important contribution of entropy in thermal decomposition at elevated temperatures.

These results demonstrate that the furan platform holds significant promise for the design of novel bio‐based phthalonitrile resins with high‐performance characteristics. As shown, the specific choice of furfural‐derived moieties influences the thermal stability of the resulting thermosets, highlighting the ongoing challenge—and opportunity—of developing new furan‐based architectures. The combination of high‐performance properties and partial bio‐origin of the presented resins contributes to advancing the broader objective of achieving carbon neutrality, as outlined in the European Union's sustainability goals.

## 4 Experimental Section

4.1

4.1.1

##### Materials

Potassium acetate, palladium acetate, Amberlyst 15, and *p*‐toluenesulfonic acid were purchased from Sigma–Aldrich (Merck KGaA, Darmstadt, Germany). All other chemicals were used as received from TCI Deutschland GmbH, if not otherwise stated. All nondried organic solvents (acetone, ethyl acetate, diethyl ether, dichloromethane, tetrahydrofuran) used were reagent grade and distilled prior to use. Dry organic solvents were either obtained from an SPS MBRAUN MB SPS‐800 or as dried solvent from TCI or VWR. Microwave‐assisted organic synthesis was performed using Biotage Initiator+ synthesis microwave using the respective Biotage pressure‐tight microwave reaction vials (0.5–2 mL and 2–5 mL) and caps equipped with septum. All microwave‐assisted reactions were carried out in pressure‐resistant vials using ethanol as the solvent at 150 °C. The microwave reactor automatically adjusted the output power, applying ≈400 W during the heating phase and 100 W to maintain the target temperature. The internal pressure during the reactions reached up to 7 bar.

##### Methods

Proton nuclear magnetic resonance (^1^H NMR), ^13^C NMR, correlation spectroscopy (COSY), and heteronuclear single‐quantum correlation spectroscopy (HSQC) spectra were acquired by using a Bruker AC 300 (300 MHz) spectrometer at 298 K. The deuterium signal of the DMSO–d6 was used as internal reference. Fourier‐transform infrared (FT‐IR) spectra were recorded from 400 to 4000 cm^−1^ using an IR‐Affinity‐1 CE system (Shimadzu, Kyoto, Japan) which was equipped with a quest ATR diamond extended range X—single reflection‐ATR accessory with a diamond crystal. ESI‐Q‐TOF MS measurements were performed with a micrOTOF Q‐II (Bruker Daltonics) mass spectrometer equipped with an automatic syringe pump from KD Scientific for sample injection. The solvent was acetonitrile. Elemental analysis (EA) was performed with a Euro EA3000 from Eurovector.

TGA was carried out under nitrogen or air atmosphere using a Netzsch TG209 F1 Libra (Selb, Germany). The measurements were performed from 25 to 900 °C with a heating rate of 10 °C min^−1^ using Al_2_O_3_ pans. DSC measurements were performed on a Netzsch DSC 204 F1 Phoenix from −50 to 450 °C under N_2_ atmosphere with a heating rate of 10 °C min^−1^ using an aluminum pan with a lid.

TMA was performed using a Method Toledo LN/600 analyzer to determine the glass transition temperature of cured resins and detect. A test specimen measuring ≈5 mm × 1.5 mm is placed flat side up on the bending test device. The support width is 10 mm, and the contact force is adjusted to the material being tested and is typically in the range of 0.02–0.1 N. The test specimen assembly is heated from room temperature to 500 °C at a heating rate of 10 K min^−1^. The change in length is determined as a function of temperature. The glass transition is evaluated as the center value of the envelope curve. A rheometer MCR302e (Anton Paar, Austria) was used to measure shear rheological behavior. Temperature sweeps were conducted under steady‐state shear conditions at a shear rate of 30 s^−1^ from 120 to 190 °C at a heating rate of 10 °C min^−1^.

Detailed synthetic procedures for all compounds as well as their characterization data are provided in the Supplementary Information.

##### Computational Details

In order to investigate the decomposition paths of the different molecules computational calculations were performed. To determine the most favorable structures, a conformational search was carried out for both monomer structures using the CREST program package.^[^ ^70^ ^]^ The most stable conformer at xtb level for each structure was then chosen for further decomposition analysis using density functional theory (DFT). All DFT calculations were performed using the Gaussian program package^[^ ^71^ ^]^ employing the level of theory B3LYP‐D3(BJ)/6‐311G(d,p).^[^ ^72^, ^73^, ^74^, ^75^ ^]^ For the calculation of the enthalpies and Gibbs free energies of the corresponding bonds, the following Equation (1) and (2) were used.^[^ ^76^, ^77^ ^]^

(1) $$\Delta H=\sum H_{\text{fragment}}^{\text{opt}}-H_{\text{initial}}^{\text{opt}}$$

(2) $$\Delta G=\sum G_{\text{fragment}}^{\text{opt}}-G_{\text{initial}}^{\text{opt}}$$

It should be noted that the number of atoms must be the same in both the initial structure and the fragments. Furthermore, vibrational entropy corrections have to be considered in the analysis. In addition to the calculation at *T* = 298 K, the temperature of 653 K was selected to reflect the experimental conditions, as changes at this temperature in the experiment are more pronounced (decomposition occurs), thereby enabling a more direct comparison with the experimental results.

Further computational details are provided in the Supplementary Information.

## Conflict of Interest

The authors declare no conflict of interest.

## Supporting information

Supplementary Material

## Data Availability

The data that support the findings of this study are available in the supplementary material of this article.
