# Supplementary material for: High‐Performance Phthalonitrile Resins Partially Derived from a Furan Bio‐Based Chemical Platform
Source: ChemSusChem. 2025 Oct 31;18(24):e202501854. doi: 10.1002/cssc.202501854 (PMC12703453; doi:10.1002/cssc.202501854)
Supplement: Supplementary file 1 — Supplementary Material [file CSSC-18-e202501854-s001.zip › cssc70251-sup-0001-SuppData-S1.pdf]

Supplementary Information

High-Performance Phthalonitrile Resins Partially Derived from a Furan Bio-based Chemical Platform

Daria I. Poliakova[a], Sergey S. Nechausov[b], Daria S. Stepaniuk[b, c], Doreen Mollenhauer[b, c, d], Ulrich S. Schubert[a, b]*, Boris A. Bulgakov[a]*

[a] Daria Poliakova, Dr. Boris Bulgakov, Prof. Dr. Ulrich S. Schubert
Laboratory of Organic Chemistry and Macromolecular Chemistry (IOMC)
Friedrich Schiller University Jena
Humboldtstr. 10, 07743 Jena, Germany
E-mails: boris.bulgakov@uni-jena.de, daria.poliakova@uni-jena.de

[b] Dr. Sergey Nechausov, Dr. Daria Stepaniuk, Prof. Dr. Doreen Mollenhauer, Prof. Dr. Ulrich S. Schubert
HIPOLE Jena (Helmholtz Institute for Polymers in Energy Applications Jena),
Lessingstrasse 12-14, 07743 Jena, Germany

[c] Dr. Daria S. Stepaniuk, Prof. Dr. Doreen Mollenhauer
Institute for Technical and Environmental Chemistry (ITUC), Friedrich Schiller University Jena, Philosophenweg 7a, 07743 Jena, Germany

[d] Prof. Dr. Doreen Mollenhauer, Prof. Dr. Ulrich S. Schubert
Helmholtz-Zentrum Berlin für Materialien und Energie GmbH (HZB), Hahn-Meitner-Platz 1, 14109 Berlin, Germany

Table of contents

Experimental part. 4

Synthesis procedures 4

Synthesis of 4-(4-aminophenoxy)-phthalonitrile (APN, 1). 4

Synthesis of 4-(4-(2-aminoethyl)phenoxy)phthalonitrile TyPN (15) [2]. 4

Synthesis of 2-furyl-1,3-dioxolane (2). 4

Synthesis of [2,2`-bifuran]-5,5`-dicarbalgehyde (BF, 3) [4]. 5

Synthesis of 2-furyl-1,3-dithiolane (4) 5

Synthesis of 5,5’-(propane-2,2’-diyl)bis[2-(1,3-dithiolan-2-yl)furan] (5) [5]. 5

Synthesis of 5,5’-(propane-2,2’-diyl)difuran-2-carbaldehyde (BFA, 6) [5]. 6

Synthesis of Schiff-bases (7-8), general procedure. 6

Synthesis of Schiff-bases (9-10), general procedure. 7

Nucleophilic substitution between Schiff-bases (7-10) and 4-nitrophthalonitrile. 9

Thermal decomposition calculation. 11

Figure S 1. Comparison of the calculated enthalpies (∆H), and Gibbs free energies (∆G) for the reaction in Figure 3 using B3LYP-D3(BJ) with different basis sets. 12

Table SI1. The enthalpies (H) and Gibbs free energies (G) of the monomers and decomposed optimized fragments corresponding to the bond cleavage calculated at B3LYP-D3(BJ)/6-311G(d,p) level of theory: 1. BF-AP-PN (**11**), 2. BF-APA-PN (**12**), 3. Dimer of BF-AP-PN, 4. Dimer of BFA-AP-PN. 12

Figures 15

Figure S 2. ^{1}H NMR spectrum of BF-AP (**7**). 15

Figure S 3. ^{13}C NMR spectrum of BF-AP (**7**) 16

Figure S 4. COSY and HSQC spectra of BF-AP (**7**). 17

Figure S 5. ESI-MS spectrum of BF-AP (**7**). 18

Figure S 6. TGA and DSC of BF-AP (**7**).19

Figure S 7. ^{1}H NMR spectrum of BFA-AP (**8**). 20

Figure S 8. ^{13}C spectrum of BFA-AP (**8**). 21

Figure S 9. COSY and HSQC spectra of BFA-AP (**8**). 22

Figure S 10. ESI-MS spectrum of BFA-AP (**8**). 23

Figure S 11. TGA and DSC of BFA-AP (**8**). 24

Figure S 12. ^{1}H NMR spectrum of BF-Ty (**9**). 25

Figure S 13. ^{13}C NMR spectrum of BF-Ty (**9**). 25

Figure S 14. COSY and HSQC spectra of BF-Ty (**9**). 26

Figure S 15. ESI-MS spectrum of BF-Ty (**9**). 27

Figure S 16. TGA and DSC of BF-Ty (**9**). 28

Figure S 17. ^{1}H NMR spectrum of BFA-Ty (**10**). 29

Figure S 18. ^{13}C NMR spectrum of BFA-Ty (**10**).30

Figure S 19. COSY and HSQC spectra of BF-Ty (**10**). 31

Figure S 20. ESI-MS spectrum of BFA-Ty (**10**). 32

Figure S 21. TGA and DSC of BFA-Ty (**10**). 33

Figure S 22. ^{1}H NMR spectrum of BF-AP-PN (**11**). 34

Figure S 23. ^{13}C spectrum of BF-AP-PN (**11**). 35

Figure S 24. HSQC spectrum of BF-AP-PN (**11**). 36

Figure S 25. ESI-MS spectrum of BF-AP-PN (**11**). 37

Figure S 26. TGA and DSC of BF-AP-PN (**11**). 38

Figure S 27. ^{1}H NMR spectrum of BFA-AP-PN (**12**). 39

Figure S 28. ^{13}C NMR spectrum of BFA-AP-PN (**12**). 39

Figure S 29. COSY and HSQC spectrum of BFA-AP-PN (**12**). 40

Figure S 30. ESI-MS spectrum of BFA-AP-PN (**12**). 41

Figure S 31. TGA and DSC of BFA-AP-PN (**12**).42

Figure S 32. ^{1}H NMR spectrum of BF-Ty-PN (**13**). 43

Figure S 33. ^{13}C NMR spectrum of BF-Ty-PN (**13**). 44

Figure S 34. COSY and HSQC spectra of BF-Ty-PN (**13**). 45

Figure S 35. ESI-MS spectrum of BF-Ty-PN (**13**). 46

Figure S 36. TGA and DSC of BF-Ty-PN (**13**). 47

Figure S 37. ^{1}H NMR spectrum of BFA-Ty-PN (**14**). 48

Figure S 38. ^{13}C NMR spectrum of BFA-Ty-PN (**14**). 49

Figure S 39. COSY and HSQC spectra of BFA-Ty-PN (**14**). 50

Figure S 40. ESI-MS spectrum of BFA-Ty-PN (**14**). 51

Figure S 41. TGA and DSC of BFA-Ty-PN (**14**). 52

Figure S 42. ^{1}H and ^{13}C NMR spectra of TyPN (**15**). 53

Figure S 43. ^{1}H NMR spectra of reactions between APPN and BF in DMSO (upper) and in ethanol (lower). 54

Figure S 44. ^{1}H NMR spectrum of reactions between BFA and BFA in ethanol. 55

Figure S 45. DSC (solid) and TGA (dashed) of the monomers **7**-**10**. 56

Figure S 46. COSY and HSQC spectra of BFA-Ty-PN (**14**). 57

Figure S 47. TGA and DSC of Ty-PN (**15**). 58

Figure S 48. TyPN (**15**) after recrystallisation. 59

References 60

Experimental part.

Synthesis procedures

Synthesis of 4-(4-aminophenoxy)-phthalonitrile (APN, 1).

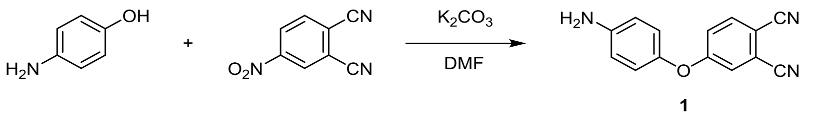

Scheme S 1. Schematic representation of 4 aminophenoxyphthalonitrile synthesis.

Synthesis of 4-(4-aminophenoxy)-phthalonitrile (APN, **1**). **1** was synthesized according to the procedure described in reference[1] from 4-aminophenol and 4-nitrophthalonitrile with 97% yield.

^{1}H NMR (300 MHz, DMSO-d6) *δ* ppm: 5.12 - 5.31 (m, 2H), 6.58 - 6.71 (m, 2H), 6.78 - 6.94 (m, 2H), 7.20 - 7.32 (m, 1H), 7.58 - 7.67 (m, 1H), 7.99 - 8.08 (m, 1H).

^{13}C NMR (151 MHz, DMSO-d6): *δ* ppm: 163.11, 147.43, 143.45, 136.57, 121.85, 121.78, 121.12, 116.90, 116.47, 115.93, 115.42, 107.37.

Synthesis of 4-(4-(2-aminoethyl)phenoxy)phthalonitrile TyPN (15) [2].

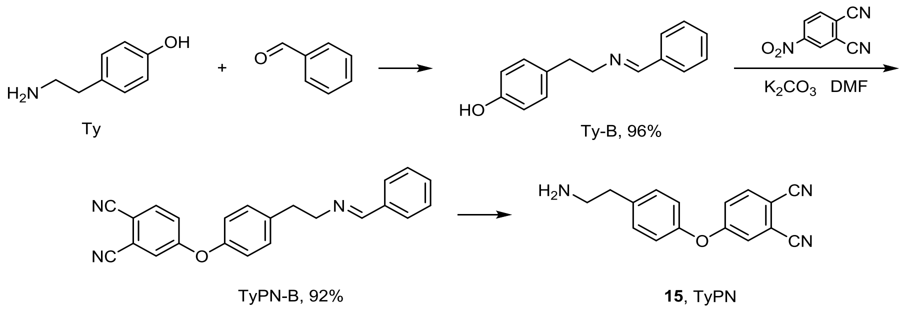

Scheme S 2. Schematic representation of the synthesis procedure of TyPN.

Synthesis of 4-(4-(2-aminoethyl)phenoxy)phthalonitrile (TyPN, **15**). **15** was synthesized according to the procedure described in reference[2] from tyramine and 4-nitrophthalonitrile via stages of protection and deprotection of amino group with 78% yield on the third stage.

^{1}H NMR (400 MHz, $CDCl_3$) *δ* ppm: 7.72 (d, J = 8.6 Hz, 1H), 7.35–7.19 (m, 5H), 7.06–6.98 (m, 2H), 3.01 (t, J = 6.9 Hz, 2H), 2.80 (t, J = 6.9 Hz, 2H), 1.34 (s, 2H).

^{13}C NMR (101 MHz, $CDCl_3$) *δ* ppm: 124.38, 114.22, 100.65, 97.79, 93.37, 83.77, 83.72, 83.04, 79.94, 77.86, 77.43, 71.00, 39.84, 39.52, 39.20, 5.88, 1.79.

Synthesis of 2-furyl-1,3-dioxolane (2).

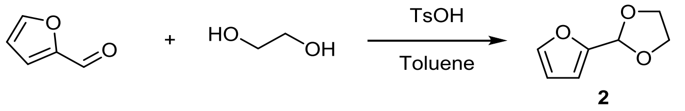

Scheme S 3. Schematic representation of furfural`s aldehyde group protection with ethylene glycol.

Synthesis of 2-furyl-1,3-dioxolane was performed according to the reported procedure[3] from furfural and ethylene glycol with 60% yield resulting with a light-yellow liquid.

^{1}H NMR (300 MHz, DMSO-d6) *δ* ppm: 3.87 - 4.08 (m, 4H), 5.88 (s, 1H), 6.43 - 6.48 (m, 1H), 6.51 - 6.55 (m, 1H), 7.65 - 7.70 (m, 1H).

^{13}C NMR (75 MHz, DMSO-d6) *δ* ppm: 65.00, 97.21, 109.30, 110.73, 143.87, 151.56.

Synthesis of [2,2`-bifuran]-5,5`-dicarbalgehyde (BF, 3) [4].

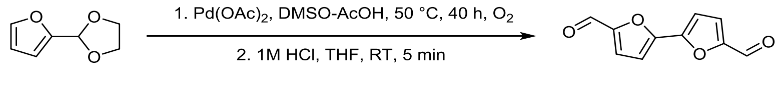

2 **3**

Scheme S 4. Schematic representation of [2,2`-bifuran]-5,5`-dicarbalgehyde synthesis.

[2,2`-Bifuran]-5,5`-dicarbalgehyde (**3**) was synthesized according to reported procedure[4] from palladium acetate (775 mg, 3.32 mmol), DMSO (64 mL), acetic acid (16 mL) and 2-(2-furyl)-1,3-dioxolane (**2**) (4.6 g, 33.2 mmol) resulting with dark yellow solid (1.418 g, 45%).

^{1}H NMR (300 MHz, DMSO-d6) *δ* ppm: 7.30 - 7.40 (m, 2H), 7.68 - 7.77 (m, 2H), 9.62 - 9.72 (m, 2H).

^{13}C NMR (75 MHz, DMSO-d_6) *δ* ppm: 112.36, 125.11, 148.54, 152.87, 178.86.

Synthesis of 2-furyl-1,3-dithiolane (4)

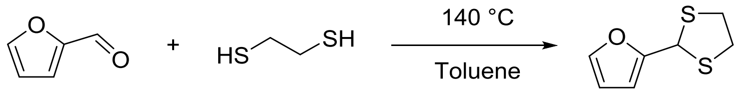

4

Scheme S 5. Schematic representation of 2-furyl-1,3-dithiolane (**4**) synthesis.

2-Furyl-1,3-dithiolane (**4**) synthesis was performed according to the reported procedure[5] from furfural (459 mmol) and ethane-1,2-dithiol (918 mmol) resulting with dark red liquid (yield 98%).

^{1}H NMR (300 MHz, DMSO-d6) *δ* ppm: 3.25 - 3.48 (m, 4H), 5.79 - 5.89 (m, 1H), 6.29 - 6.40 (m, 2H), 7.60 (dd, J=1.83, 0.91Hz, 1H),.

^{13}C NMR (75 MHz, DMSO-d_6) *δ* ppm: 39.20, 46.99, 107.28, 110.90, 143.34, 154.89.

Synthesis of 5,5’-(propane-2,2’-diyl)bis[2-(1,3-dithiolan-2-yl)furan] (5) [5].

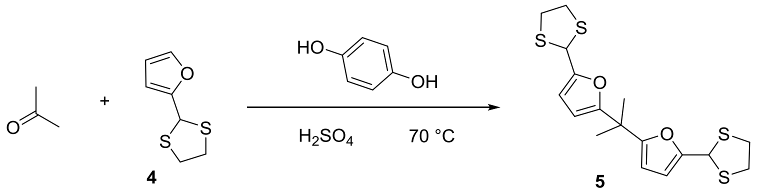

Scheme S 6. Schematic representation of 5,5’-(propane-2,2’-diyl)bis[2-(1,3-dithiolan-2-yl)furan] (**5**) synthesis.

5,5’-(Propane-2,2’-diyl)bis[2-(1,3-dithiolan-2-yl)furan] (**5**) synthesis was performed according to the reported procedure[5] from acetone (25.3 g, 435 mmol) and 2-(1,3-dithiolan-2-yl)furan (15.0 g, 87.1 mmol) resulting with light pink powder with a yield of 60%.

^{1}H NMR (300 MHz, DMSO-d6) *δ* ppm: 1.47 - 1.58 (m, 6H), 3.24 - 3.43 (m, 8H), 5.70 - 5.78 (m, 2H), 5.94 - 6.01 (m, 2H), 6.17 - 6.24 (m, 2H).

^{13}C NMR (75 MHz, DMSO-d_6) *δ* ppm: 26.53, 37.49, 39.07, 47.13, 105.40, 107.47, 153.70, 159.38.

Synthesis of 5,5’-(propane-2,2’-diyl)difuran-2-carbaldehyde (BFA, 6) [5]**.**

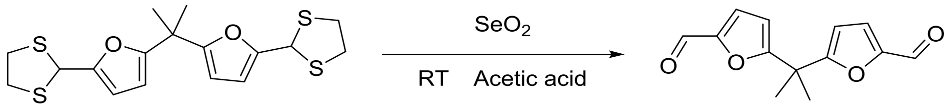

5 **6**

Scheme S 7. Schematic representation of 5,5’-(propane-2,2’-diyl)difuran-2-carbaldehyde synthesis.

5,5’-(Propane-2,2’-diyl)difuran-2-carbaldehyde (BFA, **6**) synthesis was performed according to the reported procedure[5] from 5,5’-(propane-2,2’-diyl)bis[2-(1,3-dithiolan-2-yl)furan] (**5**) (29.3 mmol) and selenium dioxide (60 mmol) resulting with light brown powder with total yield of 68%.

^{1}H NMR (300 MHz, DMSO-d6) *δ* ppm: 1.65 - 1.74 (m, 6H), 6.55 - 6.72 (m, 2H), 7.45 - 7.60 (m, 2H), 9.42 - 9.58 (m, 2H).

^{13}C NMR (75 MHz, DMSO-d_6) *δ* ppm: 25.90, 38.30, 109.18, 124.91, 152.20, 164.81, 178.39.

Synthesis of Schiff-bases (7-8), general procedure.

Aromatic amine (4-aminophenol) (10.0 mmol) and dialdehyde (BF (**3**) or BFA (**6**)) (5.00 mmol) were each dissolved in ethanol (15 mL) mixed in a microwave vial and heated to 150 °C for 1 hour. The mixture was left to precipitate in a refrigerator at 1 to 2 °C for 2 hours. The resulting precipitate was collected by filtration, washed with cold ethanol and diethyl ether, and then dried in an oven at 80 °C for 5 hours.

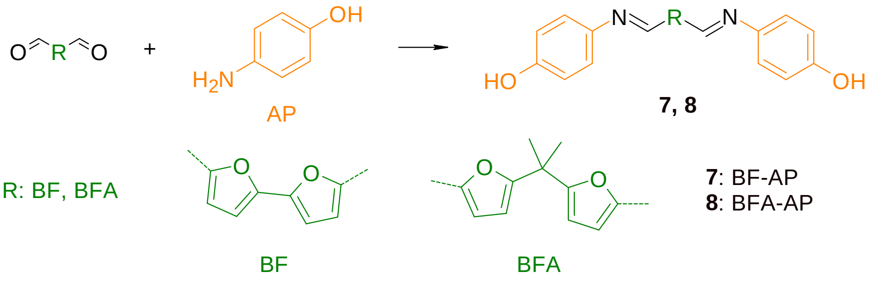

Scheme S 8. Schematic representation of the general scheme for the synthesis of Schiff-bases from dialdehyde and 4-aminophenol.

4,4'-(((1E,1'E)-[2,2'-Bifuran]-5,5'-diylbis(methaneylylidene))bis(azaneylylidene))diphenol, (BF-AP, 7) (**Error! Reference source not found.**).

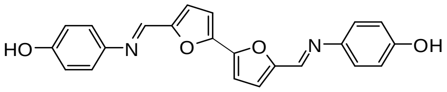

7 BF-AP

Scheme S 9. Schematic representation of the chemical structure of BF-AP (**7**).

BF-AP (**7**) was synthesized according to the procedure described above, bisfurfural (BF, **3**) and 4-aminophenol (AP) were taken as starting materials. Light yellow powder was obtained; yield is 85%. The melting point is 295 °C (DSC and TGA: Figure S5).

^{1}H NMR (300 MHz, DMSO-d6) *δ* ppm: 6.76 - 6.85 (m, 4H), 7.09 - 7.15 (m, 2H), 7.19 - 7.31 (m, 6H), 8.46 - 8.51 (m, 2H) (Figure S1).

^{13}C NMR (75 MHz, DMSO-d6) *δ* ppm: 110.62, 116.26, 118.50, 123.06, 142.70, 144.82, 147.02, 152.90, 157.05 (Figure S2).

MS (ESI, positive mode): *m/z* 373 $[M+H]^+$, M = 472 (Figure S4).

Elemental analysis calculated for $C_{26}H_{24}N_2O_4$: C 72.8%, H 5.65%, N 6.54%. Found: C 72.30%, H 5.65%, N 6.54%.

4,4'-(((1E,1'E)-(Propane-2,2-diylbis(furan-5,2-diyl))bis(methaneylylidene))bis(azaneylylidene))diphenol (BFA-AP, 8), (**Error! Reference source not found.**).

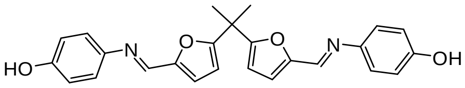

8 BFA-AP

Scheme S 10. Schematic representation of the chemical structure of BFA-AP (**8**).

BFA-AP (**8**) was synthesized according to the procedure described above, 5,5'-(propane-2,2'-diyl)difuran-2-carbaldehyde (BFA, **6**) and 4-aminophenol (AP) were taken as starting materials. White powder was obtained; yield is 85%. The melting point is 281 °C (DSC and TGA: Figure S10).

^{1}H NMR (300 MHz, DMSO-d6) *δ* ppm: 1.65 - 1.78 (m, 6H), 6.44 - 6.53 (m, 2H), 6.71 - 6.82 (m, 4H), 6.98 - 7.05 (m, 2H), 7.08 - 7.18 (m, 4H), 8.28 - 8.35 (m, 2H), 9.44 - 9.52 (m, 2H) (Figure S6).

^{13}C NMR (75 MHz, DMSO-d6) *δ* ppm: 26.44, 37.98, 108.29, 116.14, 117.33, 122.78, 143.00, 145.68, 151.90, 156.63, 162.23 (Figure S7).

MS (ESI, positive mode): *m/z* 415 $[M+H]^+$, 437 $[M+Na]^+$, M = 414. (Figure S9)

Elemental analysis calculated for $C_{25}H_{22}N_2O_4$: C 72.45%, H 5.35%, N 6.76%. Found: C 70.83%, H 5.69%, N 5.71%.

Synthesis of Schiff-bases (9-10), general procedure.

Tyramine (Ty) (10.0 mmol) and dialdehyde (BF (**3**) or BFA (**6**)) (5.00 mmol) were each dissolved in ethanol (15 mL) mixed in a microwave vial and heated to 150 °C for 1 hour. The mixture was left to precipitate in a refrigerator at 1 to 2 °C for 2 hours. The resulting precipitate was collected by filtration, washed with cold ethanol and diethyl ether, and then dried in an oven at 80 °C for 5 hours.

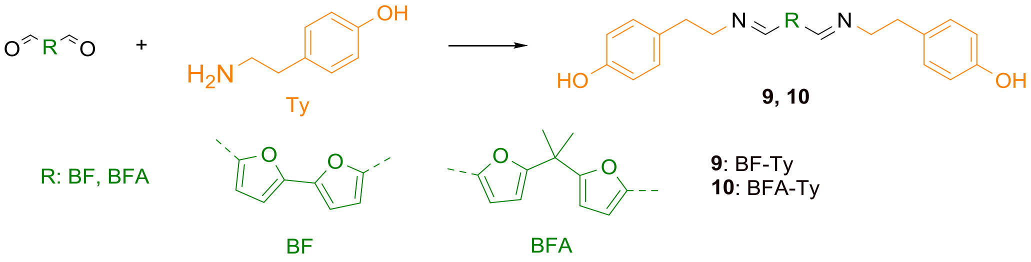

Scheme S 11. Schematic representation of the general scheme for synthesis of Schiff-bases from dialdehyde and tyramine.

4,4'-((((1E,1'E)-[2,2'-Bifuran]-5,5'-diylbis(methaneylylidene))bis(azaneylylidene))bis(ethane-2,1-diyl))diphenol, (BF-Ty, 9), (Error! Reference source not found.**).**

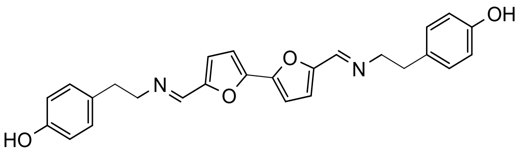

9 BF-Ty

Scheme S 12. Schematic representation of the chemical structure of BF-Ty (**9**).

BF-Ty (**9**) was synthesized according to the procedure described above, bisfurfural (BF, **3**) and tyramine (Ty) were taken as starting materials. Light yellow powder was obtained; yield is 71%. (DSC and TGA: Figure S15).

^{1}H NMR (300 MHz, DMSO-d6) *δ* ppm: 2.91 - 3.09 (m, 4H), 3.75 - 3.91 (m, 4H), 7.02 (dd, J=18.39, 3.54 Hz, 4H), 7.13 (br d, J=8.45 Hz, 4H), 7.29 - 7.47 (m, 6H), 7.70 - 7.81 (m, 2H), 8.03 - 8.18 (m, 4H) (Figure S11).

^{13}C NMR (75 MHz, DMSO-d6) *δ* ppm: 36.44, 63.16, 109.67, 115.50, 116.81, 130.10, 130.20, 146.66, 150.03, 151.82, 155.99 (Figure S12).

MS (ESI, positive mode): m/z 429.00 ($[M + H]^+$), M = 428.00 (Figure S14).

4,4'-((((1E,1'E)-(Propane-2,2-diylbis(furan-5,2-diyl))bis(methaneylylidene))bis(azaneylylidene))bis(ethane-2,1-diyl))diphenol, (BFA-Ty. 10) (Error! Reference source not found.**).**

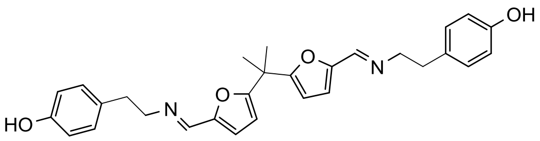

10 BFA-Ty

Scheme S 13. Schematic representation of the chemical structure of BFA-Ty (**10**).

BFA-Ty (**10**) was synthesized according to the procedure described above, 5,5'-(propane-2,2'-diyl)difuran-2-carbaldehyde (BFA, **6**) and Tyramine (Ty) were taken as starting materials. White powder was obtained; yield is 83%. T_{mp} = 223 °C (DSC and TGA: Figure S20).

^{1}H NMR (300 MHz, DMSO-d6) *δ* ppm: 1.58 - 1.70 (m, 6H), 2.68 - 2.83 (m, 4H), 3.57 - 3.72 (m, 4H), 6.29 - 6.38 (m, 2H), 6.60 - 6.72 (m, 4H), 6.76 - 6.85 (m, 2H), 6.96 - 7.09 (m, 4H), 7.95 - 8.06 (m, 2H), 9.07 - 9.22 (m, 2H) (Figure S16).

^{13}C NMR (75 MHz, DMSO-d6) *δ* ppm: 26.37, 36.56, 37.78, 63.08, 107.58, 115.35, 115.46, 130.05, 130.28,150.37, 151.00, 155.97, 161.58 (Figure S17).

MS (ESI, positive mode): *m/z* 471 $[M+H]^+$, M = 470. (Figure S19)

Elemental analysis calculated for $C_{29}H_{30}N_2O_4$: C 74.02%, H 6.43%, N 5.95%. Found: C 73.93%, H 6.51%, N 5.91%.

Nucleophilic substitution between Schiff-bases (7-10) and 4-nitrophthalonitrile.

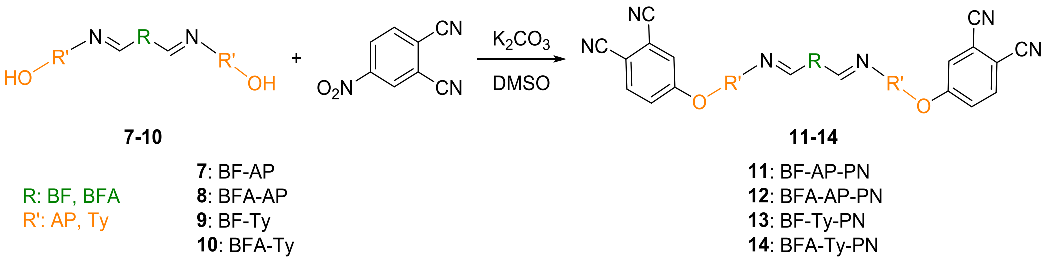

Scheme S 14. Schematic representation of the general synthesis of target monomers (**11-14**).

General synthesis procedure for 10-14. 7-10 (2 mmol), potassium carbonate (6 mmol), and anhydrous dimethyl sulfoxide (DMSO) were added to a 100 mL three-necked round-bottom flask equipped with a reflux condenser and a magnetic stirring bar. The mixture was stirred at 80 °C for 1 hour in argon atmosphere until complete dissolution was achieved. The temperature was then reduced to 60 °C, and 4-nitrophthalonitrile (4 mmol) was added. After 2 hours the reaction mixture was poured into 100 mL of water, and the resulting precipitation was immediately filtered and washed with cold water (3 × 30 mL). The obtained white solid was then subjected to lyophilization for 16 hours to ensure complete removal of residual solvents.

4,4'-(((((1E,1'E)-[2,2'-Bifuran]-5,5'-diylbis(methaneylylidene))bis(azaneylylidene))bis(4,1-phenylene))bis(oxy))diphthalonitrile, (BF-AP-PN, 11), (Error! Reference source not found.**).**

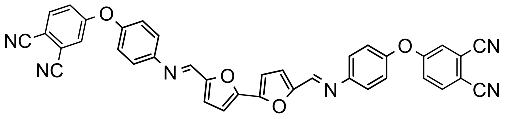

11 BF-AP-PN

Scheme S 15. Schematic representation of the chemical structure of BF-AP-PN (**11**).

BF-AP-PN (**11**) was synthesized according to the procedure described above, BF-AP (**7**) and 4-nitrophthalonitrile were taken as starting materials. Light yellow powder was obtained; yield is 81%. T_{mp} = 251 °C (DSC and TGA: Figure S25).

^{1}H NMR (300 MHz, DMSO-d6) *δ* ppm: 7.20 - 7.24 (m, 2H), 7.24 - 7.29 (m, 4H), 7.34 - 7.38 (m, 2H), 7.40 - 7.43 (m, 1H), 7.43 - 7.51 (m, 5H), 7.84 (d, J=2.51 Hz, 2H), 8.10 - 8.16 (m, 2H), 8.55 - 8.58 (m, 2H) (Figure S21).

^{13}C NMR (75 MHz, DMSO-d6) *δ* ppm: 108.67, 111.24, 114.99, 115.87, 116.38, 117.18, 121.66, 122.50, 123.18, 123.72, 136.80, 147.47, 148.87, 152.52, 152.61, 161.60, 189.88 (Figure S22).

MS (ESI, positive mode): m/z $[2M+Na]^{2+}$ = 1272, M = 624. (Figure S24)

4-(4-(((E)-(5-(2-(5-((E)-((4-(3,4-Dicyanobenzyl)phenyl)imino)methyl)furan-2-yl)propan-2-yl)furan-2-yl)methylene)amino)phenoxy)phthalonitrile, (BFA-AP-PN, 12), (Error! Reference source not found.**).**

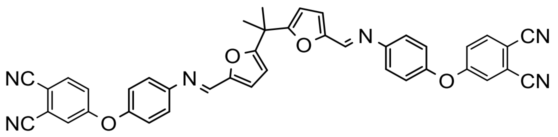

12 BFA-AP-PN

Scheme S 16. Schematic representation of the chemical structure of BFA-AP-PN (**12**).

BFA-AP-PN (**12**) was synthesized according to the procedure described above, BFA-AP (**8**) and 4-nitrophthalonitrile were taken as starting materials. Light yellow powder was obtained; yield is 78%. T_g = 75 °C (DSC and TGA: Figure S30).

^{1}H NMR (300 MHz, DMSO-d6) *δ* ppm: 1.68 - 1.80 (m, 6H), 6.53 - 6.61 (m, 2H), 7.11 - 7.17 (m, 2H), 7.18 - 7.26 (m, 4H), 7.32 - 7.43 (m, 6H), 7.77 - 7.85 (m, 2H), 8.08 - 8.15 (m, 2H), 8.37 - 8.42 (m, 2H) (Figure S26).

^{13}C NMR (75 MHz, DMSO-d6) *δ* ppm: 26.39, 38.09, 108.59, 108.64, 115.86, 116.38, 117.15, 119.34, 121.58, 122.39, 123.09, 123.53, 136.78, 149.05, 149.26, 151.46, 152.21, 161.66, 162.94 (Figure S27).

MS (ESI, positive mode): m/z 689.00 (*$[M + Na]^+$*), M = 666.00. (Figure S29)

4,4'-((((((1E,1'E)-[2,2'-Bifuran]-5,5'-diylbis(methaneylylidene))bis(azaneylylidene))bis(ethane-2,1-diyl))bis(4,1-phenylene))bis(oxy))diphthalonitrile, (BF-Ty-PN, 13), (Error! Reference source not found.**)**

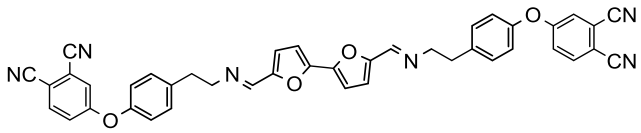

13 BF-Ty-PN

Scheme S 17. Schematic representation of the chemical structure of BF-Ty-PN (**13**).

BF-Ty-PN (**13**) was synthesized according to the procedure described above, BF-Ty (**9**) and 4-nitrophthalonitrile were taken as starting materials. Light yellow powder was obtained; yield is 84%. T_g = 65 °C (DSC and TGA: Figure S35).

^{1}H NMR (300 MHz, DMSO-d6) *δ* ppm: 2.92 - 3.06 (m, 4H), 3.74 - 3.91 (m, 4H), 6.94 - 7.08 (m, 4H), 7.09 - 7.17 (m, 4H), 7.28 - 7.44 (m, 6H), 7.75 (d, J=2.51 Hz, 1H), 7.69 - 7.79 (m, 1H), , 8.03 - 8.18 (m, 4H) (Figure S31).

^{13}C NMR (75 MHz, DMSO-d6) *δ* ppm: 36.50, 108.39, 109.79, 115.87, 116.38 (s, 2H), 117.06, 117.12, 120.71, 122.13, 122.93, 131.42, 136.73, 137.97, 146.70, 150.38, 151.77, 152.37, 161.77 (Figure S32).

MS (ESI, positive mode): m/z 681.2 (*$[M + H]^+$*), M = 680.00. (Figure S34)

4,4'-((((((1E,1'E)-(Propane-2,2-diylbis(furan-5,2-diyl))bis(methaneylylidene))bis(azaneylylidene))bis(ethane-2,1-diyl))bis(4,1-phenylene))bis(oxy))diphthalonitrile, (BFA-Ty-PN, 14), (Error! Reference source not found.**).**

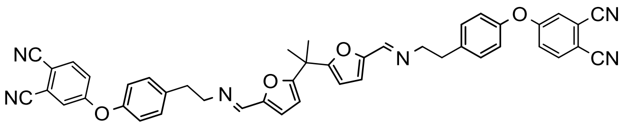

14 BFA-Ty-PN

Scheme S 18. Schematic representation of the chemical structure of BFA-Ty-PN (**14**).

BFA-Ty-PN (**14**) was synthesized according to the procedure described above, BFA-Ty (**10**) and 4-nitrophthalonitrile were taken as starting materials. Light yellow powder was obtained; yield is 82%. T_g = 51 °C (DSC and TGA: Figure S40).

^{1}H NMR (300 MHz, DMSO-d6) *δ* ppm: 1.54 - 1.74 (m, 6H), 2.82 - 3.01 (m, 4H), 3.64 - 3.83 (m, 4H), 6.30 - 6.42 (m, 2H), 6.78 - 6.87 (m, 2H), 7.01 - 7.20 (m, 4H), 7.24 - 7.43 (m, 6H), 7.68 - 7.80 (m, 2H), 7.99 - 8.13 (m, 4H) (Figure S36).

^{13}C NMR (75 MHz, DMSO-d6) *δ* ppm: 26.36, 36.63, 37.79, 62.40, 107.62, 108.38, 115.56, 116.38, 117.11, 120.68, 122.12 122.90, 131.37, 136.72, 138.03, 150.68, 150.95, 152.33, 161.67, 161.76 (Figure S37).

MS (ESI, positive mode): m/z 723.3 (*[M + H]*$^+$), M = 722. (Figure S39)

Curing process of phthalonitriles resins. To investigate the curing process of the resins, monomers **11**-**14** were mixed with 10 mol% of APN (**1**) using a mortar and pestle. The mixture was then placed in a Schlenk flask, melted (110 °C) and degassed at this temperature under low vacuum (<0.05 mbar) during 6 hours. The mixture was analyzed by ^{1}H NMR to confirm the absence of solvent. The hot mixture was poured into a mold (a porcelain dish, a 2 mL vial, or an aluminum mold). Before placing the samples in the tube furnace for curing, their mass was measured. At each stage of curing the samples were weighed to determine mass loss.

Thermal decomposition calculation.

Method assessment: Firstly, calculations for basis set assessment were performed for the decomposition path with the breaking C-C bond between furan rings (Scheme S 19) of **11** monomer using various basis sets, namely 6-31G(d)[6], 6-31G(d,p)[7], 6-311G(d,p)[8], 6-311+G(d,p)[8], cc-pVDZ[9] and cc-pVTZ[10]. The corresponding decomposition path and the calculated ΔH and ΔG values are present in Scheme 3 and Figure S1, respectively. The results indicate that the addition of polarization functions and the use of a triple-zeta basis set are crucial, while including diffuse functions doesn't significantly change the results. Therefore, for all further analyses, the B3LYP-D3(BJ)/6-311G(d,p) level of theory was used.

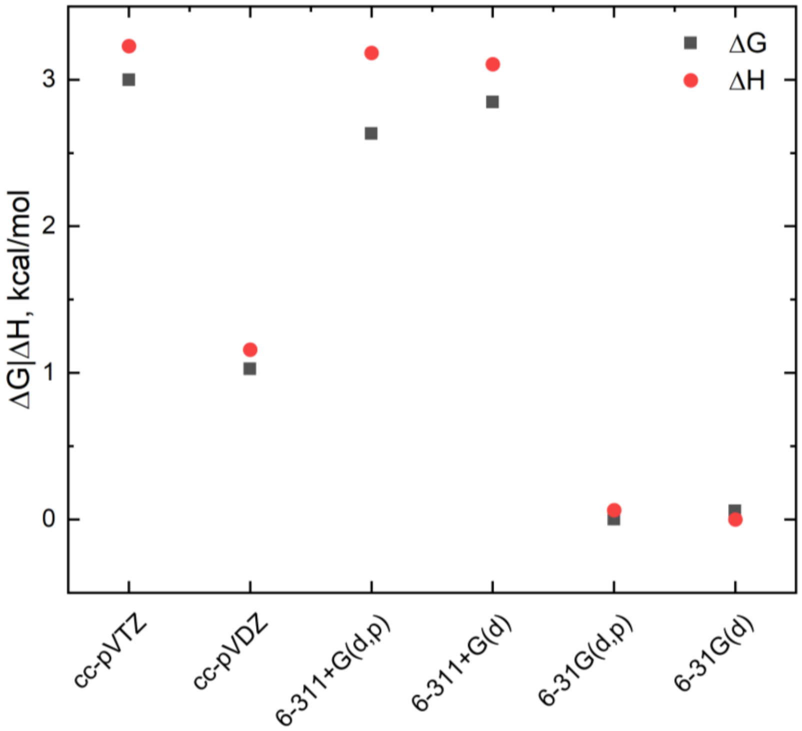

Figure S 1. Comparison of the calculated enthalpies (ΔH), and Gibbs free energies (ΔG) for the reaction in Figure 3 using B3LYP-D3(BJ) with different basis sets.

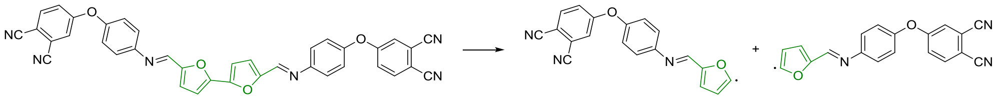

Scheme S 19. Schematic representation of decomposition reaction of BF-AP-PN molecule, used for basis set assessment.

Table SI1. The enthalpies (H) and Gibbs free energies (G) of the monomers and decomposed optimized fragments corresponding to the bond cleavage calculated at B3LYP-D3(BJ)/6-311G(d,p) level of theory: 1. BF-AP-PN (**11**), 2. BF-APA-PN (**12**), 3. Dimer of BF-AP-PN, 4. Dimer of BFA-AP-PN.

		***H*, Ha**		***G*, Ha**	
		**T=298 K**	**T=653 K**	**T=298 K**	**T=653 K**
1	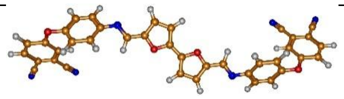	-2089.498213	-2089.375049	-2089.623005	-2089.819889
1.1	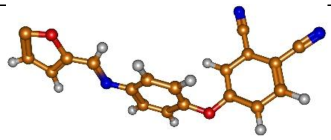	-1044.641122	-1044.579987	-1044.714749	-1044.826401
1.2	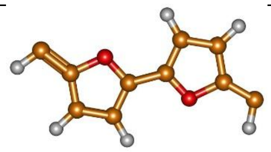	-534.877912	-534.845924	-534.924244	-534.991923

1.3	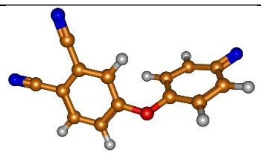	-777.05948	-777.014064	-777.119350	-777.014064
1.4	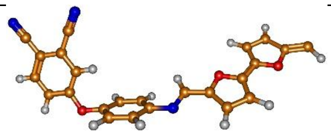	-1312.191959	-1312.114347	-1312.277777	-1312.410381
2	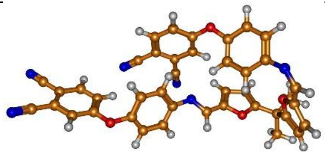	-2207.405057	-2207.26847	-2207.530733	-2207.733856
2.1	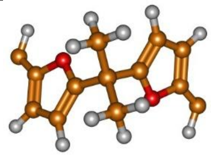	-652.782268	-652.73674	-652.839342	-652.925057
2.2	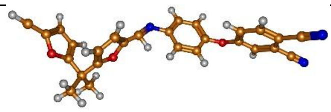	-1430.089058	-1429.997989	-1430.185101	-1430.335083
2.3	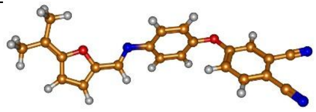	-1162.619725	-1162.545081	-1162.700399	-1162.825691
2.4	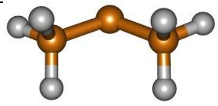	-117.762533	-117.749619	-117.793279	-117.834946
3	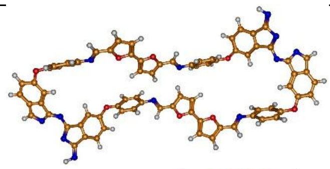	-4181.420241	-4181.173113	-4181.618361	-4181.950091
3.1	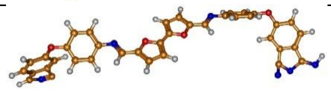	-2090.438458	-2090.313768	-2090.558912	-2090.750854
4	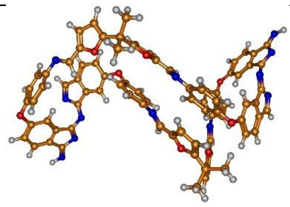	-4417.24063	-4416.966618	-4417.448473	-4417.80215

4.1	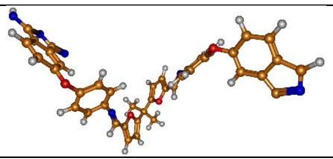	-2208.41536	-2208.277282	-2208.544898	-2208.752845

Figures

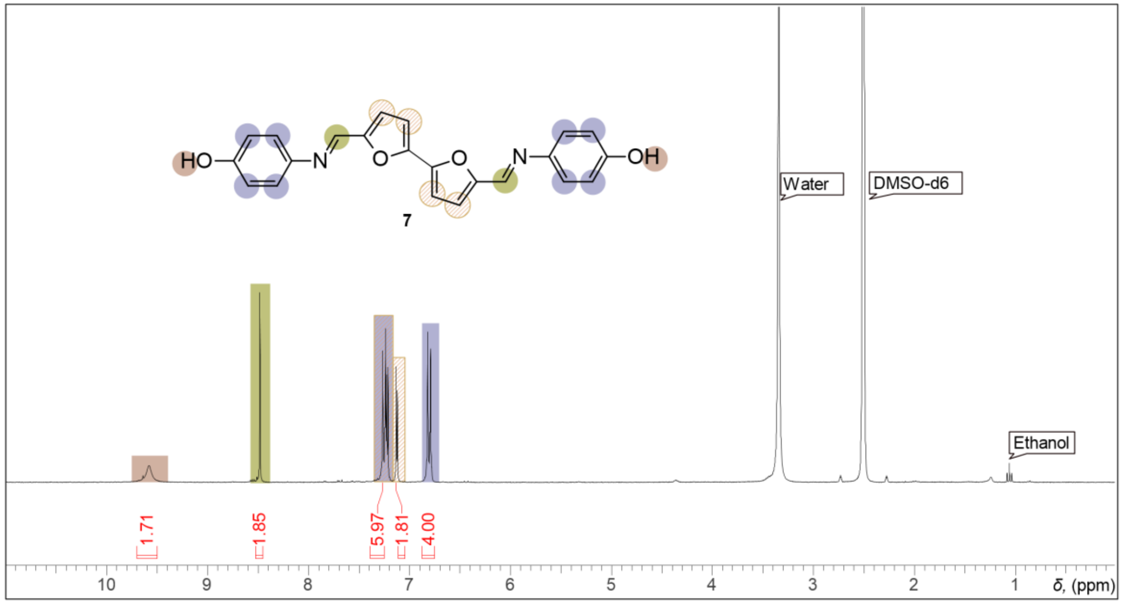

Figure S 2. ^{1}H NMR spectrum of BF-AP (**7**).

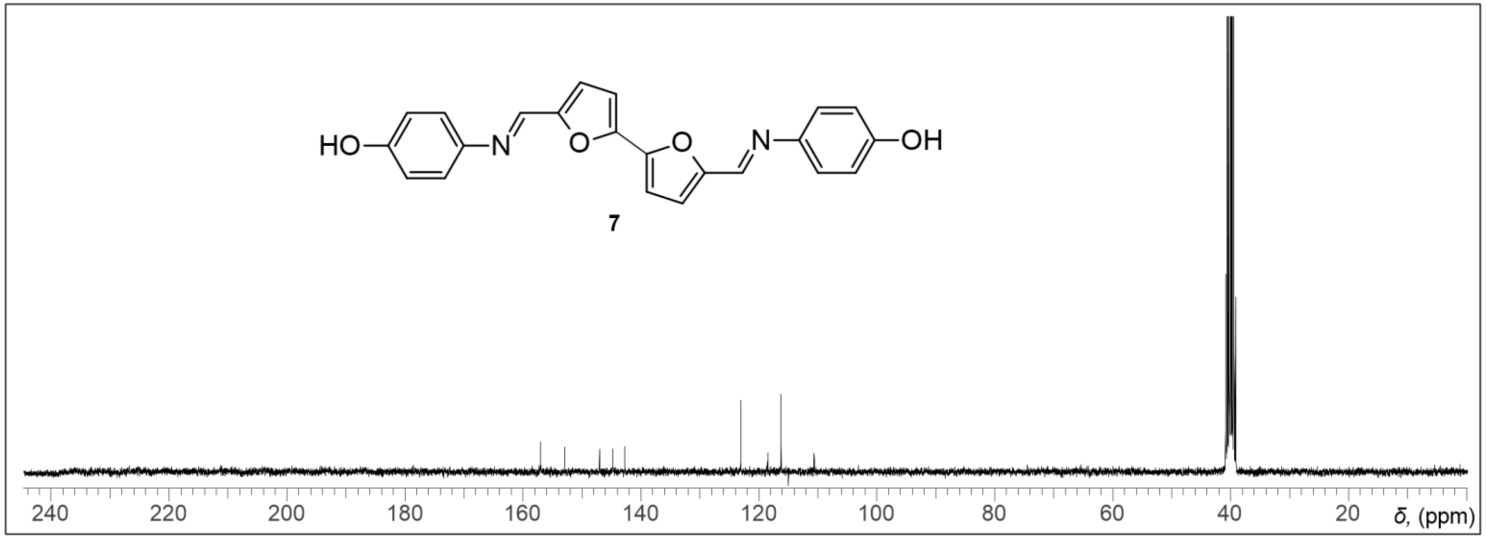

Figure S 3. ^{13}C NMR spectrum of BF-AP (**7**)

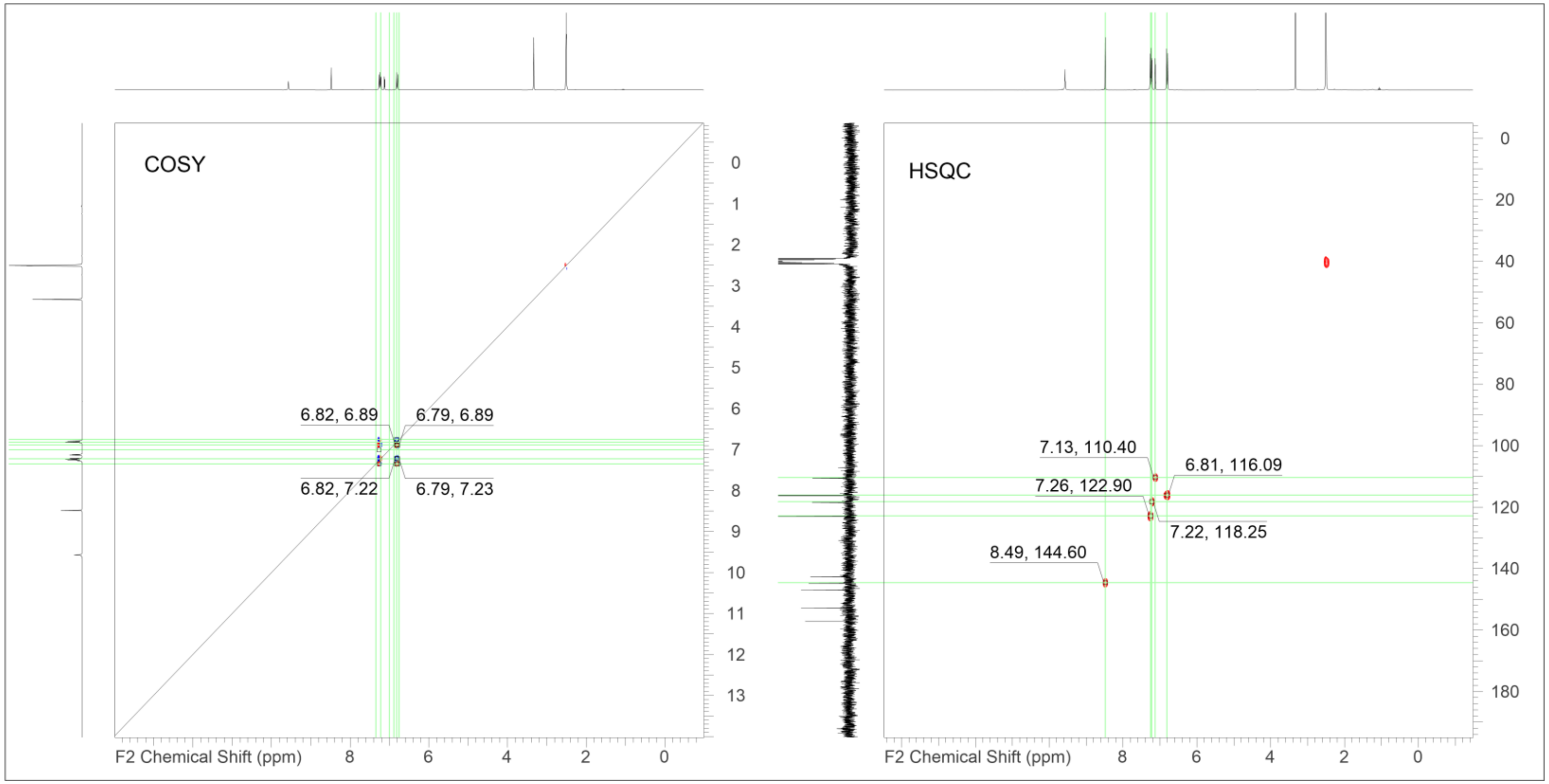

Figure S 4. COSY and HSQC spectra of BF-AP (**7**).

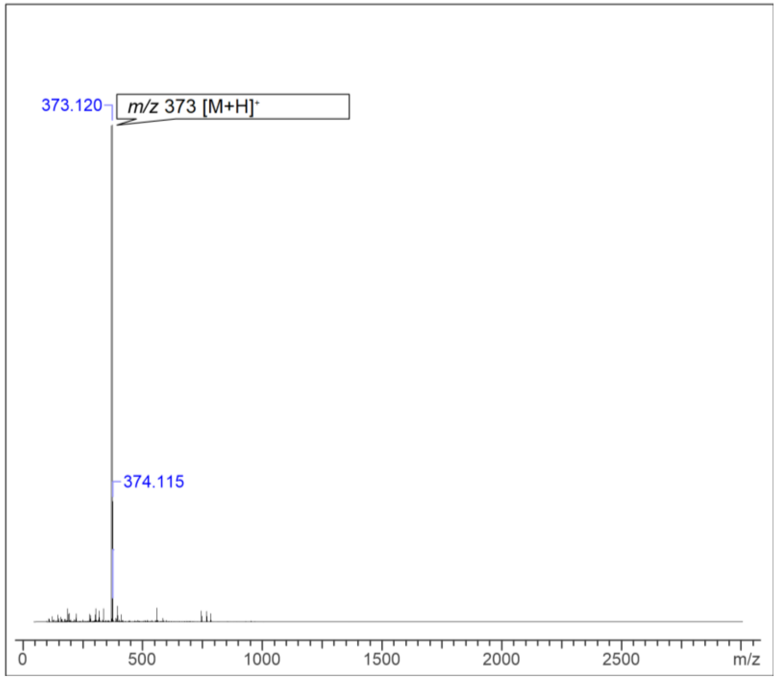

Figure S 5. ESI-MS spectrum of BF-AP (**7**).

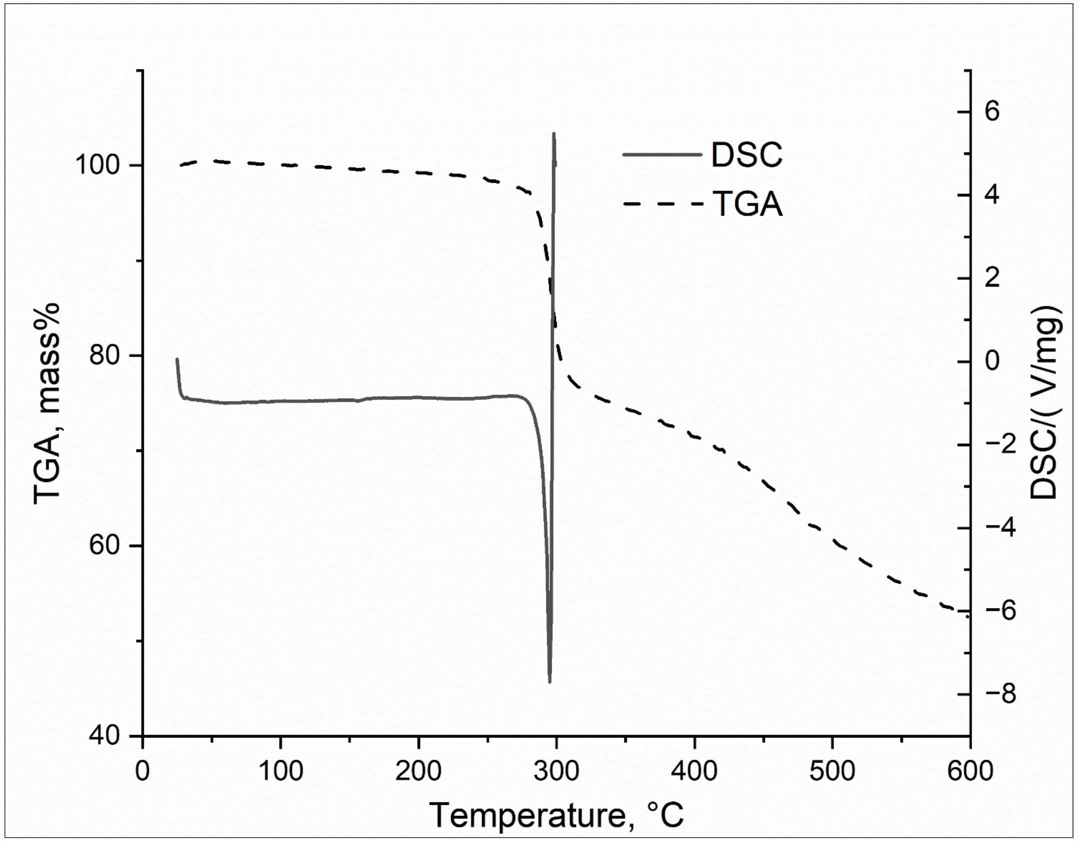

Figure S 6. TGA and DSC of BF-AP (**7**).

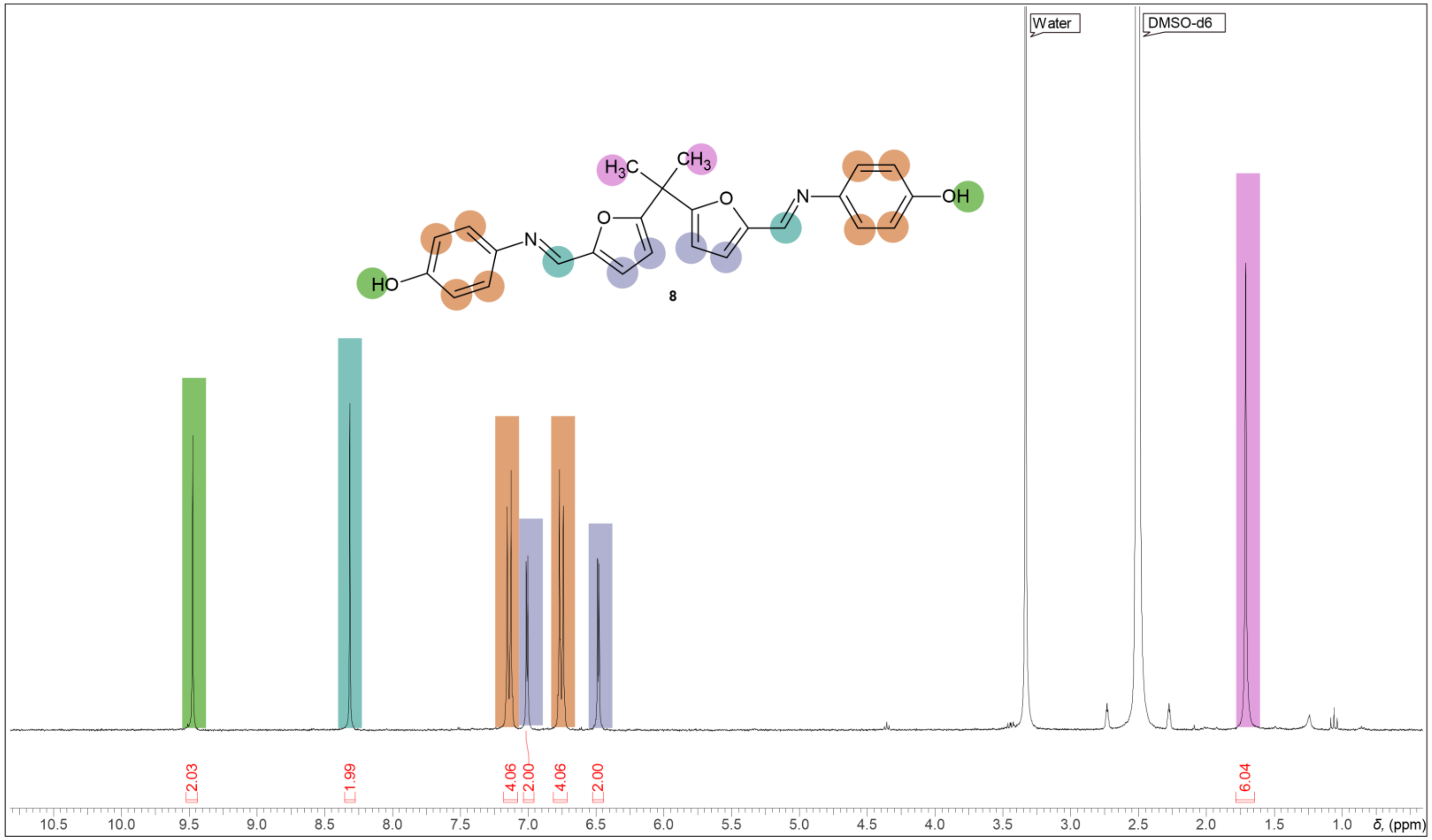

Figure S 7. ^{1}H NMR spectrum of BFA-AP (**8**).

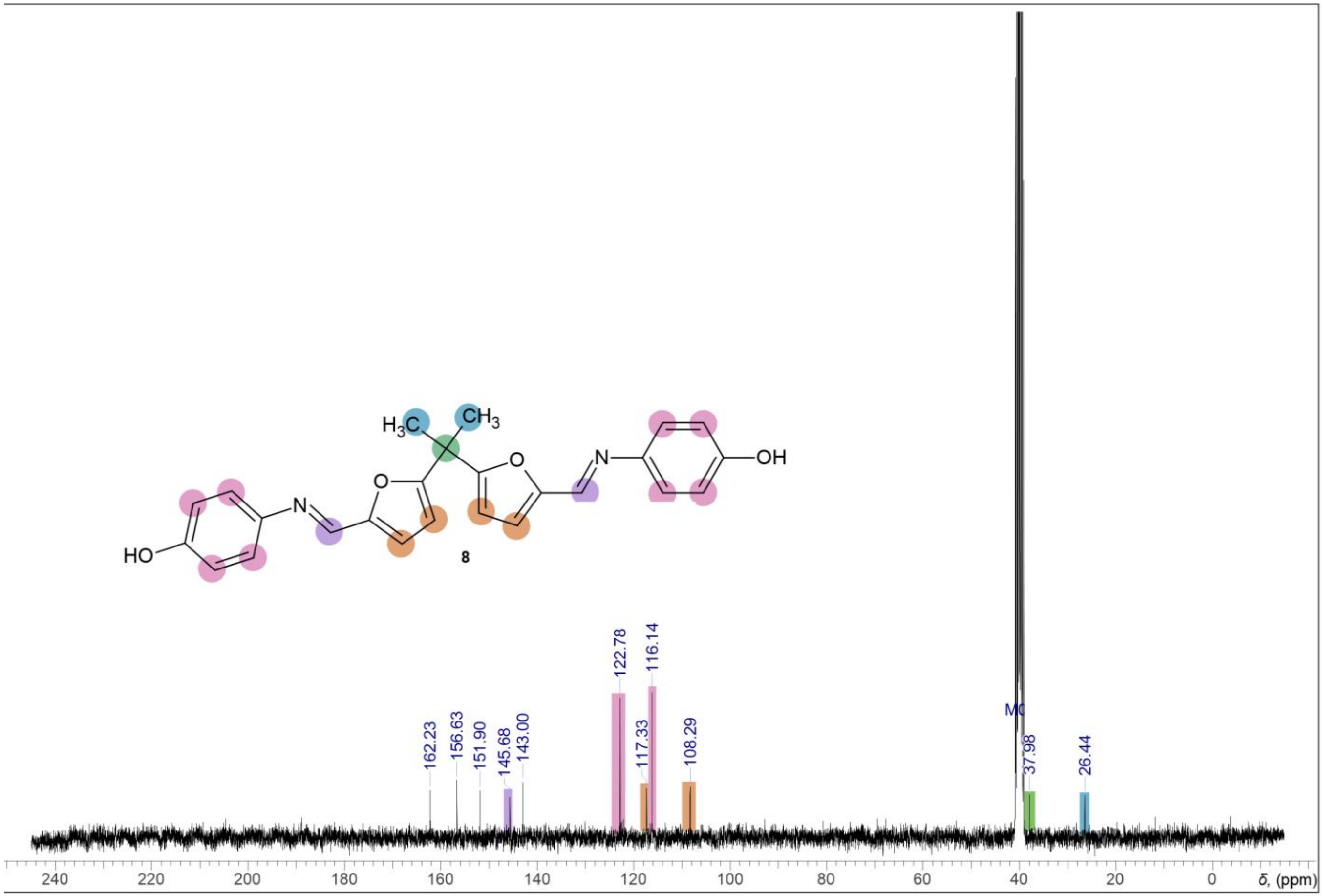

Figure S 8. ^{13}C spectrum of BFA-AP (**8**).

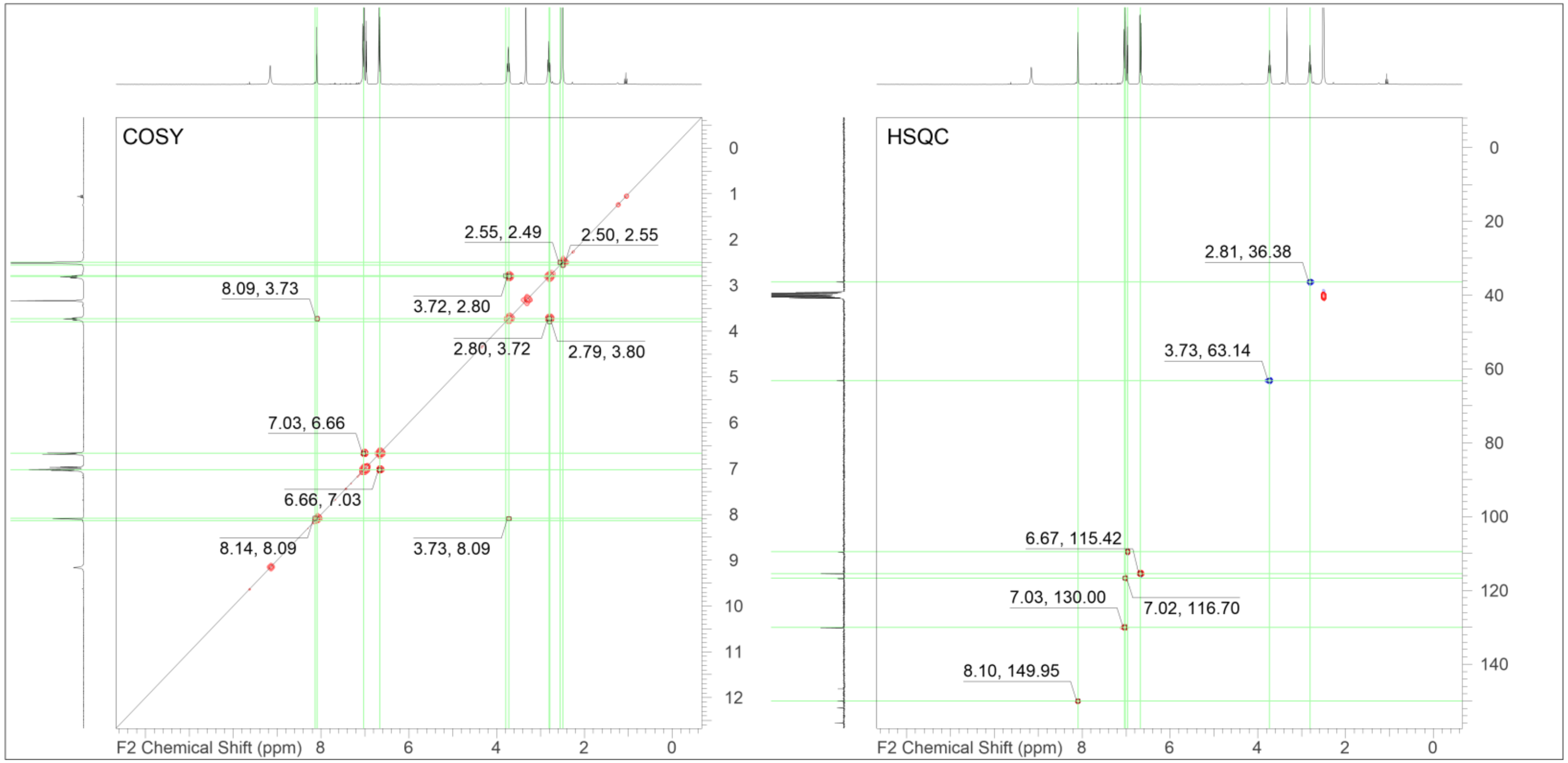

Figure S 9. COSY and HSQC spectra of BFA-AP (**8**).

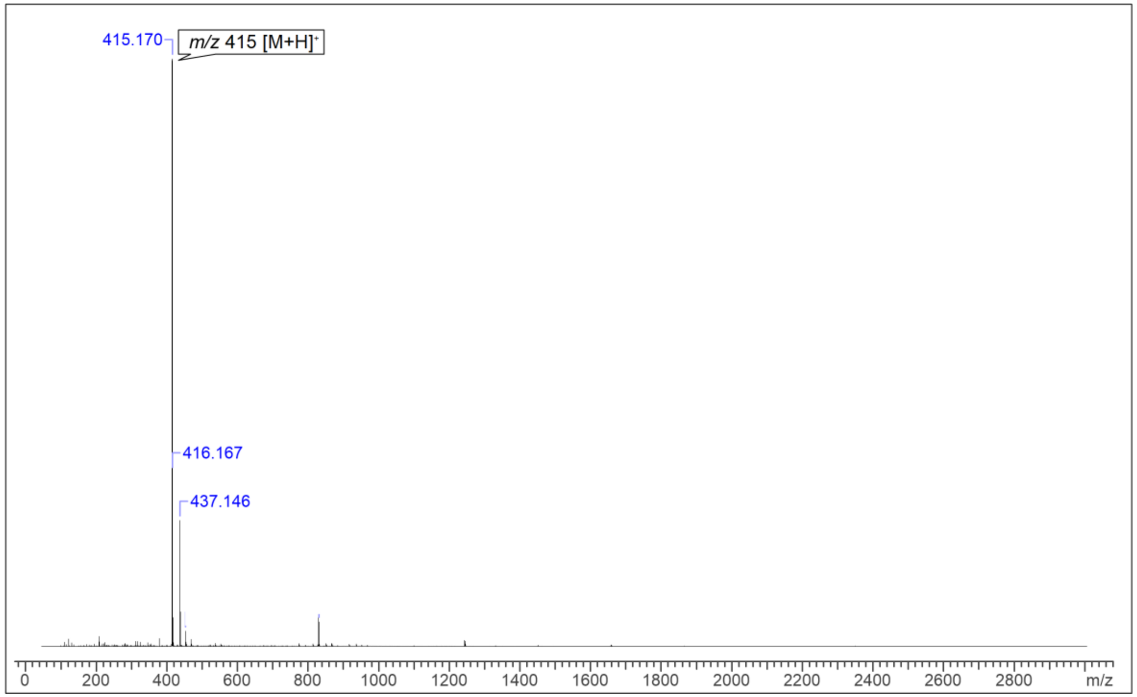

Figure S 10. ESI-MS spectrum of BFA-AP (**8**).

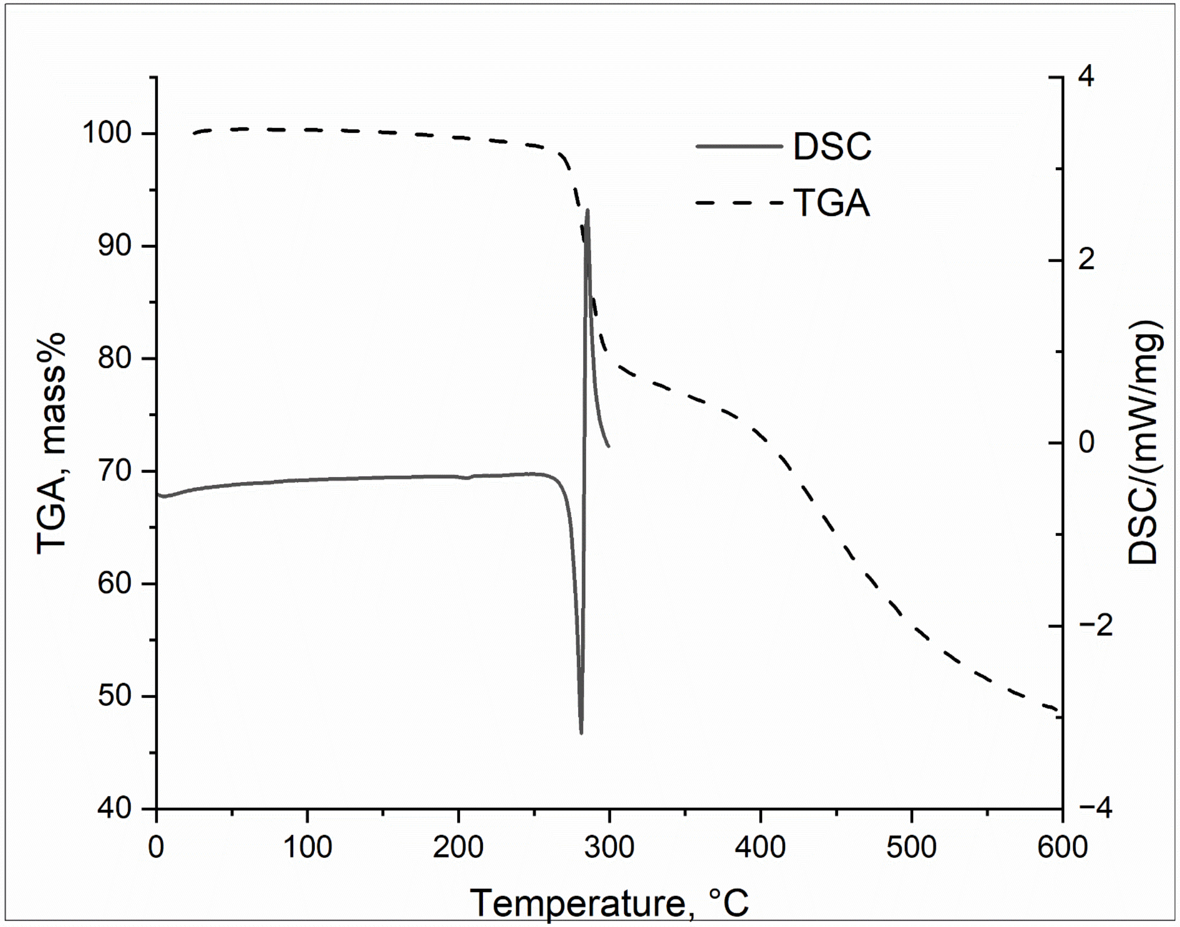

Figure S 11. TGA and DSC of BFA-AP (**8**).

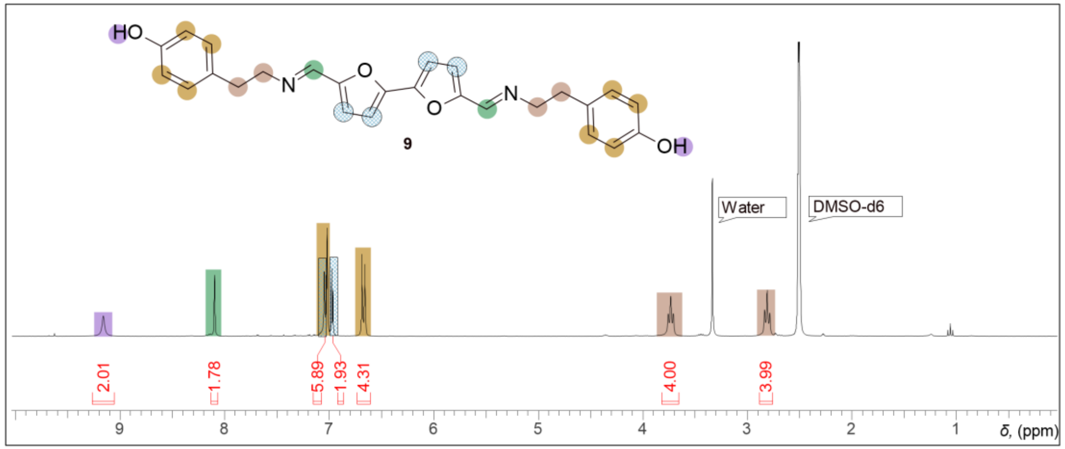

Figure S 12. ^{1}H NMR spectrum of BF-Ty (**9**).

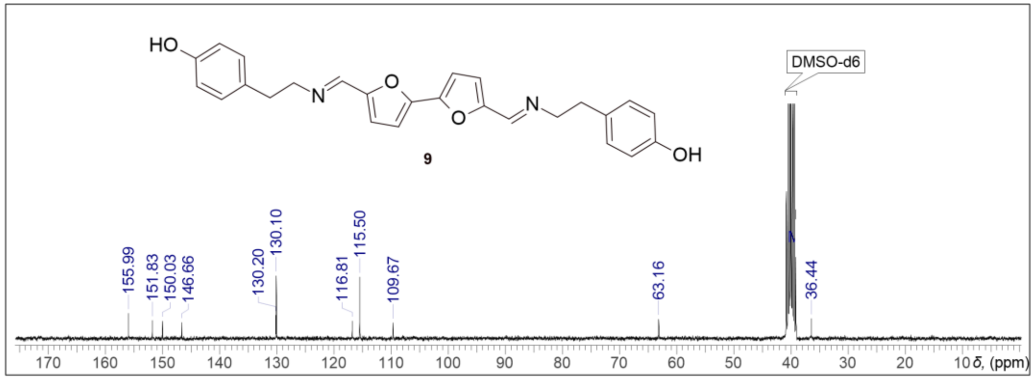

Figure S 13. ^{13}C NMR spectrum of BF-Ty (**9**).

Comment [uss1]: always DELTA Symbol in italic and the ppm in brackets

Comment [DP2]: Changed in all 1H 13C NMR spectra

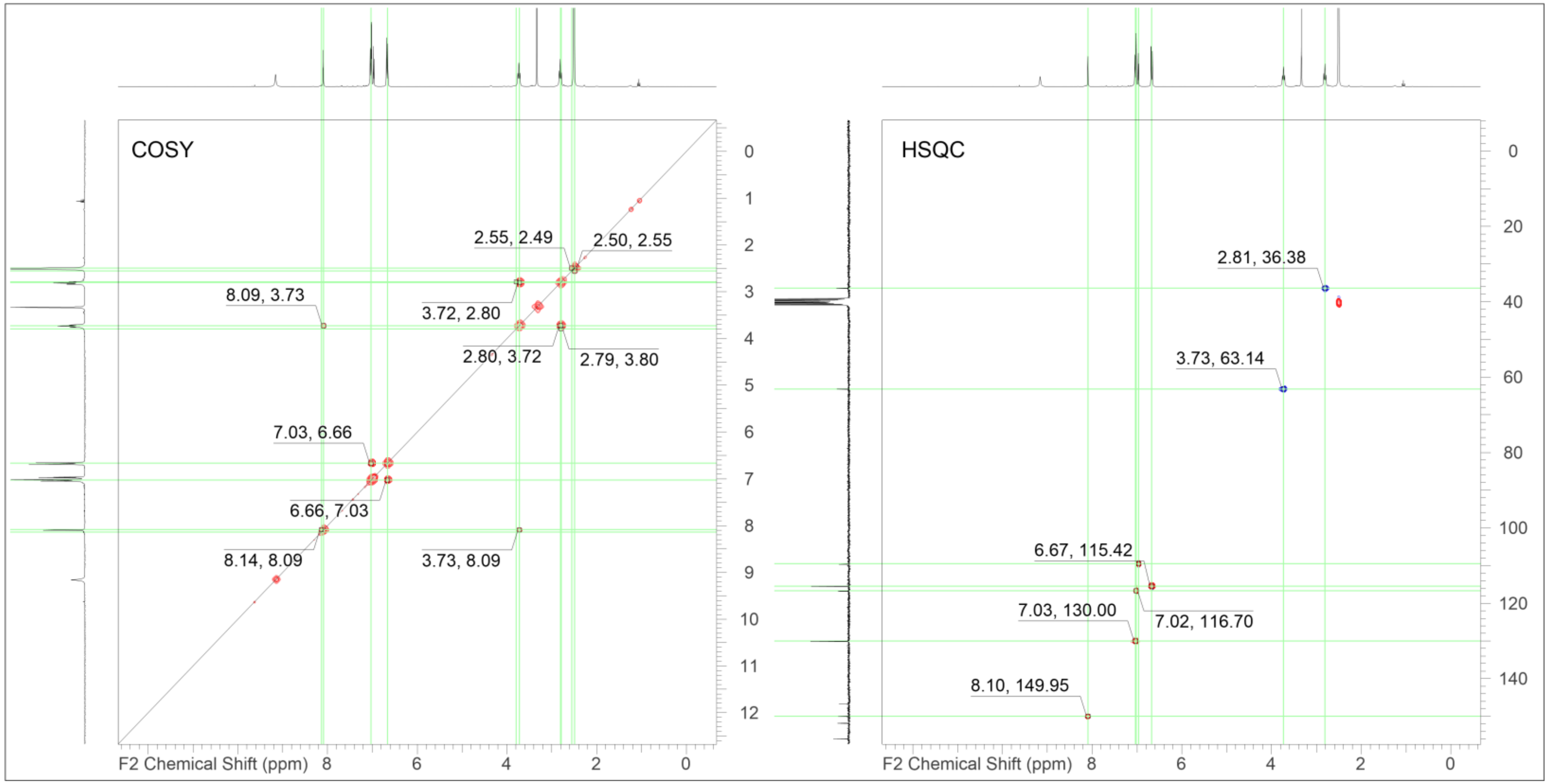

Figure S 14. COSY and HSQC spectra of BF-Ty (**9**).

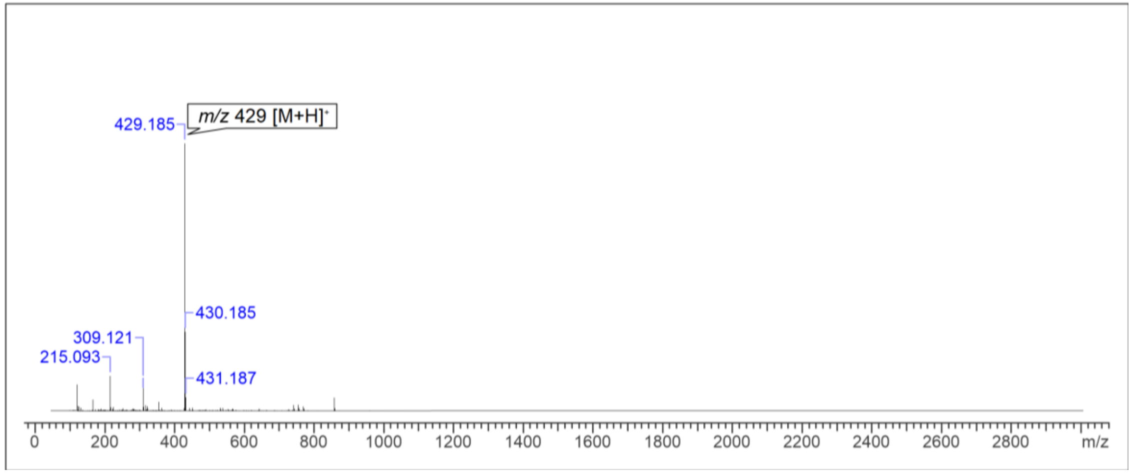

Figure S 15. ESI-MS spectrum of BF-Ty (**9**).

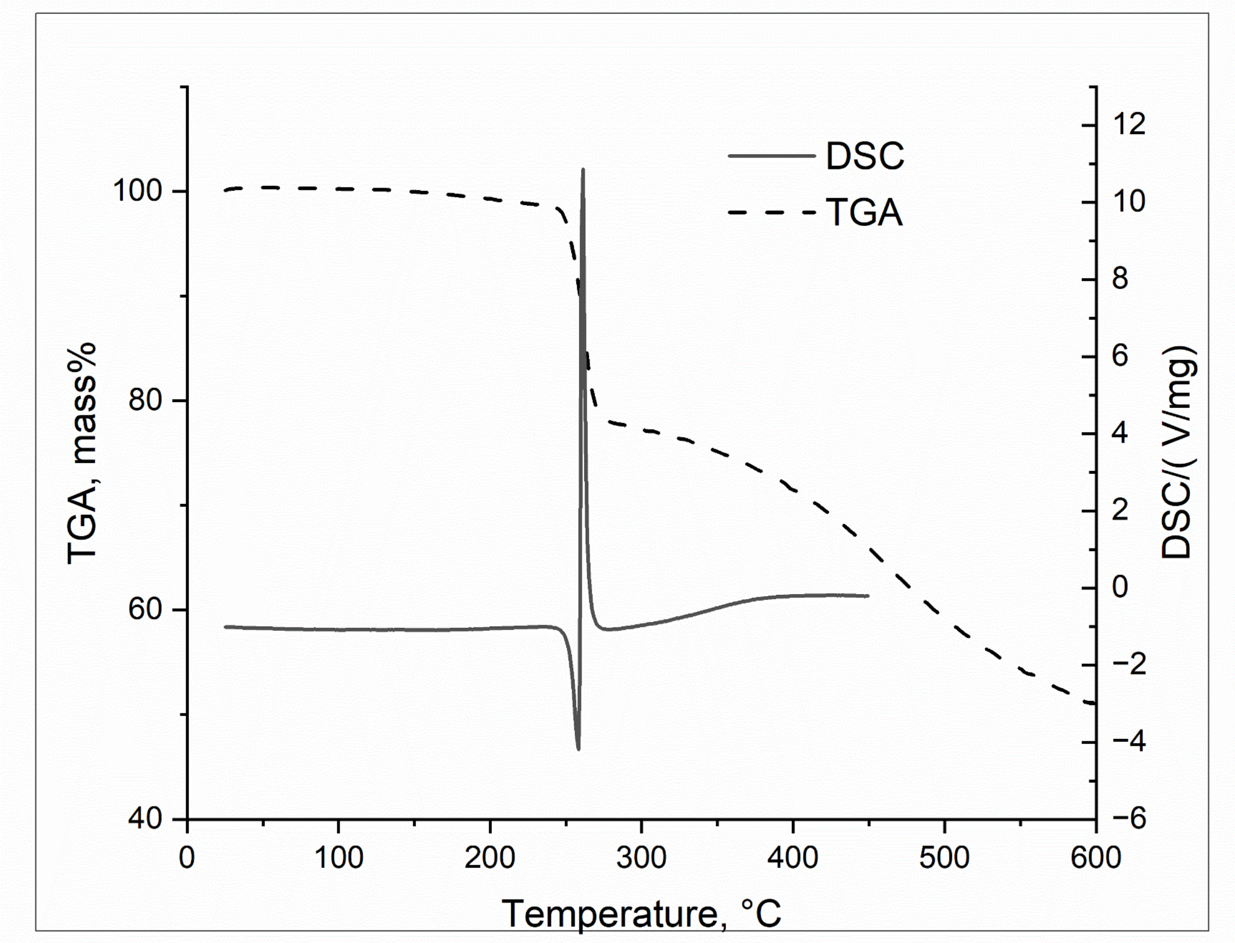

Figure S 16. TGA and DSC of BF-Ty (**9**).

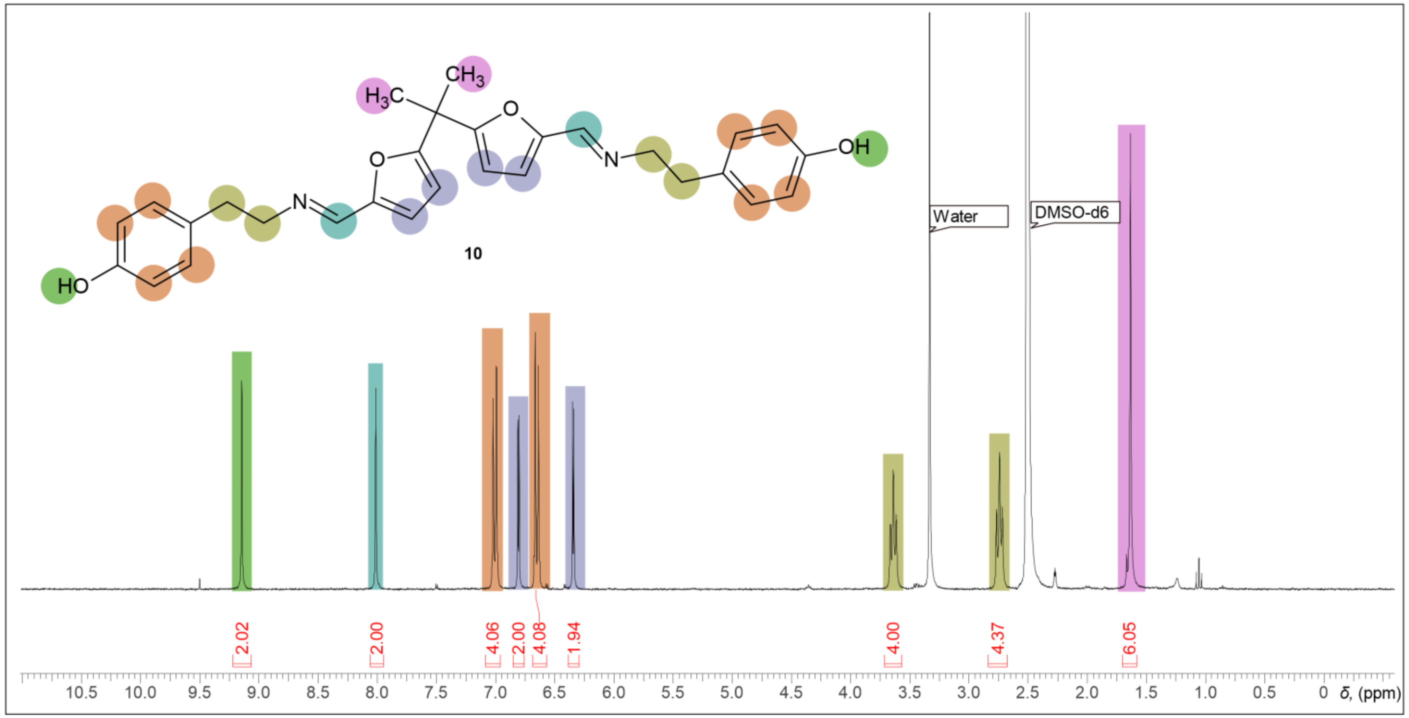

Figure S 17. ^{1}H NMR spectrum of BFA-Ty (**10**).

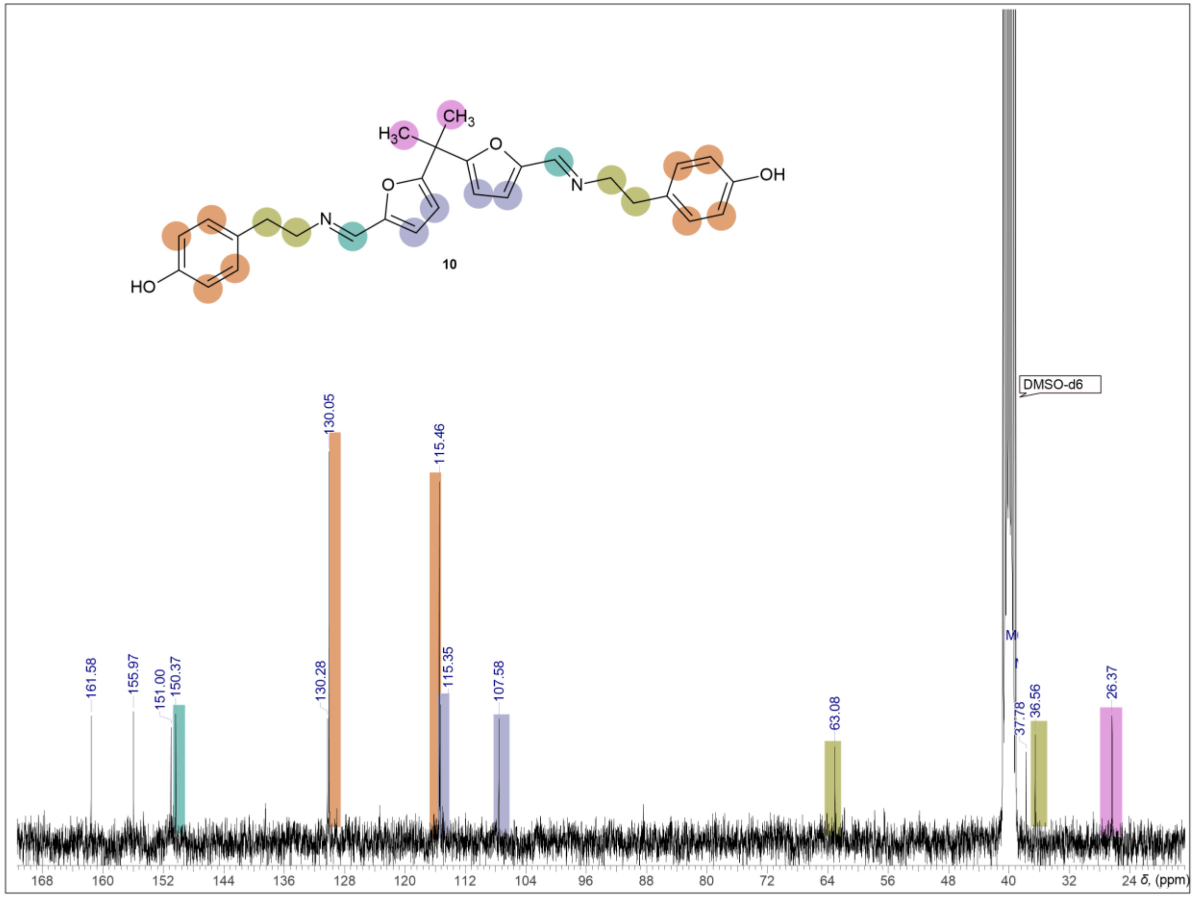

Figure S 18. ^{13}C NMR spectrum of BFA-Ty (**10**).

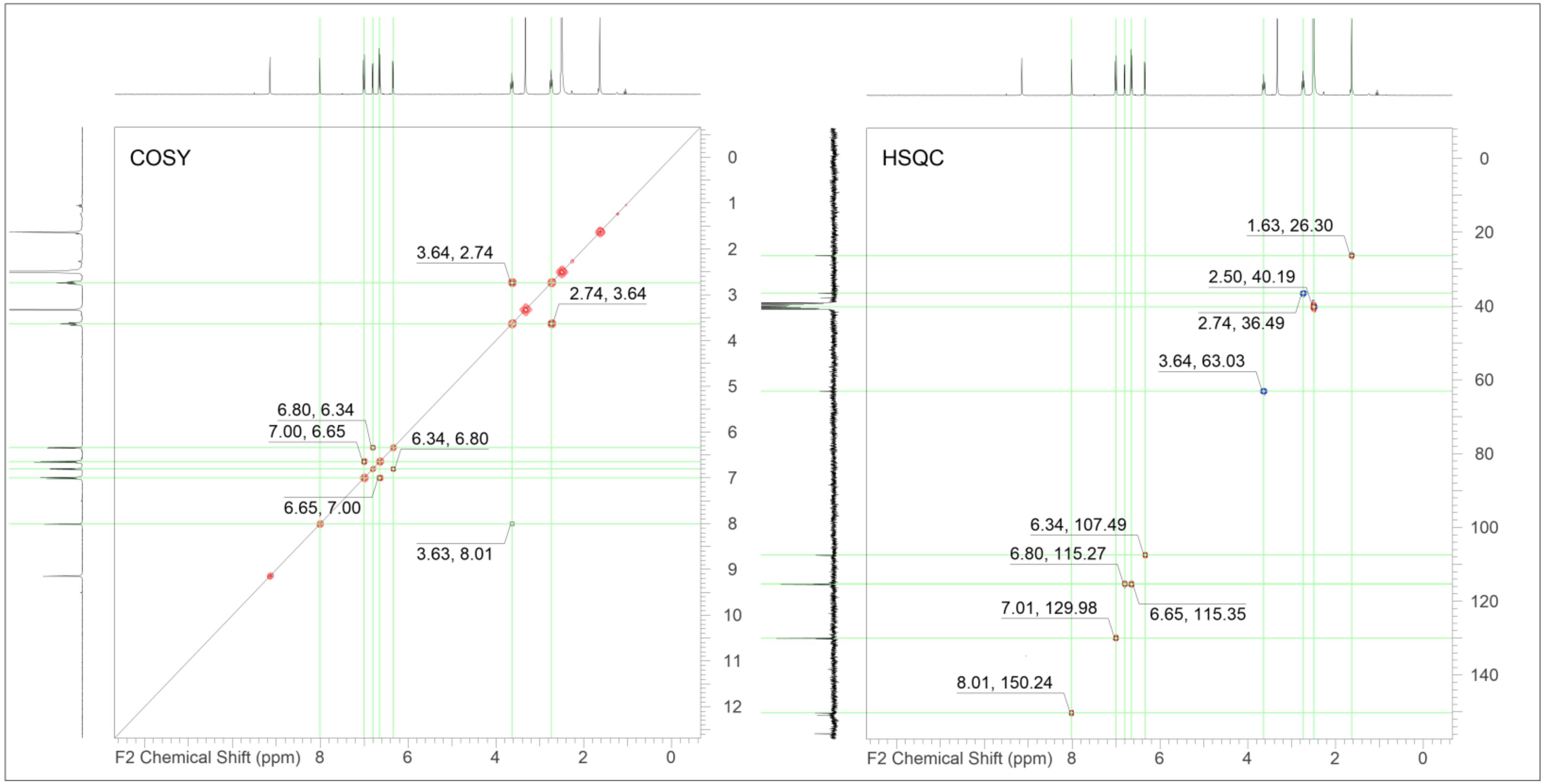

Figure S 19. COSY and HSQC spectra of BF-Ty (**10**).

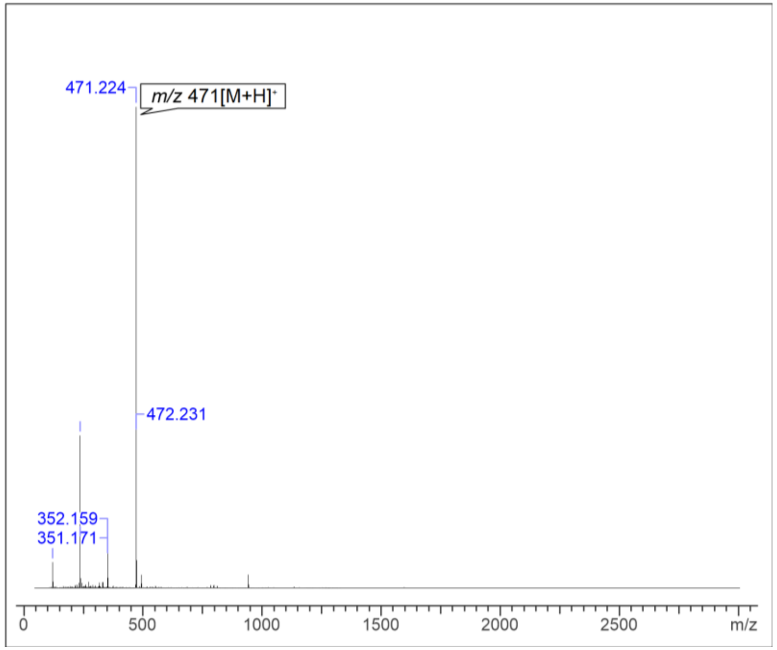

Figure S 20. ESI-MS spectrum of BFA-Ty (**10**).

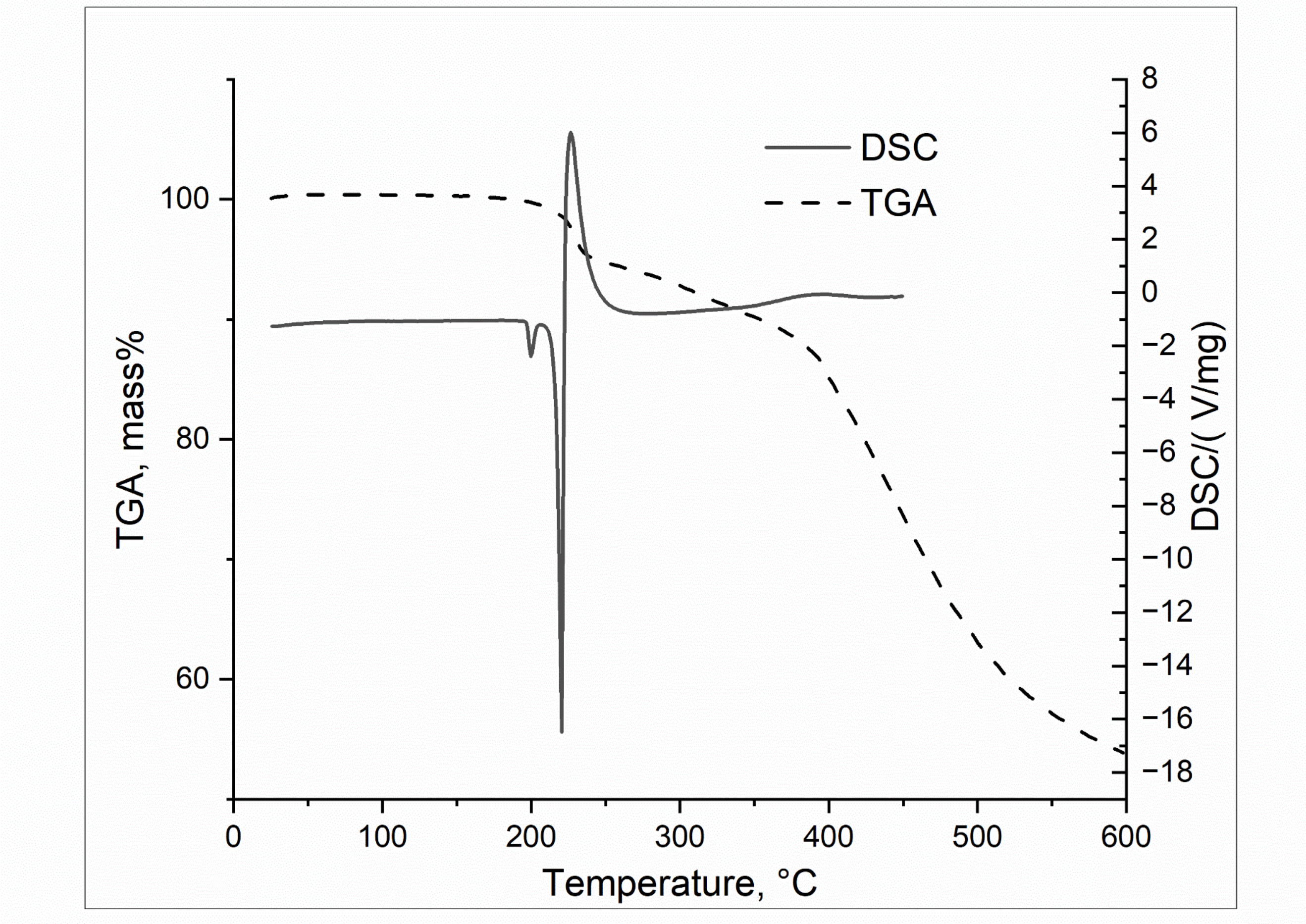

Figure S 21. TGA and DSC of BFA-Ty (**10**).

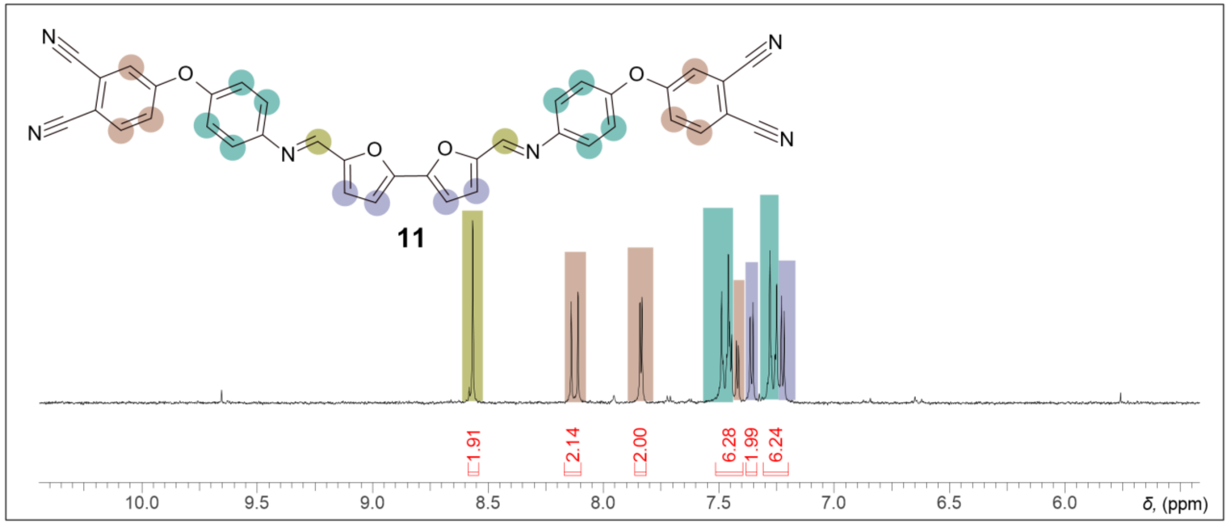

Figure S 22. ^{1}H NMR spectrum of BF-AP-PN (**11**).

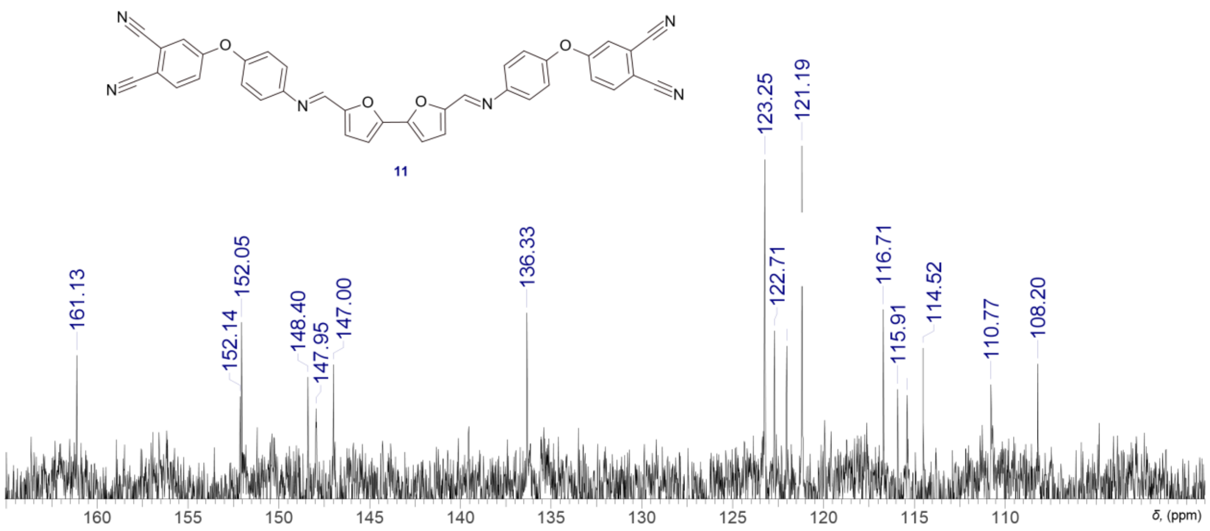

Figure S 23. ^{13}C spectrum of BF-AP-PN (**11**).

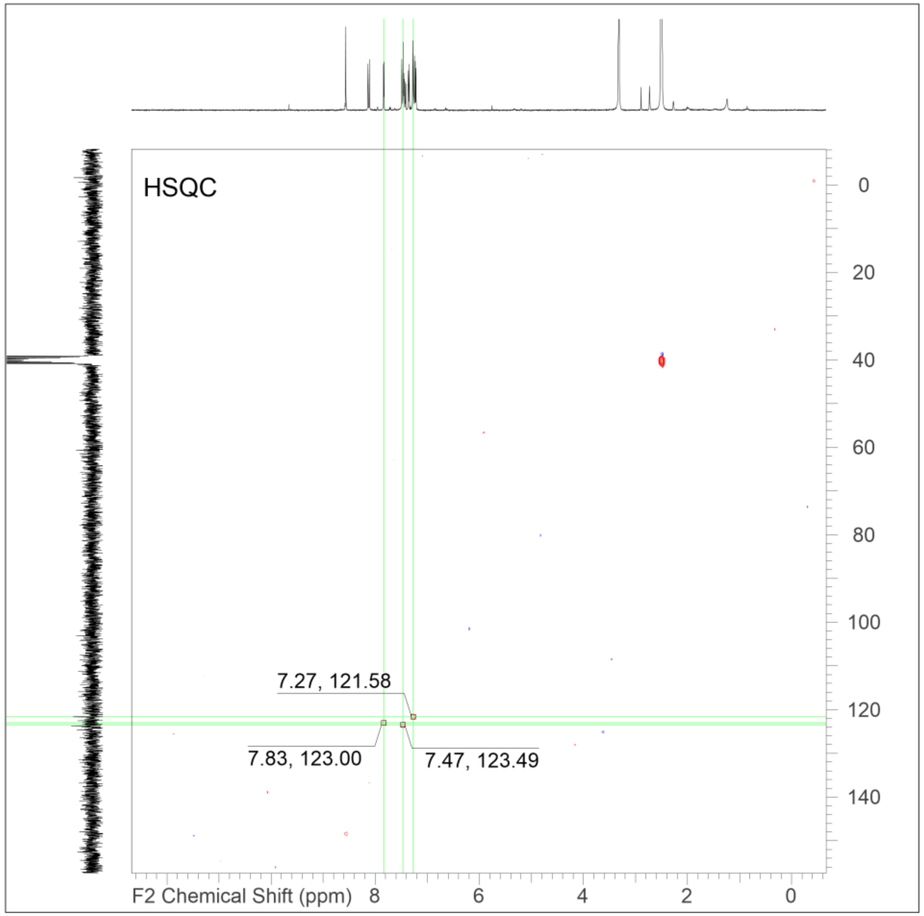

Figure S 24. HSQC spectrum of BF-AP-PN (**11**).

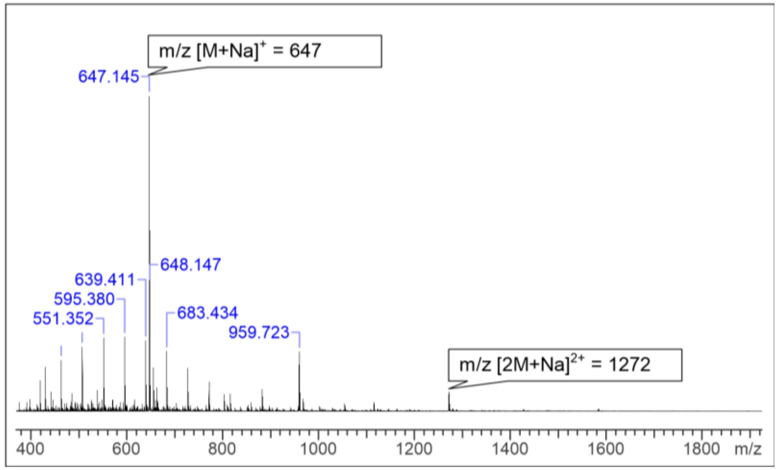

Figure S 25. ESI-MS spectrum of BF-AP-PN (**11**).

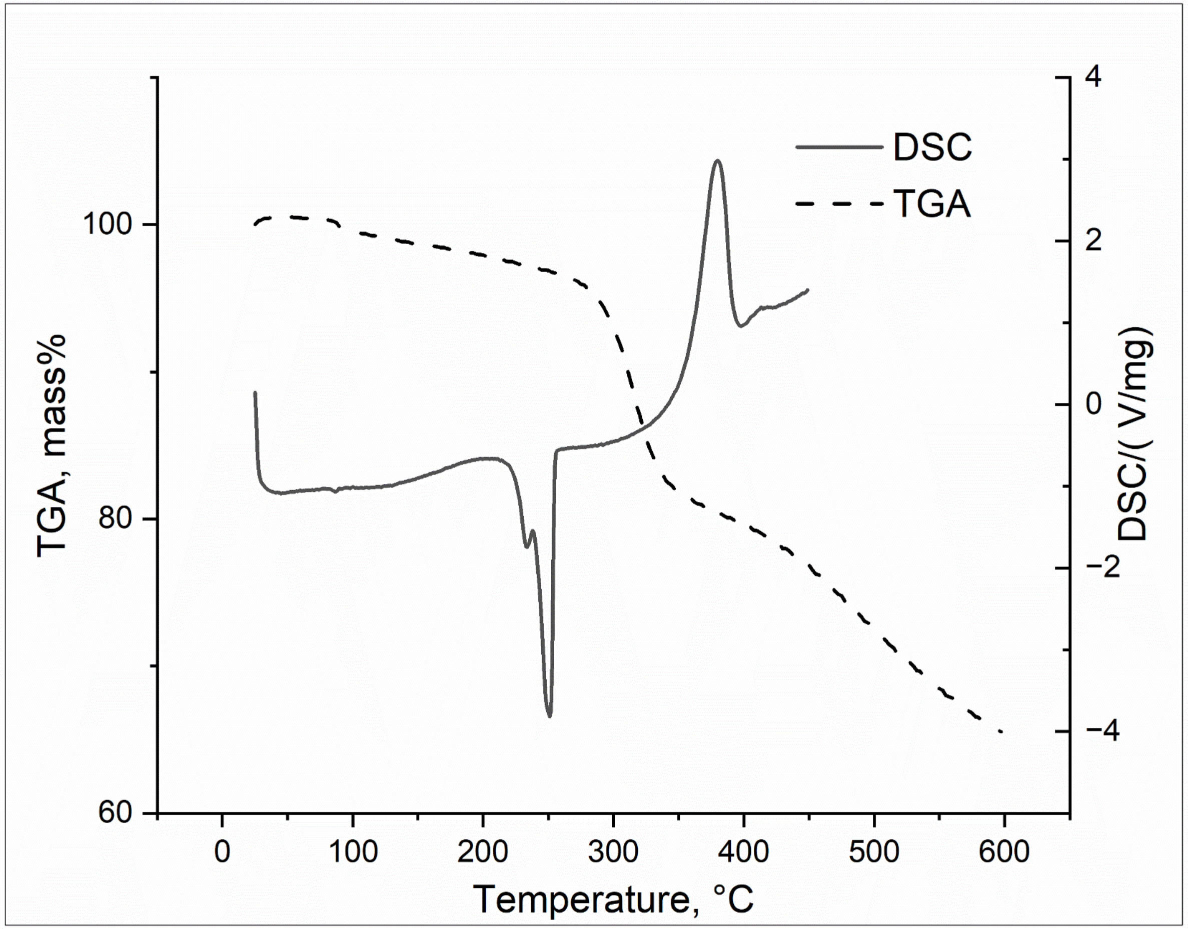

Figure S 26. TGA and DSC of BF-AP-PN (**11**).

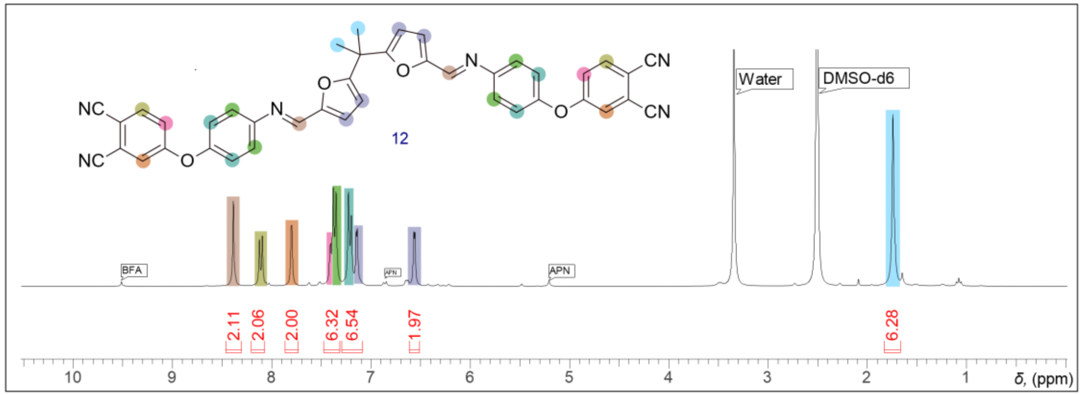

Figure S 27. ^{1}H NMR spectrum of BFA-AP-PN (**12**).

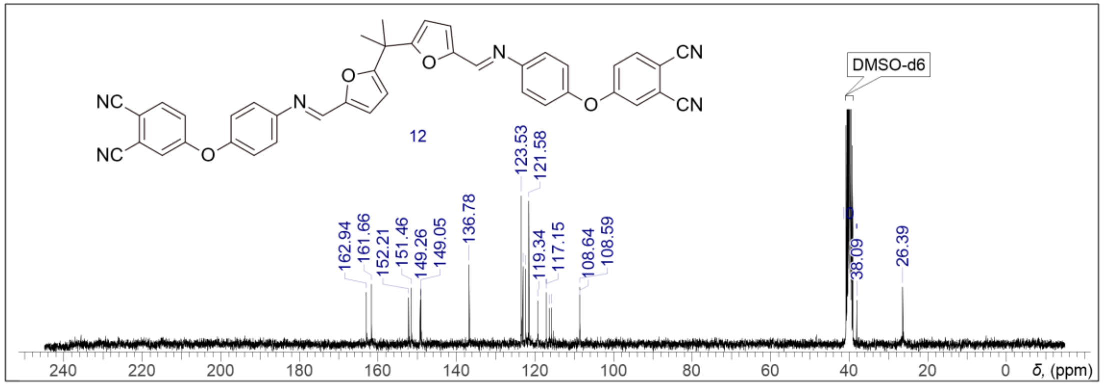

Figure S 28. ^{13}C NMR spectrum of BFA-AP-PN (**12**).

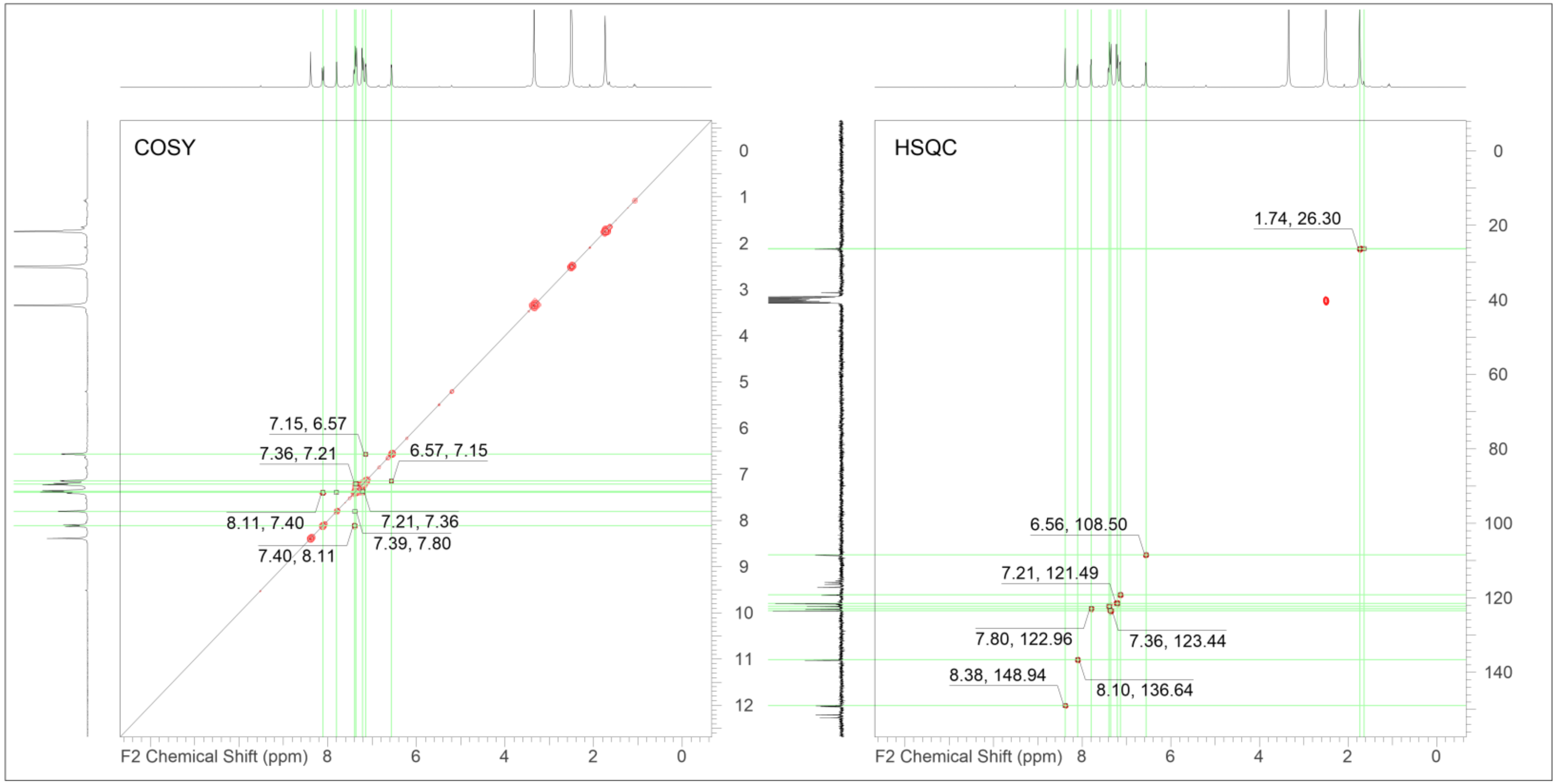

Figure S 29. COSY and HSQC spectrum of BFA-AP-PN (**12**).

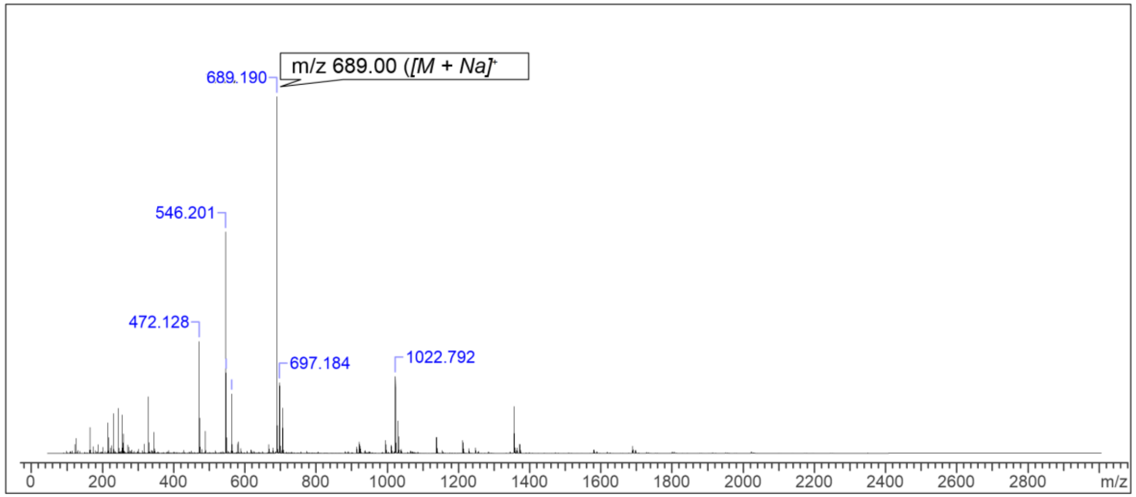

Figure S 30. ESI-MS spectrum of BFA-AP-PN (**12**).

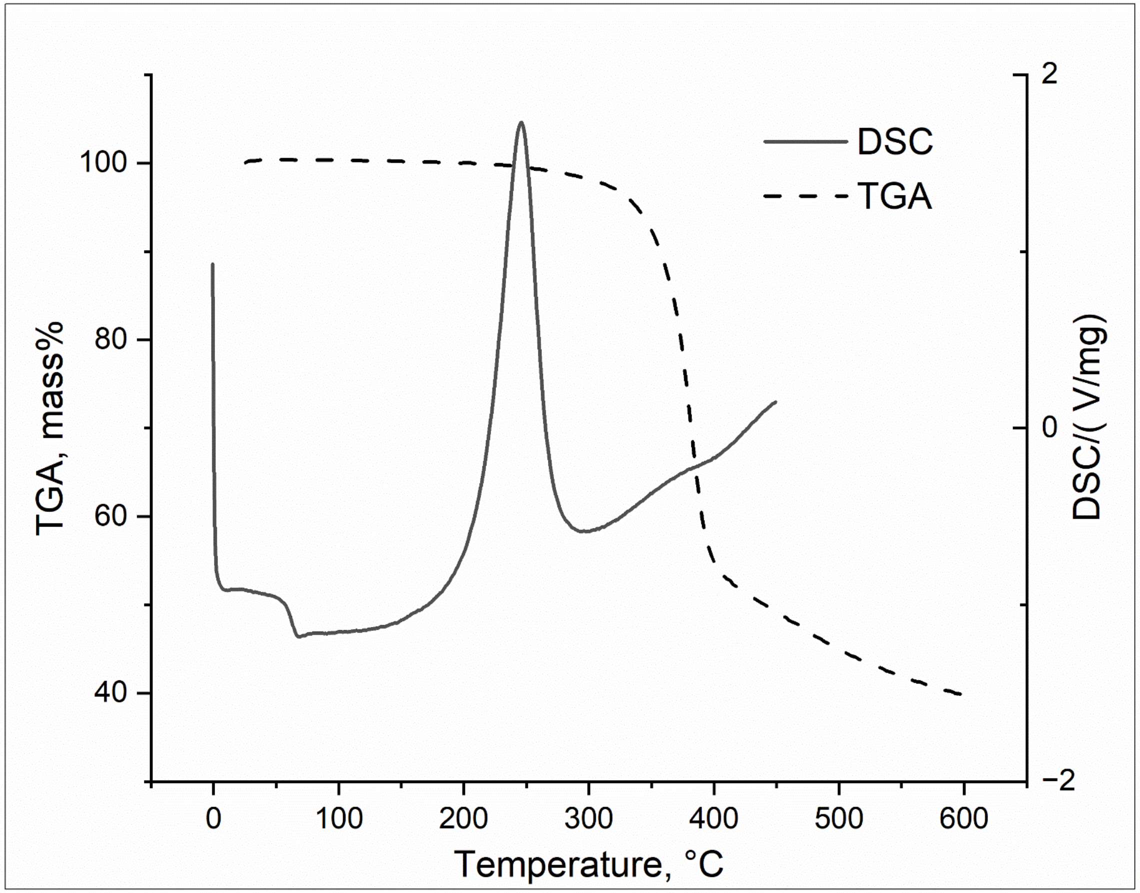

Figure S 31. TGA and DSC of BFA-AP-PN (**12**).

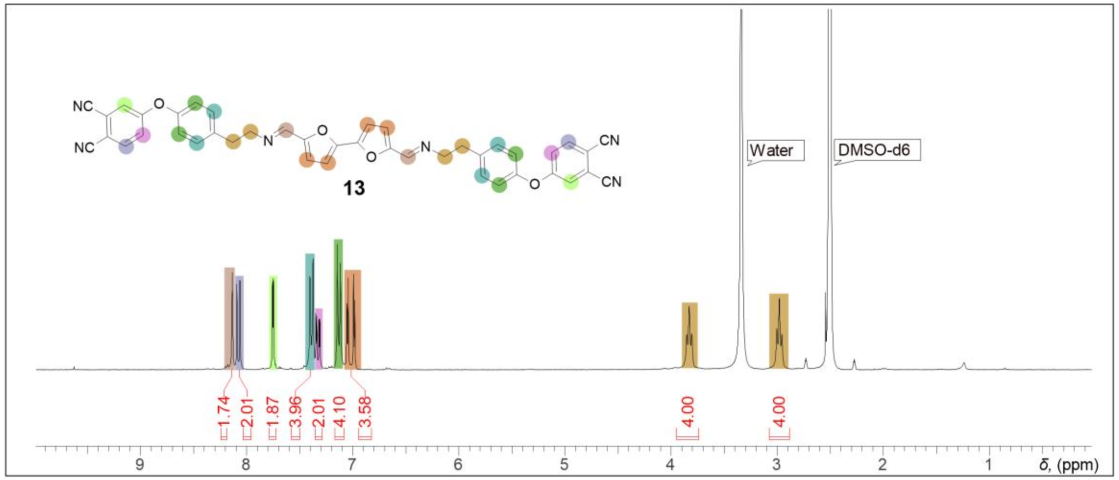

Figure S 32. ^{1}H NMR spectrum of BF-Ty-PN (**13**).

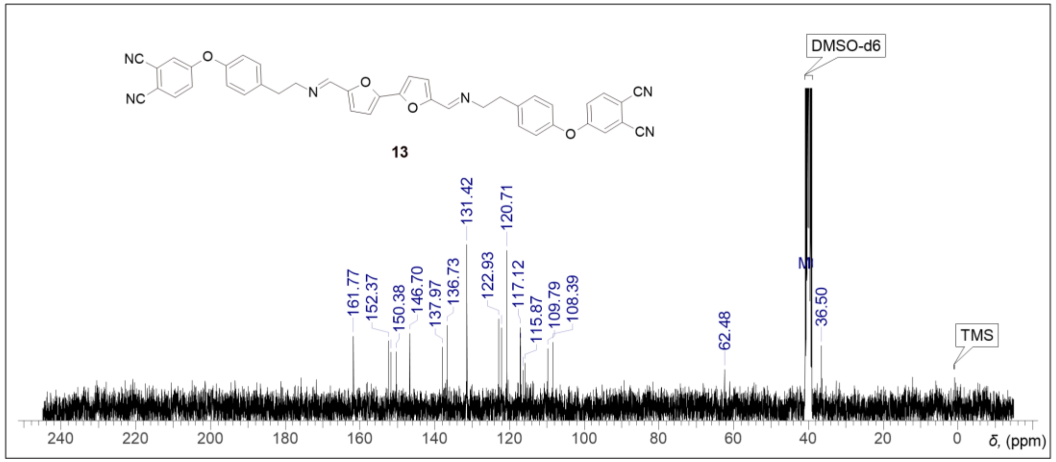

Figure S 33. ^{13}C NMR spectrum of BF-Ty-PN (**13**).

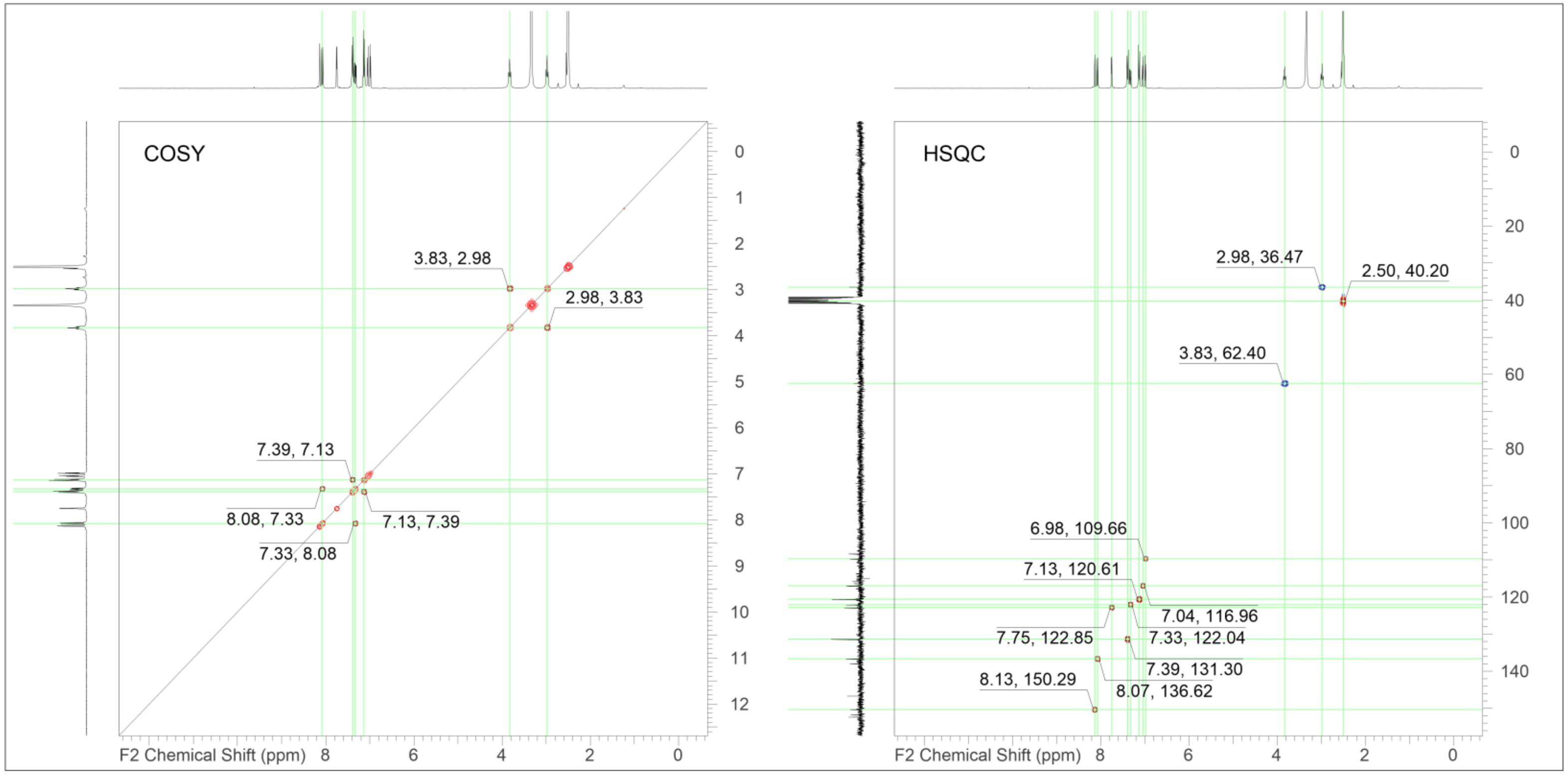

Figure S 34. COSY and HSQC spectra of BF-Ty-PN (**13**).

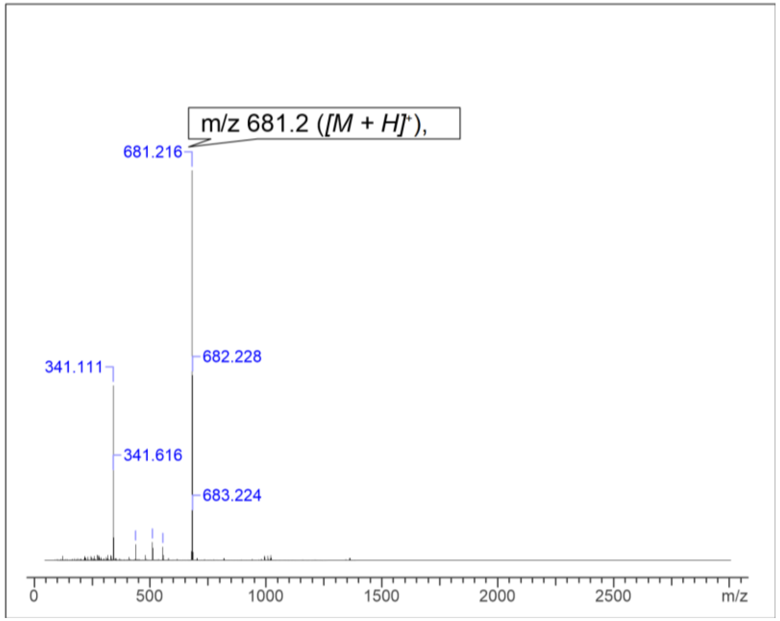

Figure S 35. ESI-MS spectrum of BF-Ty-PN (**13**).

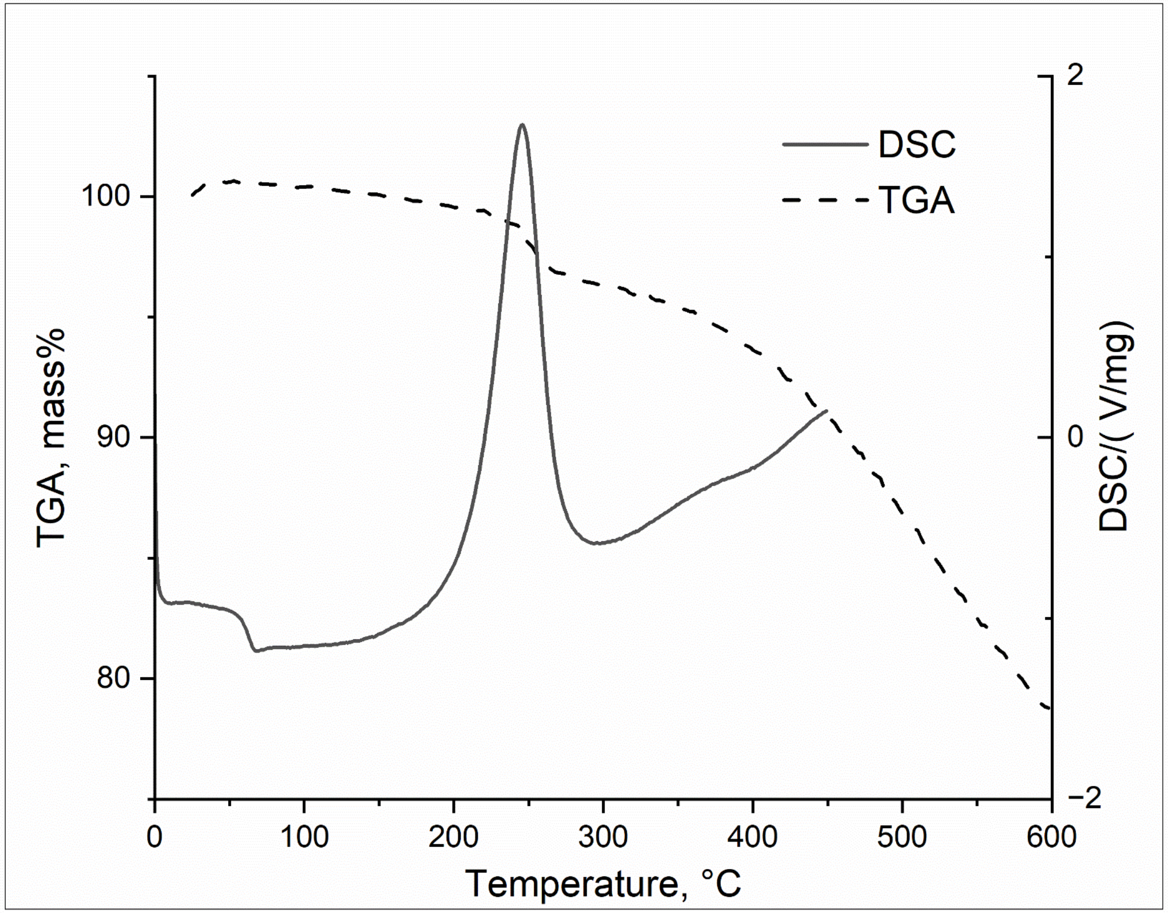

Figure S 36. TGA and DSC of BF-Ty-PN (**13**).

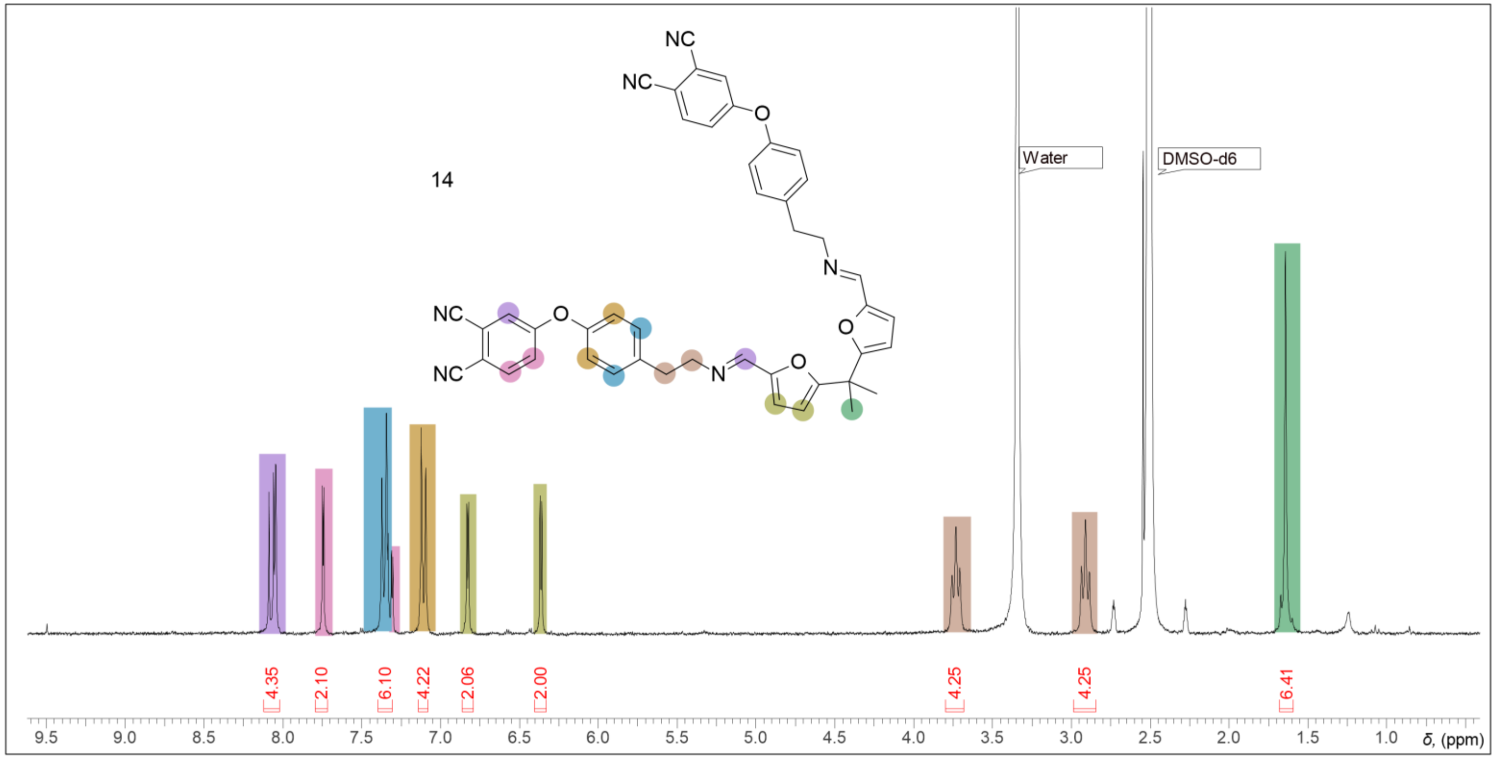

Figure S 37. ^{1}H NMR spectrum of BFA-Ty-PN (**14**).

Comment [BB3]: Order: 1H, 13C, COZY, HSQC, MS, DSC, TGA
No assigning of the signals without 2D!!!

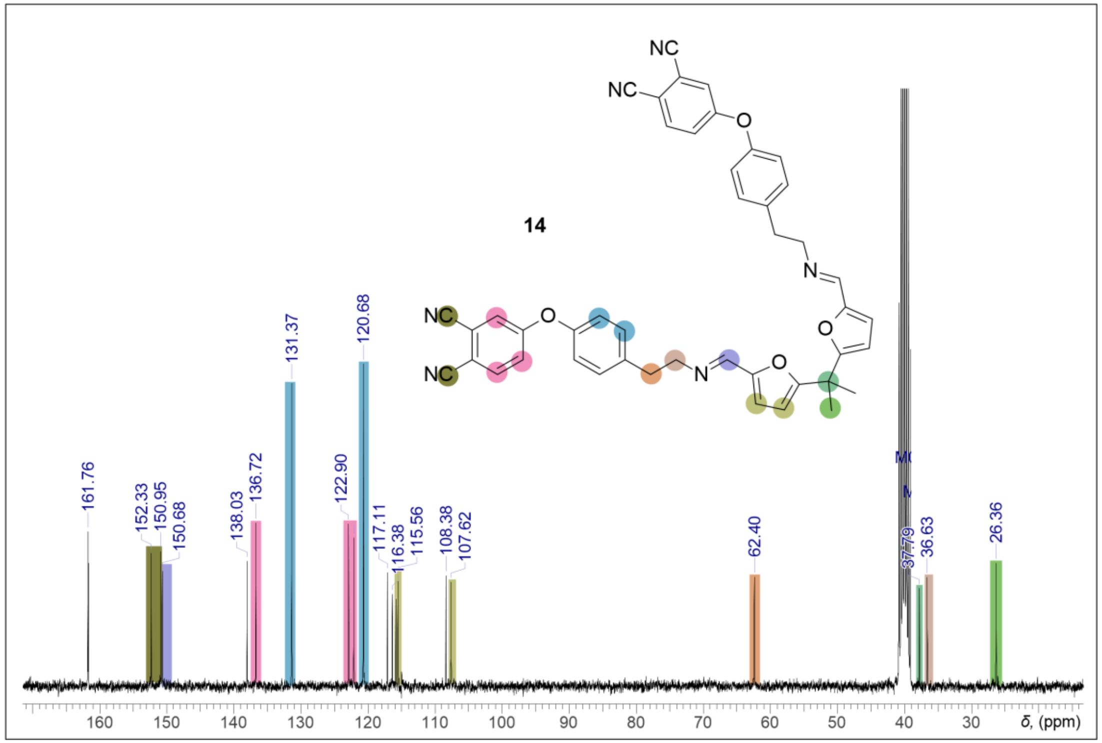

Figure S 38. ^{13}C NMR spectrum of BFA-Ty-PN (**14**).

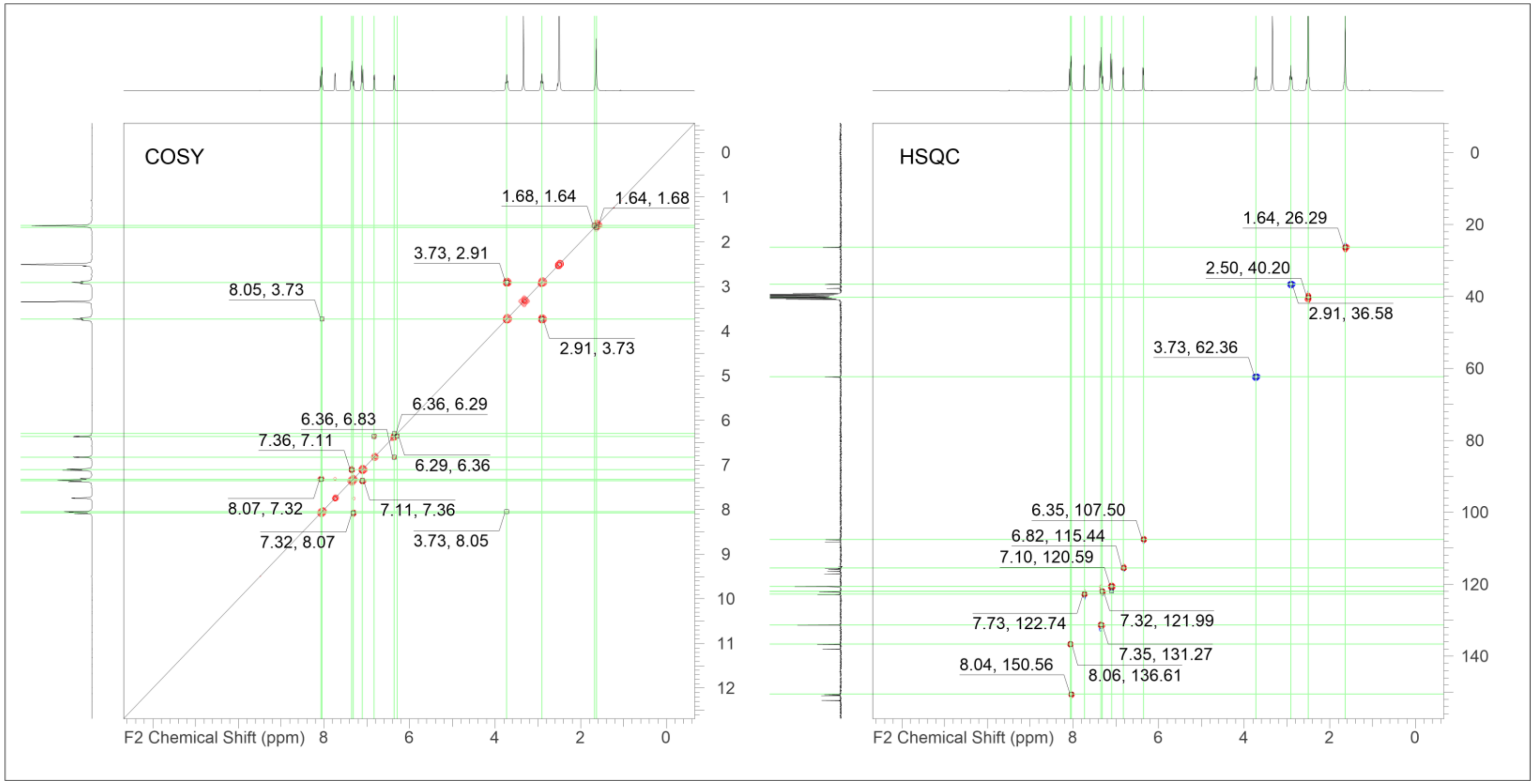

Figure S 39. COSY and HSQC spectra of BFA-Ty-PN (**14**).

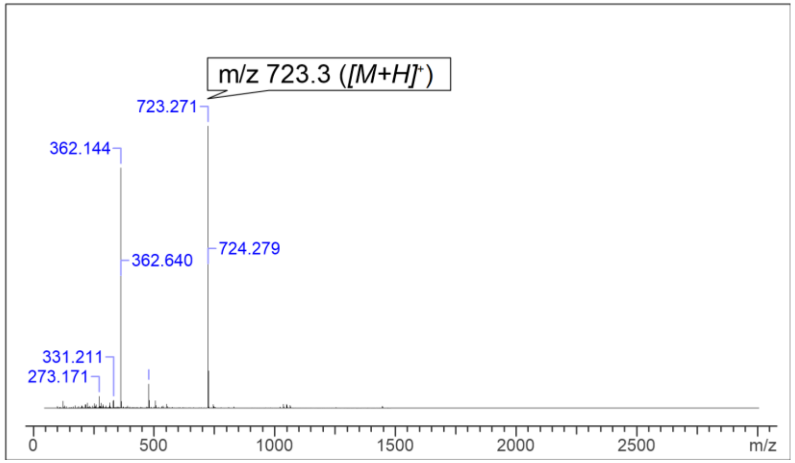

Figure S 40. ESI-MS spectrum of BFA-Ty-PN (**14**).

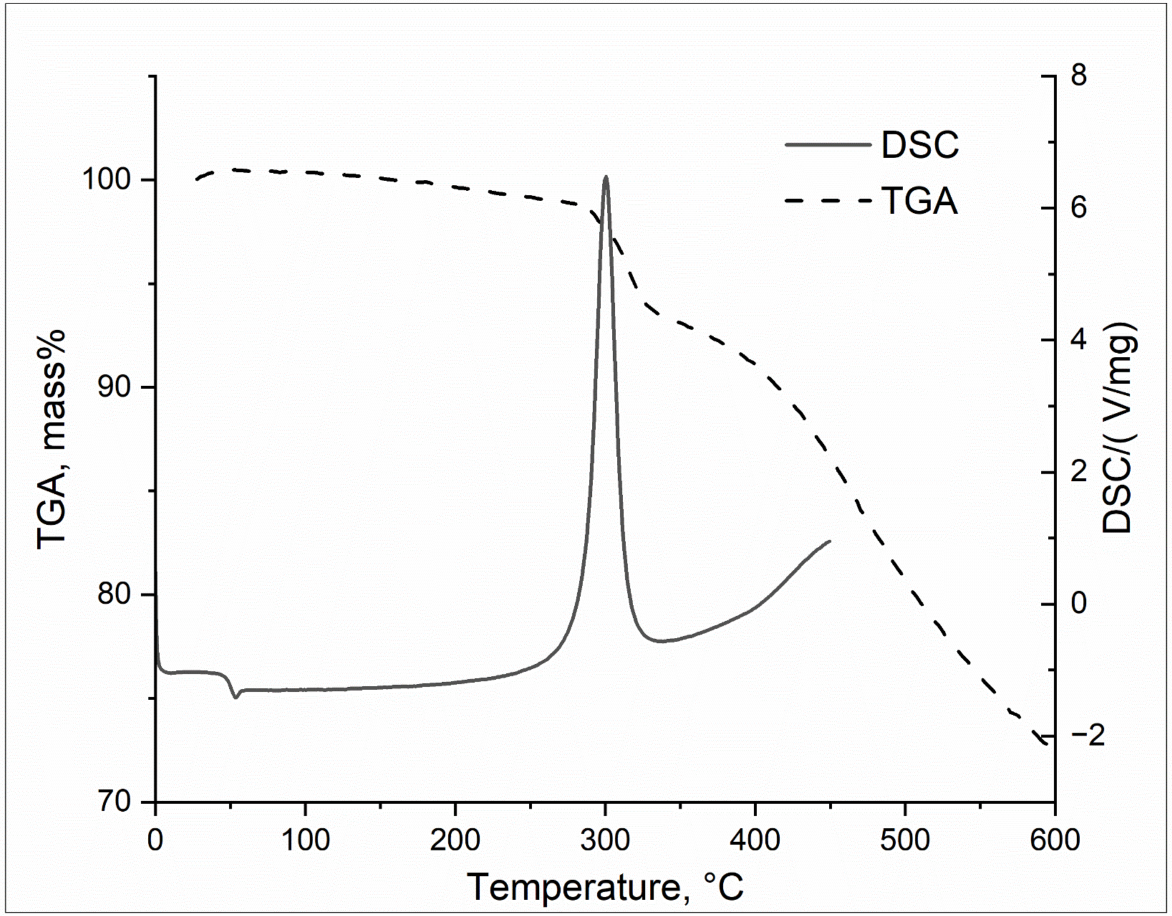

Figure S 41. TGA and DSC of BFA-Ty-PN (**14**).

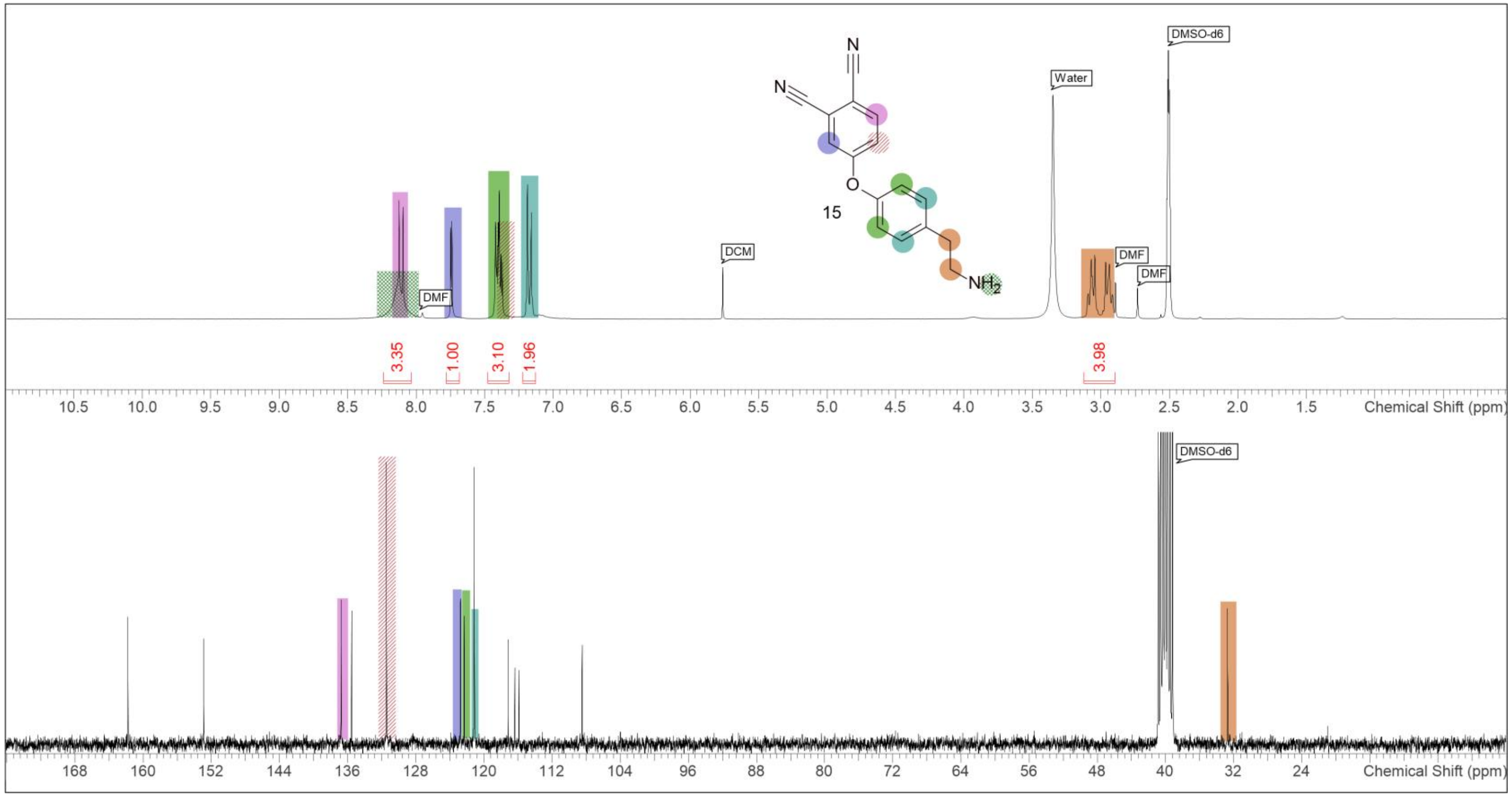

Figure S 42. ^{1}H and ^{13}C NMR spectra of TyPN (**15**).

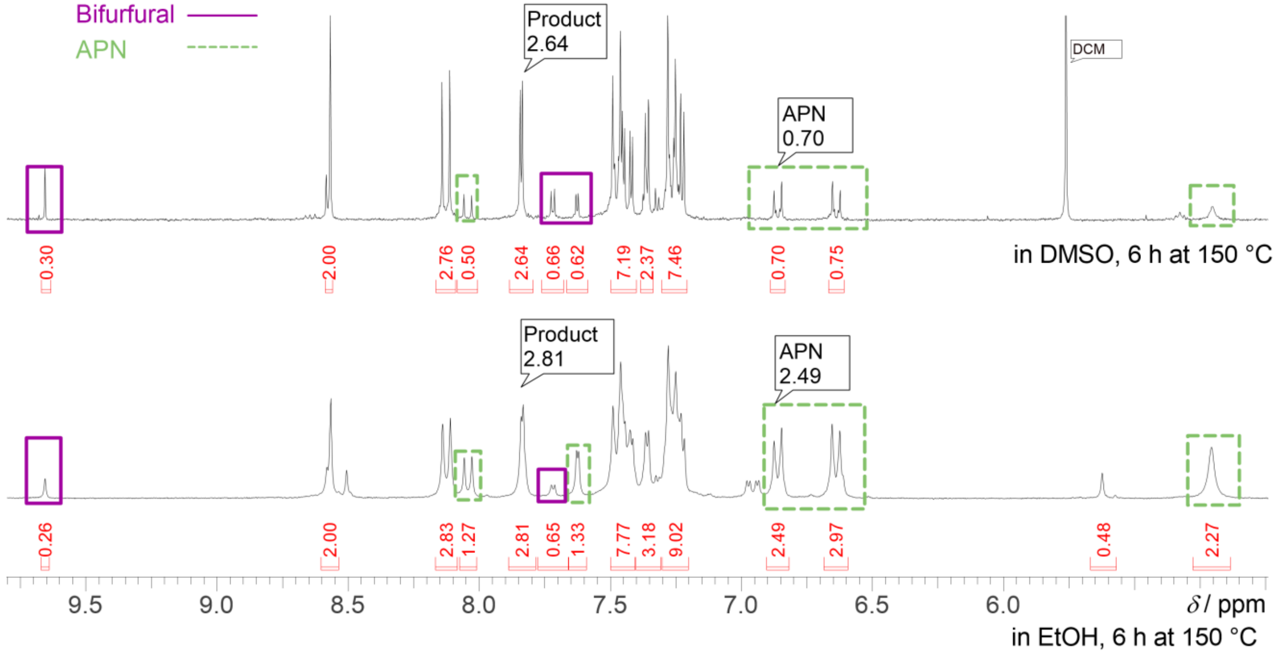

Figure S 43. ^{1}H NMR spectra of reactions between APPN and BF in DMSO (upper) and in ethanol (lower).

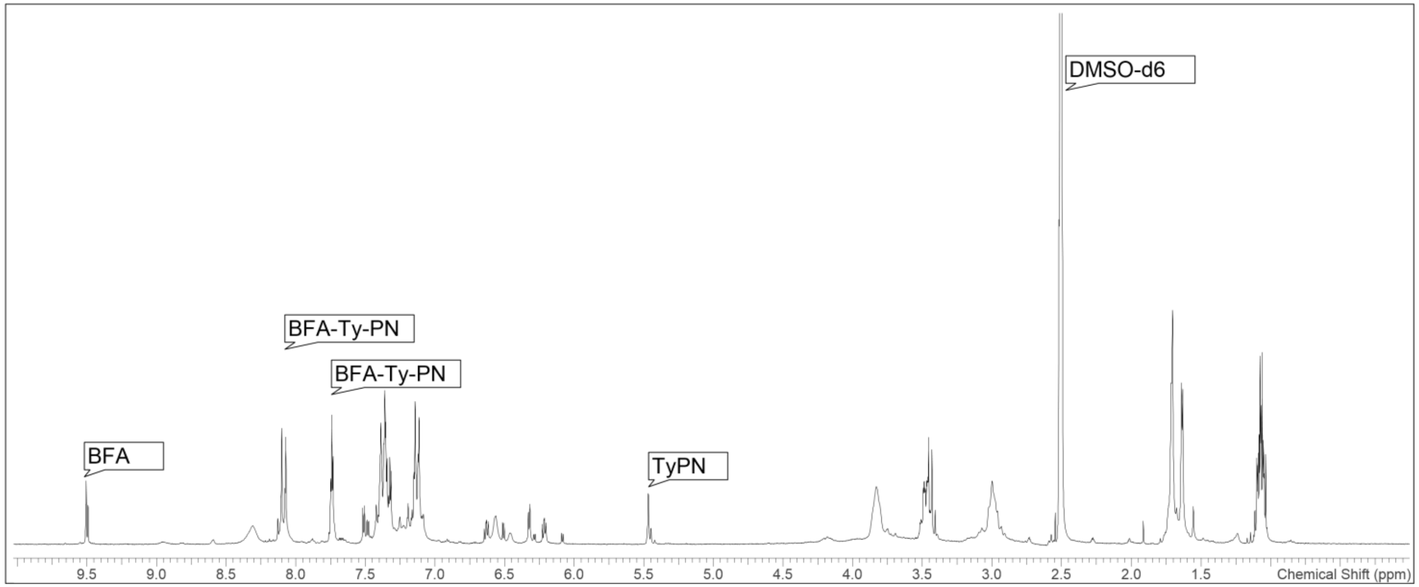

Figure S 44. ^{1}H NMR spectrum of reactions between BFA and BFA in ethanol.

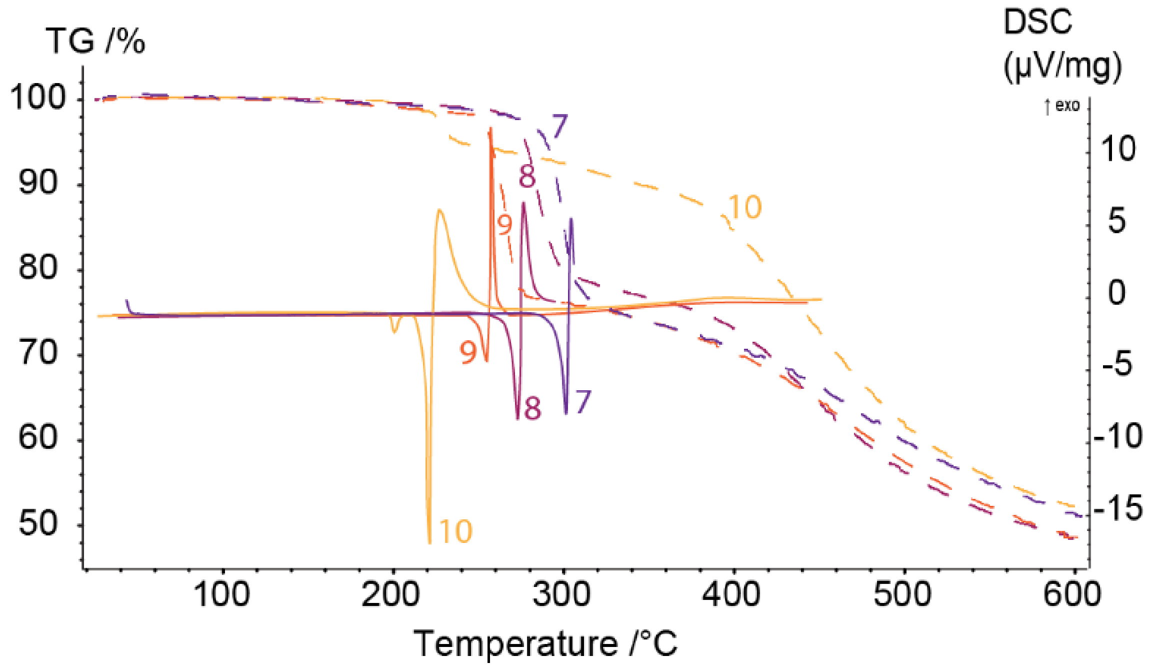

Figure S 45. DSC (solid) and TGA (dashed) of the monomers **7**-**10**.

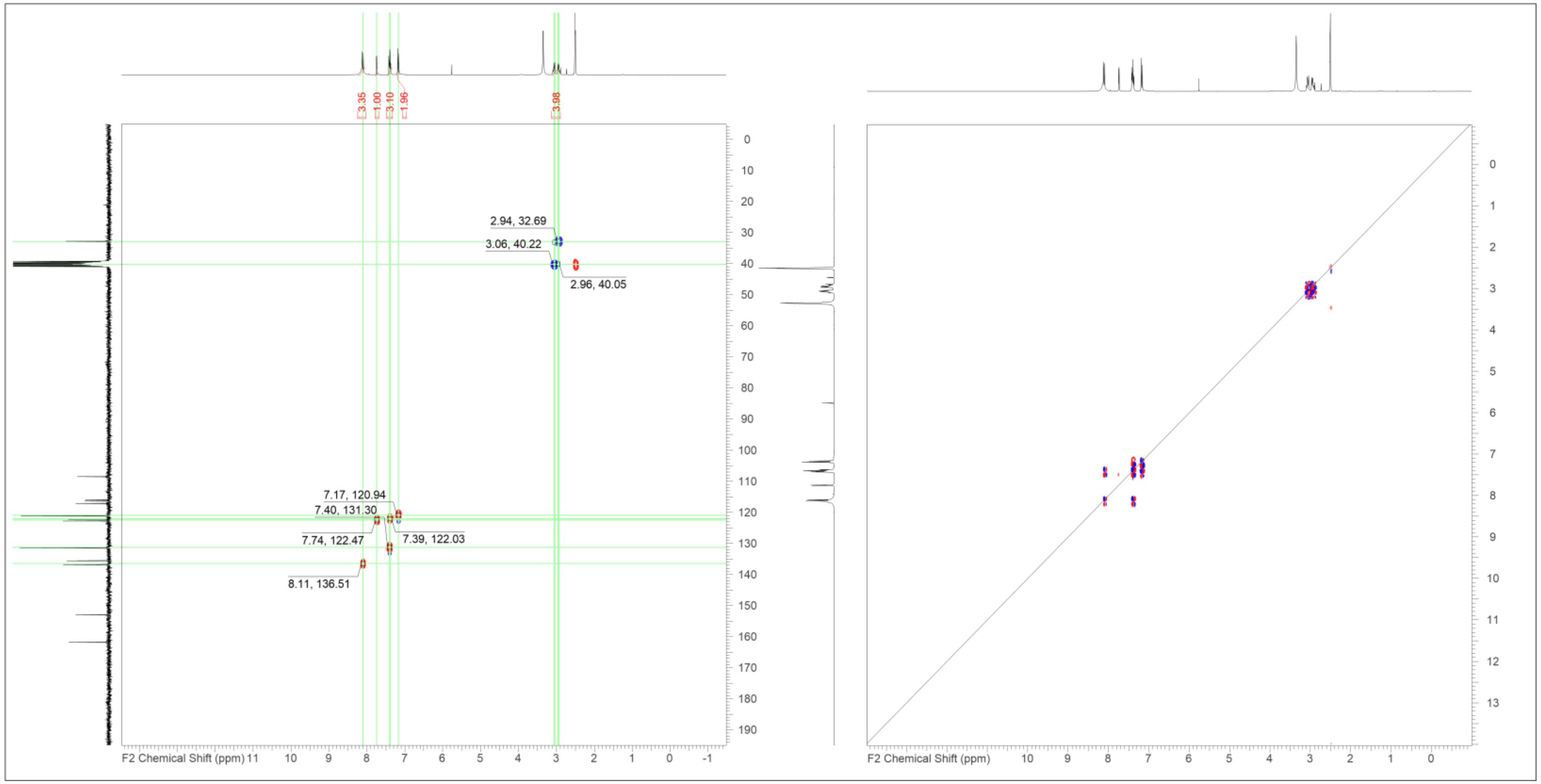

Figure S 46. COSY and HSQC spectra of BFA-Ty-PN (**14**).

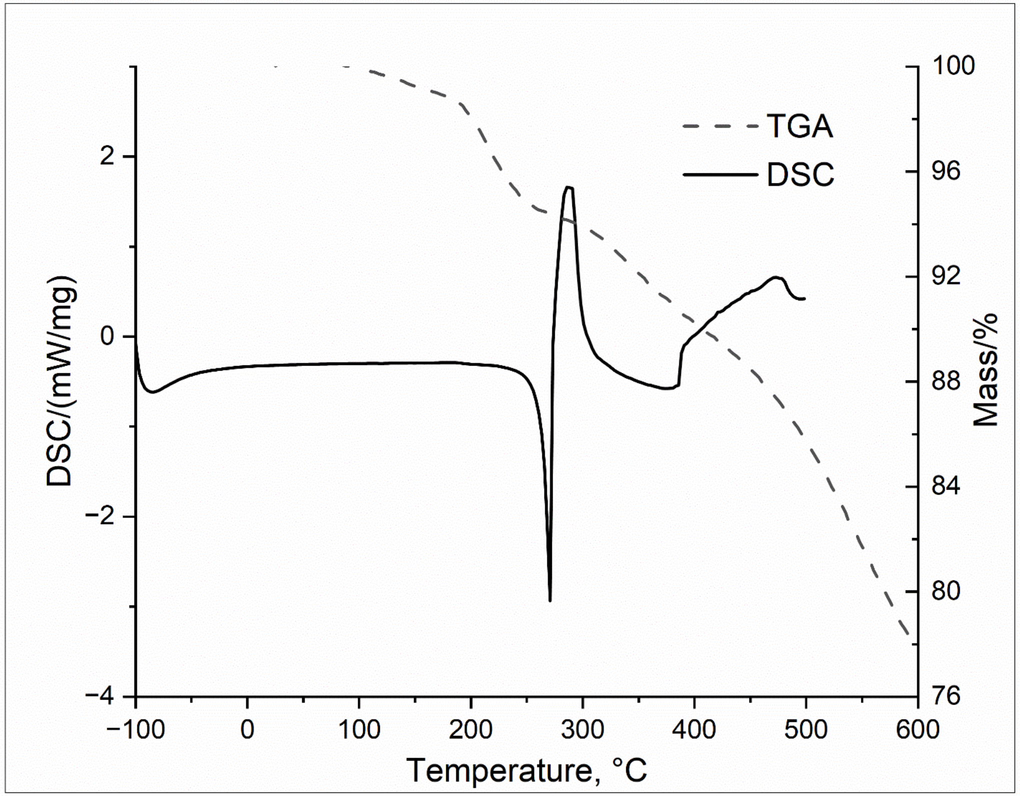

Figure S 47. TGA and DSC of Ty-PN (**15**).

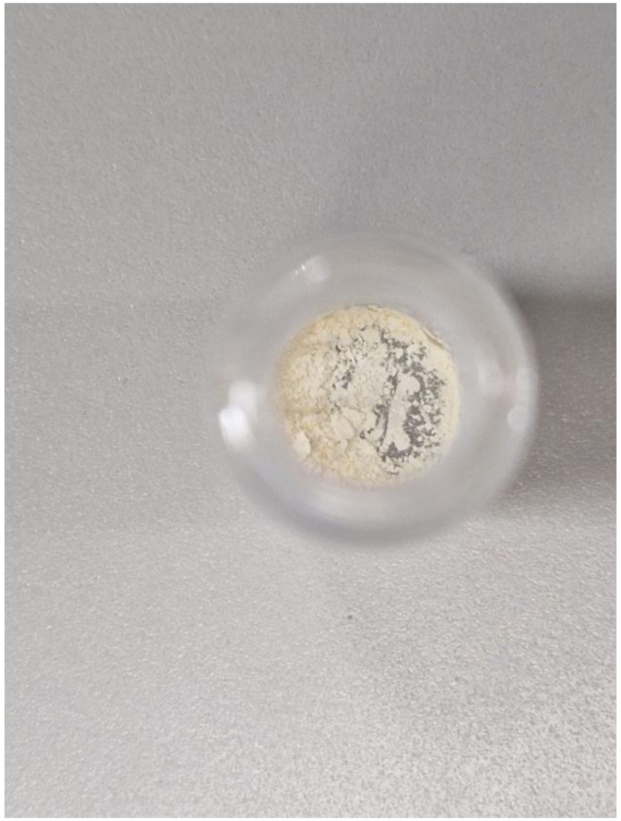

Figure S 48. TyPN (**15**) after recrystallisation.

References

[1] H. Sheng, X. Peng, H. Guo, X. Yu, K. Naito, X. Qu, Q. Zhang, "Synthesis of high performance bisphthalonitrile resins cured with self-catalyzed 4-aminophenoxy phthalonitrile," *Thermochim. Acta.* **2014**, *577*, 17–24.

[2] M. Chen, X. He, J. Lv, H. xiao, W. Tan, Y. Wang, J. Hu, K. Zeng, G. Yang, "A new bio-based thermosetting with amorphous state, sub-zero softening point and high curing efficiency," *Polymer (Guildf)* **2023**, *264*, 125518.

[3] T.-J. Lu, J.-F. Yang, L.-J. Sheu, "An Efficient Method for the Acetalization of .alpha.,.beta.-Unsaturated Aldehydes," *J. Org. Chem.* **1995**, *60*, 2931–2934.

[4] Y. Tachibana, S. Hayashi, K. Kasuya, "Biobased Poly(Schiff-Base) Composed of Bifurfural," *ACS Omega* **2018**, *3*, 5336–5345.

[5] V. Gaitonde, K. Lee, K. Kirschbaum, S. J. Sucheck, "Bio-based bisfuran: synthesis, crystal structure, and low molecular weight amorphous polyester," *Tetrahedron. Lett.* **2014**, *55*, 4141–4145.

[6] R. Ditchfield, W. J. Hehre, J. A. Pople, "Self-Consistent Molecular-Orbital Methods. IX. An Extended Gaussian-Type Basis for Molecular-Orbital Studies of Organic Molecules," *J. Chem. Phys.* **1971**, *54*, 724–728.

[7] G. A. Petersson, A. Bennett, T. G. Tensfeldt, M. A. Al-Laham, W. A. Shirley, J. Mantzaris, "A complete basis set model chemistry. I. The total energies of closed-shell atoms and hydrides of the first-row elements," *J. Chem. Phys.* **1988**, *89*, 2193–2218.

[8] R. Krishnan, J. S. Binkley, R. Seeger, J. A. Pople, "Self-consistent molecular orbital methods. XX. A basis set for correlated wave functions," *J. Chem. Phys.* **1980**, *72*, 650–654.

[9] T. H. Dunning, ""Gaussian basis sets for use in correlated molecular calculations. I. The atoms boron through neon and hydrogen," *J. Chem. Phys.* **1989**, *90*, 1007–1023.

[10] R. A. Kendall, T. H. Dunning, R. J. Harrison, "Electron affinities of the first-row atoms revisited. Systematic basis sets and wave functions," *J. Chem. Phys.* **1992**, *96*, 6796–6806.
